# Renewable Beta‐Elemene Based Cyclic Carbonates for the Preparation of Oligo(hydroxyurethane)s

**DOI:** 10.1002/cssc.202201123

**Published:** 2022-07-25

**Authors:** Cristina Maquilón, Arianna Brandolese, Christian Alter, Claas H. Hövelmann, Francesco Della Monica, Arjan W. Kleij

**Affiliations:** ^1^ Institute of Chemical Research of Catalonia (ICIQ) The Barcelona Institute of Science and Technology (BIST) Av. Països Catalans 16 43007 Tarragona Spain; ^2^ BASF Coatings GmbH 48165 Münster Germany; ^3^ Catalan Institute of Research and Advanced Studies (ICREA) Pg. Lluis Companys 23 08010 Barcelona Spain; ^4^ Current affiliation: Dipartimento di Biotecnologie e Scienze della Vita Università degli Studi dell'Insubria Via J. H. Dunant 3 21100 Varese Italy

**Keywords:** amine reagents, beta-elemene, carbon dioxide, cyclic carbonates, polyurethanes

## Abstract

Conversion of β‐elemene into new β‐elemene dicarbonates through epoxidation and halide salt‐catalyzed CO_2_ cycloaddition reactions is reported. Step‐growth polyaddition of this dicarbonate to five different, commercial diamines was investigated under neat conditions at 150 °C yielding non‐isocyanate‐based low molecular weight oligo(hydroxyurethane)s with 1.3≤*M_n_* ≤6.3 kDa and 1.3≤*Ð*≤2.1, and with glass transition temperatures ranging from −59 to 84 °C. The preparation of one selected polyhydroxyurethane material, obtained in the presence of Jeffamine® D‐2010 was scaled‐up to 43 g. The latter, when combined in a formulation using Irgacure® 2100 and Laromer® LR 9000 allowed the preparation of coatings that were analyzed with several techniques showing the potential of these biobased oligourethanes towards the preparation of commercially relevant materials.

## Graphical abstract

**Naturally**: Biobased β‐elemene was probed as a renewable precursor for the synthesis of non‐isocyanate based polyurethane coatings. Conversion of this terpene into its bis‐cyclic carbonate was examined, followed by polycondensation with commercially relevant diamines thereby providing oligourethanes as pre‐polymers. One of these was tested extensively and found to be potentially suitable as a starting point for new coating formulations using a dual curing approach.

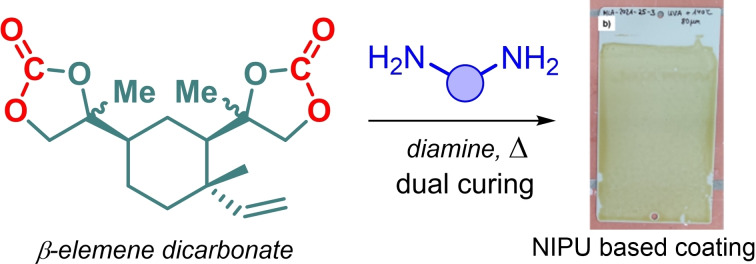

## Introduction

**Scheme 1:**
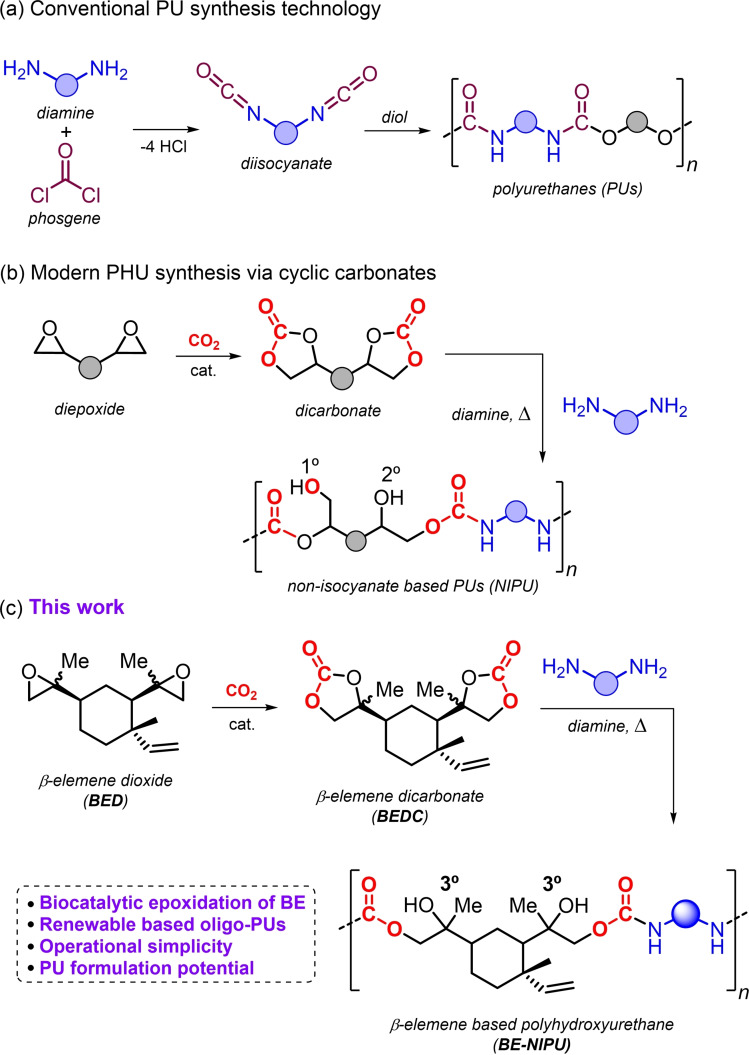
Comparison between conventional PU synthesis (a), alternative NIPU synthesis (b), and β‐elemene‐based PUs presented in this work (c). For simplicity, diisocyanates and diols are shown here as an exemplary case.

Polyurethanes (PUs)[^1a^, ^1b^, ^1c^] represent one of the most industrially important classes of polymeric materials. The PU demand is increasing every year with an estimated European market size of around $26.24 billion by 2024.^[1d]^ Currently, PUs are employed in a wide range of consumer applications such as foams, insulators, paints, liquid coatings, sealants, fibers and elastomers, among others.[^1a^, ^1b^, ^1c^, ^2^] PU production technology is based on polyaddition reactions between isocyanates and polyols (Scheme 1a). Despite the mature and efficient nature of this technology, there are several drawbacks. Isocyanates are prepared by reaction of amines with highly toxic and dangerous phosgene in an energy‐intensive process. It further releases hydrogen chloride as by‐product, while exposure to isocyanates negatively affects human health.^[3]^ The irreversible reaction between isocyanates and water produces CO_2_, which is conveniently used to produce foam, but generates problems in storage and handling of isocyanate reagents.

The production of commonly employed polyols, such as polyethylene oxide and polypropylene oxide,^[4]^ relies on the use of fossil fuel‐based feedstock and requires handling of dangerous and harmful chemicals such as ethylene oxide. Consequently, the identification of new synthetic routes based on safer reactions and chemicals is a topic of great interest in green chemistry that has spurred the community to identify and develop more sustainable routes for PUs. In this respect, the creation of non‐isocyanate‐based polyurethanes, abbreviated as NIPUs, has become an active area of research.^[5]^

Such NIPUs can be prepared through the polyaddition reaction between di/multi‐cyclic carbonate reagents and diamines (Scheme 1b). This route has become more and more appealing due to the development of efficient and sustainable methods for the production of carbonates from CO_2_ and epoxides.^[6]^ Moreover, many research efforts are devoted to the sustainable production of (di)amines from renewable sources^[7]^ since they are useful intermediates in many synthetic processes. NIPUs obtained through this polyaddition route are typically referred to as poly(hydroxyurethane)s (PHUs) because of the presence of primary (1°) and secondary (2°) alcohol groups along the main polymer chain with generally the secondary OH groups being more abundant. The presence of hydroxyl groups enables an additional intra‐ and inter‐molecular hydrogen‐bond network. As a result, an increase in polarity, thermal stability, adhesion properties and water uptake of these types of PUs is feasible. These improved properties have advanced the discovery and development of new materials.^[8]^

In spite of the high attractiveness of this new route towards the formation of NIPUs, the number of renewable, biosourced monomers in NIPU synthesis remains highly limited.^[9]^ Biobased macromolecules are attractive and considered more benign alternatives to conventional polymers with often a lower carbon footprint. Therefore, the use of biosourced monomers can give impetus for developing alternative and green NIPU derived materials especially in those cases where the monomers combine features such as renewability, low cost and modularity.

**Scheme 2:**
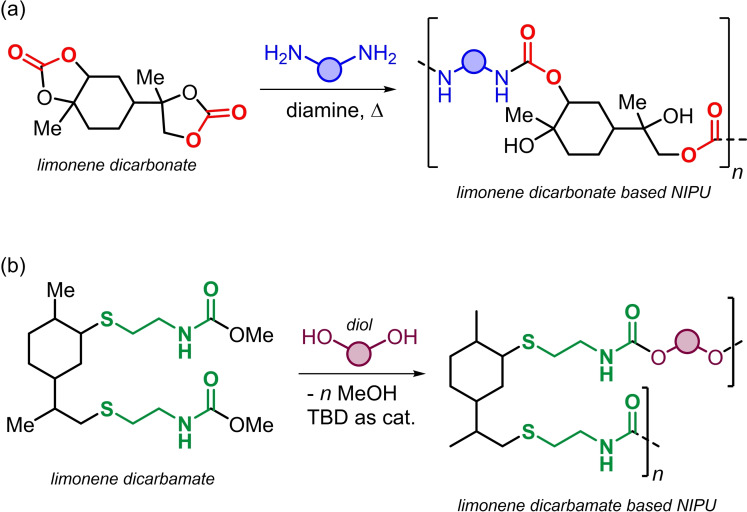
Previously reported (a) limonene‐dicarbonate based NIPU, and (b) limonene dicarbamate based NIPU synthesis.

Over the last decade, terpenes have been recognized as useful and functional bio‐monomers for the preparation of polymeric materials.^[10]^ Several types of terpene‐based polymers have been developed demonstrating the vast potential of terpene‐derived monomers for the creation of sustainable material and product development guiding by principles of a circular economy and chemistry.^[11]^ So far, very few terpene‐based cyclic carbonates have been used in NIPU synthesis. Limonene‐based NIPUs have been reported through different synthetic approaches (Scheme 2).^[12]^ The group of Mülhaupt reported the preparation of NIPUs by treatment of limonene dicarbonate with diamines thereby obtaining oligomers with glass transition temperatures (*T*_g_'s) ranging from 33 to 62 °C (Scheme 2a).^[12a]^ Brittle NIPU based thermosets were obtained in the presence of polyfunctional amines. More recently, the influence of the limonene dicarbonate purity on the final material properties was studied by the same group.^[12b]^ Different from the work of Mülhaupt, Firdaus and Meier described the synthesis of biobased NIPUs through polycondensation of renewable diols and limonene dicarbamate (Scheme 2b).^[12c]^ In this latter case, *M*_n_ of up to 12.6 kDa were achieved and semi‐crystalline samples were obtained in some cases exhibiting melting temperatures (*T*_m_'s) as high as 69 °C.

In order to advance the portfolio of biobased NIPUs obtained from more rigid precursors, we envisioned that functional terpene‐derived cyclic carbonates (i.e., β‐elemene dicarbonate, BEDC; Scheme 1c) would be attractive. β‐elemene (BE) is a natural sesquiterpene commonly extracted from ginger root but can also be found in citrus fruit. BE can be made available on multi‐ton scale through a fermentation process from sugar.^[13]^ Here, we describe the preparation of new NIPU oligomers based on BE. These oligomers were used as a starting point for coating formulations using both UVA and thermal curing. The present results help to establish new potential for biobased terpene scaffolds as key components of NIPU‐derived materials.

## Results and Discussion

### β‐Elemene dicarbonate synthesis

Recently, we described the synthesis of β‐elemene epoxides via standard epoxidation methods using *meta*‐chloroperbenzoic acid (*m*CPBA) as a reagent.^[14]^ This method allows to scale up the synthesis of β‐elemene diepoxide (BED) to multigram quantities (up to 8 g, see the Supporting Information) and therefore securing access to larger amounts of its dicarbonate, enabling a practical synthesis of the respective oligourethanes. In order to provide a more sustainable approach towards BED, BE was epoxidized under heterogeneous batch conditions using Novozym® 435 as a biocatalysts and H_2_O_2_ (30 % v/v in water, 3.0 equiv.) as a more benign oxidant in EtOAc. This epoxidation is carried out under mild temperature conditions (50 °C) providing BED with >95 % selectivity (Supporting Information) and giving access to gram quantity of the bis‐epoxide.^[15]^

With the synthesis of BED being covered in terms of quantities and conditions, we explored the reaction of this bis‐epoxide with carbon dioxide in the presence of the commercial halide salts tetrabutylammonium chloride (TBAC), tetrabutylammonium bromide (TBAB) or bis(triphenylphosphine)iminium chloride (PPNCl) under reaction conditions similar to those used with other bulky (bis)epoxides (100 °C, 40 bar of CO_2_).^[16]^ After 24 h, β‐elemene BE can be fully converted into a mixture of β‐elemene *mono*‐carbonate (BEMC) and β‐elemene *di*‐carbonate (BEDC) products (Table 1, entries 1–3), with the use of PPNCl giving a somewhat higher BEDC/BEMC ratio (entry 3; BEDC/BEMC=53 : 47). Both BEMC and BEDC can be easily separated from each other via column chromatography allowing to identify and fully characterize both carbonate products by NMR, HRMS and FTIR analyses (see Supporting Information).

**Table 1:** Carbon dioxide cycloaddition to β‐elemene dioxide toward β‐elemene dicarbonate (BEDC).

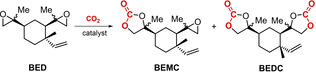				
Entry^[a]^	Catalyst [mol %]^[b]^	Temperature [°C]	Time [h]	BEDC/BEMC [%]^[c]^
1	TBAB, 1.5	100	24	38/62
2	TBAC, 1.5	100	24	39/61
3	PPNCl, 1.5	100	24	53/47
4	PPNCl, 1.5	100	48	78/22
5	PPNCl, 1.5	100	72	70/30
6^[d]^	PPNCl, 1.5	100	72	91/9
7^[d]^	PPNCl, 2.0	100	72	>99/1
8^[e]^	PPNCl, 2.0	100	72	80/20
9^[e]^	PPNCl, 2.0	115	72	86/14
10^[e]^	PPNCl, 2.0	130	72	>99/1
11^[f]^	PPNCl, 2.0	130	72	>99/1

[a] Reaction conditions: BED=50 mg (2.1×10^−4^ mol), 40 bar CO_2_, neat. Conversion of β‐elemene dioxide always >99 %, determined by ^1^H NMR. [b] With respect to epoxide groups. [c] Determined by ^1^H NMR (CDCl_3_) by integration of epoxide/carbonate/olefin signals. [d] BED=200 mg (8.5×10^−4^ mol). [e] BED=1.0 g (4.2×10^−3^ mol). [f] BED=4.0 g (1.7×10^−2^ mol).

The reaction time was extended to 72 h while using PPNCl, reaching a BEDC‐to‐BEMC ratio of 91/9 (entries 4–6). Complete conversion of BED into BEDC was achieved by using a catalyst loading of 2 mol % (entry 7, Table 1). Increasing the initial quantity of BED to 1.0 g resulted in a significantly lower BEDC/BEMC ratio, likely due to more difficult CO_2_ diffusion to the reactive sites, and a reaction temperature of 130 °C was necessary to reach quantitative substrate conversion (entries 8–10, Table 1). Under these latter conditions, the reaction could be scaled up to 4.0 g of starting material without affecting the conversion (entry 11, Table 1) and thus providing multi‐gram quantities of BEDC.

BEDC is obtained as a viscous oil after column chromatography, which was needed to remove the halide catalyst. NMR characterization revealed the formation of several diastereoisomers in line with the stereochemical mixture obtained for BED (see the Supporting Information). Attempts to obtain crystals suitable for X‐ray analysis were unsuccessful, however precipitation of one enriched diastereoisomer as a white solid was possible.^[17]^

### Synthesis of urethane model compounds

**Scheme 3:**
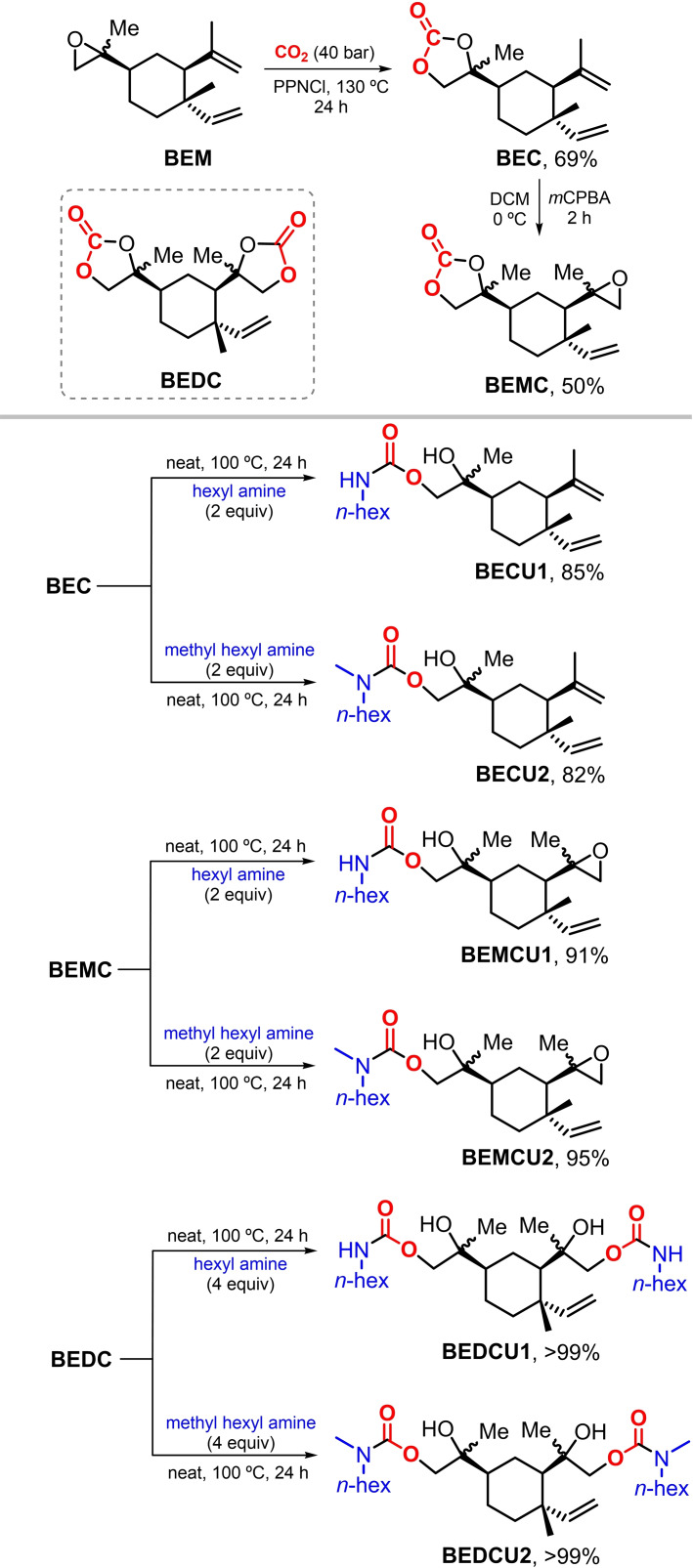
Urethane derivatives prepared from carbonates BEC, BEMC and BEDC, and primary/secondary mono‐amines.

In order to examine the reactivity of cyclic carbonates derived from β‐elemene, we decided to use the three carbonate species depicted in Scheme 3 including BEC, BEMC and BEDC, and monoamines *n*‐hexylamine and *N*‐methylhexyl amine. The BEC (69 % yield) was produced from the known mono‐epoxide BEM^[14]^ using PPNCl as catalyst under neat conditions. Subsequent epoxidation of BEC using *m*CPBA in DCM at 0 °C afforded the mono‐carbonate‐mono‐epoxide BEMC in 50 % isolated yield. The latter preparation can be seen as a useful and more practical alternative to the isolation of BEMC from the respective mixtures of carbonates reported in Table 1. These mixtures allow only low yields of this mono‐carbonate under the conditions reported. BEMC represents an interesting trifunctional building block/monomer comprising of a cyclic carbonate, epoxide and olefin functional groups which may allow for orthogonal functionalization approaches and polymer engineering.

With these three carbonates in hand, we investigated their reactivity towards a primary and two secondary amines (Scheme 3, lower part). Under neat conditions, all three carbonates could be quantitatively transformed into their respective mono‐ (BECU1, BECU2, BEMCU1 and BEMCU2) and di‐urethanes (BEDCU1 and BEDCU2) in (expectedly) good isolated yields of up to >99 %. While the reactions between the carbonates and hexyl amine or *N*‐methylhexyl amine proceeded smoothly, the use of *N*‐(*n*‐butyl)benzyl amine did not provoke any conversion of these carbonates between 100–130 °C, which may be rationalized by steric effects. It should be noted that the cyclic carbonates ring‐opening was regioselective in all cases, leading to hydroxy‐urethane structures having tertiary OH groups.

The formation of urethane (or carbamate) linkages could be easily deduced from both the NMR and IR analyses while comparing their spectra with the ones from the respective cyclic carbonates. The latter display highly characteristic absorptions for the carbonate fragments at 1783–1791 cm^−1^, whereas the carbamates of Scheme 3 display peaks at 1682–1698 cm^−1^. These latter values are well within the range typically noted for the urethane linkages of NIPUs,^[8]^ and the collected spectroscopic data offers a reference point for the NIPU formation discussed below.

The ^1^H NMR spectra of the urethanes were less clear due to the presence of various stereoisomers in the products. In order to get more instructive spectroscopic data, a reaction was performed using a precipitated sample of BEDC (see above) and *n*‐hexyl‐amine. With this purified sample of BEDC, the ^1^H NMR spectrum of the derived di‐urethane BEDCU1 displayed the presence of virtually a single species (see the Supporting Information).

### Polyaddition of BEDC and diamines

**Scheme 4:**
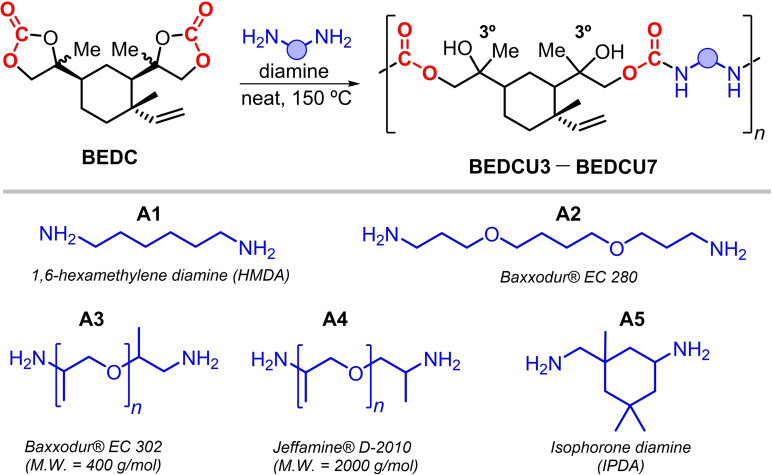
Synthesis of β‐elemene based oligo(hydroxyurethane)s BEDCU3 to BEDCU7 using diamines (DA) A1–A5.

Next, we examined the polyaddition between various diamines and BEDC (Scheme 4). These reactions were performed under solvent‐free conditions at 150 °C with a stoichiometric (1 : 1 ratio) amount of both reagents.

Five different diamines were used (Table 2). Commercially relevant linear and cyclic substrates were employed, and also α‐ and β‐methyl substituted amines were probed. The molecular weights (number average molecular weights, *M*_n_), ranging from 1.3 to 6.3 kg mol^−1^, point at the formation of oligomeric NIPU species. These results were anticipated as Mülhaupt and co‐workers showed that similar reactivity profiles are observed when using limonene dicarbonate as a precursor.[^12a^, ^12b^] The two epoxides present in BED have distinct reactivity, with one being sterically more hindered. This is clearly noticed in the preparation of BEDC, and at lower reaction temperatures ( ≤100 °C, see also Table 1, entries 1 and 2) the mono‐carbonate BEMC is the major reaction product. Therefore, it can be assumed that the carbonate ring positioned between the terminal olefin and second carbonate substituent in BEDC will have lower reactivity upon ring‐opening by the diamine reagent. Not surprisingly, the higher the molecular weight and the more flexible the diamine reagent, the less viscous the NIPU that is produced. Along this tendency, similar notions are made for the *T*_g_ values with temperatures ranging from −59 to 84 °C for the lowest and highest viscosity of the prepared NIPUs.

**Table 2:** Properties of β‐elemene‐based BEDCUs.

Entry^[a]^	BEDCU/DA	IR^[b]^ [cm^−1^]	*M*_n_ [kDa]^[c]^	*Ð* ^[c]^	*T*_g_ [°C]^[d]^	*T*_d_ [°C]^[e]^
1	BEDCU3, A1	1692	1.6	1.5	−6	243
2	BEDCU4, A2	1692	1.8	1.5	−26	248
3	BEDCU5, A3	1690	2.0	1.6	−25	256
4	BEDCU6, A4	^[f]^	6.3	2.1	−59	310
5	BEDCU7, A5	1695	1.3	1.3	84	247

[a] Reaction conditions: BEDC=1.0 g (3.1×10^−3^ mol), diamine=3.1×10^−3^ mol, T=150 °C, t=48 h. [b] Values correspond to the urethane linkages. [c] Obtained from GPC analysis in DMAc containing 1 % TFAc and 0.5 % LiBr at 40 °C, and calibrated with PMMA standards. [d] Obtained by DSC analysis with a scan rate of 10 °C min^−1^. [e] Onset decomposition temperature obtained by TGA under nitrogen. [f] Too weak carbamate absorption, a weak and broad absorption band noted at *ν* ≈3300–3600 cm^−1^ is ascribed to the presence of NH/NH_2_/tertiary OH groups.

Both the NMR and IR spectra of the new NIPUs (BEDCU3‐7) showed clear evidence for the presence of unreacted carbonate and amine groups (see the Supporting Information) and therefore they should be regarded as pre‐polymers potentially useful as chain extenders thereby increasing the bio‐content of NIPU formulations. Thus, further cross‐linking reactions were assessed. Since BEDCU6 showed the lowest viscosity, we decided to scale up its synthesis starting with 6 g of BEDC (with 43 g of final hydroxy‐urethane product) in order to have enough starting material for various cross‐linking experiments.

### Initial crosslinking tests

A distinct feature of NIPUs generated from the aminolysis of polycarbonates is the presence of hydroxyl groups in the final product. In addition, in the compounds BEDCU3–7 an additional pendent double bond is also present in the repeat units of the oligomers. These double bonds could be used for crosslinking through well‐established radical‐mediated thiol‐ene chemistry creating (ideally) dithioether linkages between the polymer chains. Consequently, the network created in this way would represent a hard and dry film suitable for testing of various physical properties. A commonly used crosslinking agent is pentaerythritol tetrakis(3‐mercapto‐propionate) (PEMTP) that features four thiol moieties. Thus, we explored suitable conditions for the thermal curing of BEDCU6 by PEMTP in the presence of 2,2′‐azobis(2‐methylpropionitrile) (AIBN) as catalyst and performing these experiments in an oven for 40 min. Double bond (C=C) to thiol (SH) equivalents ranging from 1 : 0.8 to 1 : 2 were considered, and temperatures ranging from 100 °C to 140 °C were screened. These thermal curing attempts, however, only gave sticky layers and hence were not suitable for physical tests even when using higher relative amounts of tetra‐thiol.

Thus, we decided to try a different, bifunctional cross‐linker [i.e., ethylene glycol bis(3‐mercaptopropionate), GDMP] since the steric requirements of PEMTP probably affected the crosslinking rate of BEDCU6. The latter was thus treated at 140 °C during 40 min in the presence of 2 wt % of AIBN, and with different ratios (1 : 0.8, 1 : 1 and 1 : 1.2) between the C=C bonds of BEDCU6 and the thiol groups of GDMP. Unfortunately, the resulting layers were even more sticky compared to the ones obtained with PETMP produced at a 1 : 1.2 ratio. The difference in the physical state of the reaction mixture before and after curing with the thiol reagents suggested that a certain degree of crosslinking had indeed taken place. At this stage, we believed that the amount of double bonds in BEDCU6 is probably too low (due to the high molecular weight of Jeffamine® 2010) to generate enough thiol bridges, and thus resulting into macromolecular structures that are not suitable as coatings. As a possible solution, we then considered the use of dual curing by addition of an oligomeric additive to increase the double bond density (see below).

### Dual curing crosslinking reactions and physical tests

**Scheme 5:**
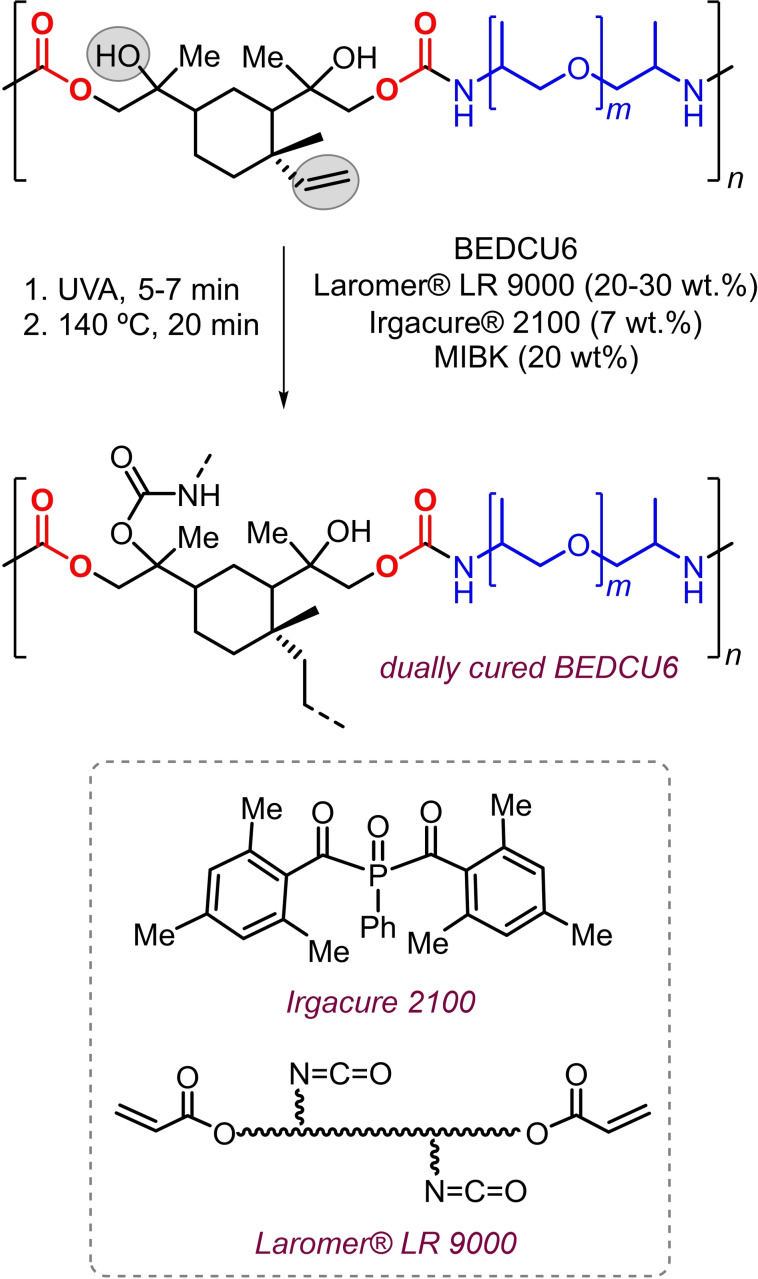
Dual curing of BEDCU6 in the presence of Irgacure® 2100 (photo‐initiator) and Laromer® LR 9000 as an isocyanate acrylate curing agent.

Dual cure processes involve two types of reactions taking place to cure a coating. In the present case, the dual curing process comprises of a photocatalytic coupling of the double bonds, and a thermal coupling of OH and NH_2_ groups with an isocyanate‐acrylate based oligomer added in order to increase the amount of double bonds present in the reaction mixture (Scheme 5).

First, we tried the curing of BEDCU6 by premixing 73 wt % of this oligourethane with 7 wt % Irgacure® 2100 (a photo‐initiator) dissolved in 20 wt % of methyl isobutyl ketone (MIBK) in a vial (formulation 1), and then applied a metallic panel employing a spiral squeegee or coating knife (doctor blade) with a certain thickness. The wet film was cured for 5 min under UV irradiation and subsequently heated for 20 min in an oven at 140 °C. Unfortunately, this curing procedure did not result in a dry coating, presumably due to the low density of double bond equivalents.

**Figure 1:**
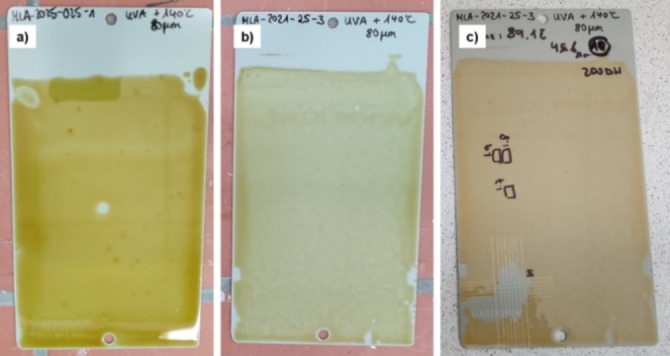
Comparison between coating samples obtained from BEDCU6+7 wt.% Irgacure® 2100 (a), BEDCU6+7 wt % Irgacure® 2100+30 wt % Laromer® 9000 (b), and coating (c) as prepared under (b) after the physical tests.

To overcome this limitation, we considered the co‐addition of a polyacrylate with reactive isocyanate groups in its structure (i.e., Laromer® 9000). We screened different combinations of thickness, concentration of Laromer® 9000 and UV curing time, while keeping the wt % of solvent and photo‐initiator constant (formulations 2 and 3). Also, a test to examine Laromer® 9000 self‐curing was performed (formulation 4) to compare with the combinations of BEDCU6 with Laromer® 9000. Once the cured coatings were prepared following these above‐mentioned formulations, testing of their physical properties was performed using different panels. In Figure 1, selected cured coatings before and after the physical tests are shown.

All the results for the physical tests are gathered in Table 3. The self‐curing of BEDCU6 (formulation 1) did not result in a dry layer, and consequently it was not possible to perform any physical test on it (Table 3, entry 1) but the dryness test was possible for the other curing conditions (formulations 2–4) allowing for further evaluation. The gloss results showed no real dependency on the formulation type, obtaining rather similar results within a small range of 87–96 E. One of the variations applied in film preparation was the associated thickness of the coating knife. Two different coating knife sizes (viz., 60 μm and 80 μm) were used for the application of the mixture in the panel before the dual curing process. After the combined UV and thermal curing, the dry panels showed reproducible thickness of 24–25 μm and 50–57 μm when using the 60 μm and 80 μm coating knife, respectively. Cross‐cut tests revealed a large difference between formulations containing a combination of BEDCU6 and Laromer® 9000 (formulations 2 and 3) and the self‐curing of the latter (formulation 4), representing a class 5 and class 0, respectively, in this assay.

**Table 3:** Physical test results for the different curing conditions employed.

Entry^[a]^	Formulation^[b]^	Dryness	Gloss [GU]	Thickness [μm]	Cross‐cut	Buchholz hardness	MEK test [DR]
1	Form. 1 80 μm	Not okay	–	–	–	–	–
2	Form. 2 80 μm	Okay	87.7	50.9	5	2.2	50
3	Form. 2 60 μm	Okay	89.7	24.3	5	1.7	0
4	Form. 3 80 μm	Okay	89.1	50.5	5	1.8	200
5^[c]^	Form. 3 80 μm	Okay	88.2	56.7	5	1.8	75
6	Form. 3 60 μm	Okay	87.4	25.7	5	1.6	25
7	Form. 4 80 μm	Okay	96.4	57.5	0	1.3	200

[a] Curing conditions: 5 min UV curing followed by 20 min in an oven at 140 °C. [b] Form. stands for formulation. [c] 7 min UV curing followed by 20 min in an oven at 140 °C.

These data indicate that coatings produced with our pre‐polymer BEDCU6 were not adhering perfectly to the panel surface. Similarly, the Buchholz hardness test offers numbers from 1.6 to 2.2 for formulations 2 and 3 and a slightly lower result (1.3) for formulation 4. Finally, the MEK test was crucial to conclude that a coating with a larger amount of Laromer® 9000 and a thicker layer shows better results (entries 2–5). Formulation 3 employing an 80 μm coating knife turned out as good as formulation 4 in terms of solvent resistance (entry 4 vs. 6), and only in these two cases the coating was able to resist 200 DR.

## Conclusion

The results presented in this work demonstrate that a bis‐cyclic carbonate derived from β‐elemene (a natural terpene) allows for the formation of sterically challenging non‐isocyanate based oligourethanes (NIPUs) using commercially relevant diamines. Five different diamines were tested offering appreciable conversion of the carbonate groups, and modest molecular weights of up to 6.3 kg mol^−1^ and *Ð* values in the range 1.3–2.1. Proof of concept trials were performed to investigate the application of these oligomeric NIPUs serving as a starting point for coating formulations. The initial results show some degree of promise after applying a dual curing process. Subsequent testing of various physical properties points towards potential of renewable β‐elemene in devising new polyurethane formulations.

## Experimental Section

### Synthesis of BEC

β‐Elemene mono epoxide (BEM, 220 mg, 1.0 mmol) and PPNCl (13 mg, 0.02 mmol, 2.0 mol %) were weighed into a 10 mL Teflon vessel equipped with a magnetic stirring bar and placed into a 15 mL autoclave reactor. The reactor was closed, and the system pressurized with CO_2_ (10 bar) and vented three times. Then, the reactor was pressurized at the selected pressure of 40 bar and placed in a heating mantle set at 130 °C. After stirring for 24 h, the reactor was cooled down with an ice/water bath. Mesitylene (10 mol %) was added as an internal standard, and the reaction analyzed by ^1^H NMR (CDCl_3_) to determine the conversion and selectivity. The reaction mixture was purified by column chromatography (8 : 2 Hex:EtOAc, *R*_f_=0.30). Yield: 183 mg (0.69 mmol, 69 %). ^1^H NMR (500 MHz, CDCl_3_) δ 5.91–5.71 (m, 1H), 4.99–4.90 (m, 2H), 4.87 (p, *J*=1.6 Hz, 1H), 4.59 (ddt, *J*=2.7, 1.9, 0.9 Hz, 1H), 4.32 (dd, *J*=8.5, 2.6 Hz, 1H), 4.08 (dd, *J*=8.5, 1.2 Hz, 1H), 2.05–1.94 (m, 1H), 1.83–1.23 (m, 13H), 1.01 (s, 3H) ppm; ^13^C NMR (126 MHz, CDCl_3_) δ 154.80, 154.788, 149.35, 149.33, 146.92, 146.86, 112.776, 110.68, 110.665, 85.78, 85.76, 73.39, 73.17, 52.10, 52.01, 46.18, 46.13, 39.76, 39.74, 39.04, 39.01, 27.68, 27.512, 24.98, 24.94, 22.22, 22.07, 21.84, 21.74, 16.63, 16.62 ppm; IR (neat) ν_max_=3079, 29445, 1793(C=O) cm^−1^; HRMS (ESI+, MeOH) *m/z* calcd. (C_16_H_24_NaO_3_), (M+Na)^+^, 287.1618; found: 287.1620.

### Synthesis of BEMC

BEC (264 mg, 1.0 mmol) was dissolved in DCM (10 mL) in a 25 mL round bottom flask and cooled to 0 °C with an ice/water bath. *m*CPBA (77 % w/w, 270 mg, 1.2 mmol) was added and the reaction mixture stirred at 0 °C. After 2 h, the suspension was filtered and the solid washed with hexane and the solvent removed by rotary evaporation. The remaining oil was dissolved in hexane (20 mL) The solution was then washed with an aqueous solution of Na_2_SO_3_ (3×5 mL, 1 m), a saturated aqueous solution of NaHCO_3_ (3×5 mL) and brine. The organic phase was dried over sodium sulfate and the product was obtained after removal of the solvent in vacuo using a rotary evaporator. The product was purified through column chromatography (8 : 2 Hex:EtOAc, *R*_f_=0.29) to give the title compound. Yield: 140 mg (0.50 mmol, 50 %). ^1^H NMR (400 MHz, CDCl_3_) δ 6.08–5.56 (m, 1H), 5.31–4.68 (m, 2H), 4.50–3.69 (m, 4H), 2.20–0.71 (m, 17H) ppm; ^13^C NMR (126 MHz, CDCl3) δ 154.65, 154.62, 149.62, 149.49, 148.23, 148.19, 111.35, 111.33, 110.45, 110.42, 85.55, 85.47, 85.44, 73.62, 73.31, 73.01, 72.69, 58.26, 58.18, 57.66, 56.31, 56.18, 52.98, 52.94, 52.75, 52.62, 50.49, 50.39, 45.60, 45.57, 45.55, 45.34, 40.67, 40.09, 39.65, 39.06, 39.00, 29.70, 24.24, 23.95, 23.77, 23.62, 23.10, 22.87, 22.72, 22.10, 21.93, 21.83, 21.80, 21.73, 21.43, 21.37, 19.70, 19.63, 17.38, 17.32, 17.02, 16.93 ppm; IR (neat) ν_max_ 1791 (C=O) cm^−1^. HRMS (ESI+, MeOH) *m/z* calcd. (C_16_H_24_NaO_4_), (M+Na)^+^, 303.1568; found: 303.1567.

### Synthesis of BEDC

BED (4.0 g, 1.69×10^−2^ mol) and PPNCl (388 mg, 6.77×10^−4^ mol, 2.0 mol % with respect to the epoxide groups) were weighed into a 100 mL Teflon vessel equipped with a magnetic stirring bar and placed into a 150 mL autoclave reactor. The lid was closed, and the system pressurized with CO_2_ (5 bar) and vented three times. Then, the reactor was pressurized at the selected pressure of 40 bar and placed in a heating mantle set at 130 °C. After stirring for 72 h, the reactor was cooled down with an ice bath. Mesitylene (10 mol %) was added as an internal standard, and the reaction analyzed by ^1^H NMR (CDCl_3_) to determine the conversion and selectivity. The reaction mixture was purified by column chromatography (Hex:EtOAc, gradient from 4 : 1 to 2 : 1). Yield: 4.8 g (14.8 mmol, 87 %). ^1^H NMR (400 MHz, CDCl_3_) δ 5.98–5.63 (m, 1H), 5.11–4.83 (m, 2H), 4.40–4.19 (m, 1H), 4.12–4.01 (m, 1H), 2.70–2.31 (m, 2H), 2.01–0.85 (m, 17H) ppm; ^13^C NMR (101 MHz, CDCl_3_) δ 154.64, 154.62, 154.47, 154.44, 154.42, 154.05, 154.03, 154.00, 150.68, 150.66, 149.88, 149.83, 148.62, 148.55, 148.36, 148.23, 111.85, 111.68, 111.08, 110.41, 87.14, 87.02, 86.88, 86.80, 85.66, 85.63, 85.38, 85.32, 85.28, 75.61, 75.50, 73.29, 73.27, 73.15, 73.08, 73.06, 72.90, 72.79, 67.11, 63.66, 53.52, 52.48, 52.45, 52.25, 51.13, 51.08, 46.84, 46.79, 45.84, 45.26, 45.12, 45.07, 45.01, 42.32, 41.83, 41.80, 41.75, 39.39, 39.27, 39.22, 38.67, 38.59, 38.58, 27.88, 27.78, 26.32, 25.95, 23.79, 23.32, 22.99, 22.93, 22.81, 22.62, 21.95, 21.89, 21.81, 21.74, 21.69, 21.61, 21.45, 21.40, 21.38, 21.35, 16.91, 16.82, 16.64, 16.57, 15.88 ppm; IR (neat) ν_max_ 1783 (C=O) cm^−1^; HRMS (ESI+, MeOH) *m/z* calcd. (C_17_H_24_NaO_6_), (M+Na)^+^, 347.1479; found: 347.1465.

### Typical procedure for NIPU synthesis using various diamines and BEDC

In a Schlenk tube, both BEDC and the corresponding diamine were combined in a 1 : 1 ratio. After purging with N_2_, the reaction mixture was heated to 130 °C and stirred for 48 h. Hereafter, the reaction mixture was allowed to reach room temperature, dissolved in MeOH and the NIPU precipitated by addition of hexane. This work up procedure was repeated three times and the precipitated polymer was then dried under vacuum. For the *M*_n_, *Ð*, *T*_g_ and *T*_d_ values of all isolated NIPU oligomers, see Table 2.

### Analysis data for BEDCU6

^1^H NMR (500 MHz, DMSO‐d_6_) δ 6.03–5.59 (m, 1H), 5.11–4.67 (m, 2H), 4.54–3.65 (m, 4H), 3.61–3.14 (m, 19H), 2.01–0.61 (m, 33H) ppm; ^13^C NMR (125 MHz, DMSO‐d_6_) δ 157.57, 156.10, 154.80, 154.79, 154.73, 154.70, 154.43, 154.37, 154.26, 154.22, 154.21, 153.39, 152.92, 152. 84, 152.72, 152.67, 152.11, 152.06, 151.28, 150.96, 150.87, 150.70, 150.21, 150.04, 149.98, 149.66, 149.63, 132.39, 126.79, 110.99, 110.96, 110.56, 110.48, 110.43, 110.36, 109.98, 109.95, 109.45, 109.25, 87.86, 87.85, 87.79, 87.77, 87.59, 87.56, 87.54, 87.53, 87.51, 87.48, 87.41, 86.74, 86.57, 86.53, 86.47, 86.29, 86.26, 86.24, 86.20, 86.14, 86.11, 75.51, 75.29. 75.23, 75.10, 75.04, 74.32, 74.09, 73.63, 73.48, 73.44, 73.41, 73.40, 73.30, 73.25, 73.12, 73.01, 72.89, 72.84, 72.48, 72.33, 72.23, 70.82, 68.13, 68.04, 67.86, 67.78, 66.99, 65.66, 65.63, 63.47, 63.38, 61.38, 60.20, 55.93, 54.82, 54.54, 52.69, 52.32, 52.27, 52.21, 51.81, 51.46, 51.44, 50.92, 50.85, 48.23, 47.76, 47.75, 47.66, 47.58, 47.53, 47.44, 47.08, 46.94, 46.71, 46.67, 46.53, 45.75, 45.66. 45.62, 44.92, 44.91, 44.79, 44.76. 44.66, 44.61, 44.45, 43.76, 43.51, 43.30, 43.12, 43.09, 42.99, 41.98, 41.95, 41.23, 41.05, 40.76, 38.85, 38.83, 37.88, 36.86, 36.73, 36.52, 36.23, 36.10, 36.05, 35.47, 35.33, 35.30, 35.27, 31.98, 31.91, 31.87, 31.78, 31.76, 31.74, 31.70, 30.33, 30.08, 29.75, 28.51, 27.98, 27.89, 27.86, 27.84, 27.82, 27.72, 27.32, 26.66, 25.88, 25.56, 25.34, 25.24, 24.86, 24.69, 24.62, 24.56, 24.47, 24.37, 24.25, 23.64, 23.59, 23.47, 23.37, 23.02, 22.93, 22.81, 22.61, 22.58, 22.47, 22.44, 22.36, 22.25, 22.14, 22.04, 21.98, 21.95, 21.85, 21.80, 21.77, 21.70, 21.64, 21.57, 21.50, 21.47, 21.40, 21.12, 17.53, 17.26, 17.18, 17.05, 16.80, 16.78, 16.76, 16.75, 16.62, 16.54, 16.53, 16.50, 16.48, 16.46, 16.25, 14.55 ppm; IR (neat) ν_max_=3348 (NH and NH2), 2971, 2870, 1796 (carbonate), 1714, 1650, 1537 (NH amide), 1455, 1375, 1258 (C−O urethane), 1089, 1059, 909, 775 cm^−1^.

For the analysis data of the other BEDCUs together with their NMR and IR spectra, see the Supporting Information.

## Conflict of interest

The authors declare no conflict of interest.

1

## Supporting information

As a service to our authors and readers, this journal provides supporting information supplied by the authors. Such materials are peer reviewed and may be re‐organized for online delivery, but are not copy‐edited or typeset. Technical support issues arising from supporting information (other than missing files) should be addressed to the authors.

Supporting Information

## Data Availability

The data that support the findings of this study are available from the corresponding author upon reasonable request.
