# Supplementary material for: Renewable Beta‐Elemene Based Cyclic Carbonates for the Preparation of Oligo(hydroxyurethane)s
Source: ChemSusChem. 2022 Jul 25;15(17):e202201123. doi: 10.1002/cssc.202201123 (PMC9541927; doi:10.1002/cssc.202201123)
Supplement: Supplementary file 1 — Supporting Information [file CSSC-15-0-s001.pdf]

ChemSusChem

Supporting Information

Renewable Beta-Elemene Based Cyclic Carbonates for the Preparation of Oligo(hydroxyurethane)s

Cristina Maquilón[+], Arianna Brandolese[+], Christian Alter, Claas H. Hövelmann, Francesco Della Monica,* and Arjan W. Kleij*This publication is part of a Special Collection highlighting "The Latest Research from our Board Members". Please visit the Special Collection at .© 2022 The Authors. ChemSusChem published by Wiley-VCH GmbH. This is an open access article under the terms of the Creative Commons Attribution License, which permits use, distribution and reproduction in any medium, provided the original work is properly cited.

Table of Contents

Page S2. General Considerations

Page S4. Synthesis of β-elemene oxides

Page S8. Reaction conditions for β-elemene dicarbonate (**BEDC**) formation

Page S9. Halide salts catalysts screening

Page S12. Screening of **BEDC** formation with PPNCl

Page S13. Gram-scale synthesis of **BEDC** under optimized conditions

Page S25. Synthesis of **BEMC** via β-elemene monoxide

Page S35. General procedure for the urethane synthesis

Page S45. General procedure for NIPU synthesis using various diamines and **BEDC**

Page S52. Thermal curing

Page S53. Physical tests

Page S56. NMR Spectra

Page S67. FT-IR spectra

Page S70. Gel permeation chromatography

Page S75. Differential scanning calorimetry (DSC)

Page S78. Thermogravimetric analyses of the polyesters

Page S81. References

S2. General Considerations

All water-sensitive operations were carried out under a nitrogen atmosphere using standard vacuum line and Schlenk techniques. Solvents were purchased from Sigma-Aldrich (HPLC grade) and dried using an MBraun MBSPS800 purification system. NMR spectra were recorded on a Bruker AV-400 spectrometer operating at 400 MHz to collect ^{1}H and 100 MHz for ^{13}C NMR spectral data. ^{1}H NMR spectra are referenced to the residual solvent peak at δ 7.26 ppm for $CDCl_3$, and δ 2.50 ppm for DMSO-d_6. ^{13}C NMR spectra are referenced to the residual solvent peak at δ 77.16 ppm for $CDCl_3$, and δ 39.52 ppm for DMSO-d_6. Differential scanning calorimetry (DSC) analyses for determination of the glass transition temperatures (T_g) were measured under a N_2 atmosphere using a TA-Instruments Discovery DSC2500 equipment. Samples were weighed into aluminum crucibles/cups and subjected to two heating cycles (–80 to 130 ºC) at a heating rate of 10 °C/min. Thermogravimetric analyses (TGA) were recorded under N_2 atmosphere using a TA Instruments Discovery TGA-5500 equipment. Samples were weighed into aluminum crucibles/cups and heated from 0 to 400 ºC at a heating rate of 10 ºC/min. Gel permeation chromatography (GPC) measurements were performed by BASF Kompetenzzentrum Analytik/PolymerLC-Labor using an Agilent HPLC system equipped with a DRI Wyatt Optilab DSP detector. The solvent/eluent used was DMAc + 1% TFAc + 0,5% LiBr at 40 ºC at a flow rate of 1 $mL\cdot min^{-1}$. Samples were analyzed at a concentration of 4 $mg\cdot mL^{-1}$ after filtration through a 0.45 µm pore-size membrane. M_n, M_w, and $Đ$ data were derived from the RI signal by a calibration curve based on several poly(methyl methacrylate)s (PMMAs from Polymer Standards Service, mass ranging from 800 to 2.200.000 g/mol) for the analysis of the polymers.

Reagents

All reagents were purchased from commercial suppliers (Aldrich and Acros) and used as received. The catalyst bis(triphenylphosphine)iminium chloride (PPNCl) is commercially

available and was used after recrystallization from dichloromethane and drying under vacuum for 48 h. β-elemene was kindly provided by Isobionics. Novozym® 435 (Lipase acrylic resin from *Candida antarctica)* was kindly provided by Novozyme. The diamines and formulation reagents (Irgacure® 2100 and Laromer® 9000) were supplied by BASF Coatings GmbH (Münster).

S4. Synthesis of β-elemene oxides

Synthesis of β-elemene dioxide (BED)

The β-elemene dioxide (**BED**) was obtained as following, by modification of previously reported procedure.[1] β-elemene (93%, 10.0 g, 45.5 mmol) was dissolved in DCM (455 mL) in a 1 L flask and cooled at 0 ºC with an ice bath. *m*CPBA (77 % w/w, 23.0 g, 0.10 mol) was added during 20 minutes and the reaction stirred at 0 ºC. After 4.3 hours from the first addition, the suspension was filtered, the solid washed with hexane and the solvent removed by rotary evaporation. The resulting mixture was suspended in hexane, filtered and dried again until obtaining a colorless oil. The oil was dissolved in hexane (250 mL) and washed with sat. $NaHCO_3$ (250 mL, 3 times). The organic phase was dried over Na_2SO_3, filtered and the solvent removed by rotary evaporation. The mixture was purified by column chromatography on silica gel (eluent from Hex/EtOAc = 8/2 to Hex/EtOAc = 6/4). In Hex/EtOAc = 8/2, R_f(monoxide) = 0.76; R_f(dioxide) = 0.48-0.57. Yield = 8.1 g (75.3%).

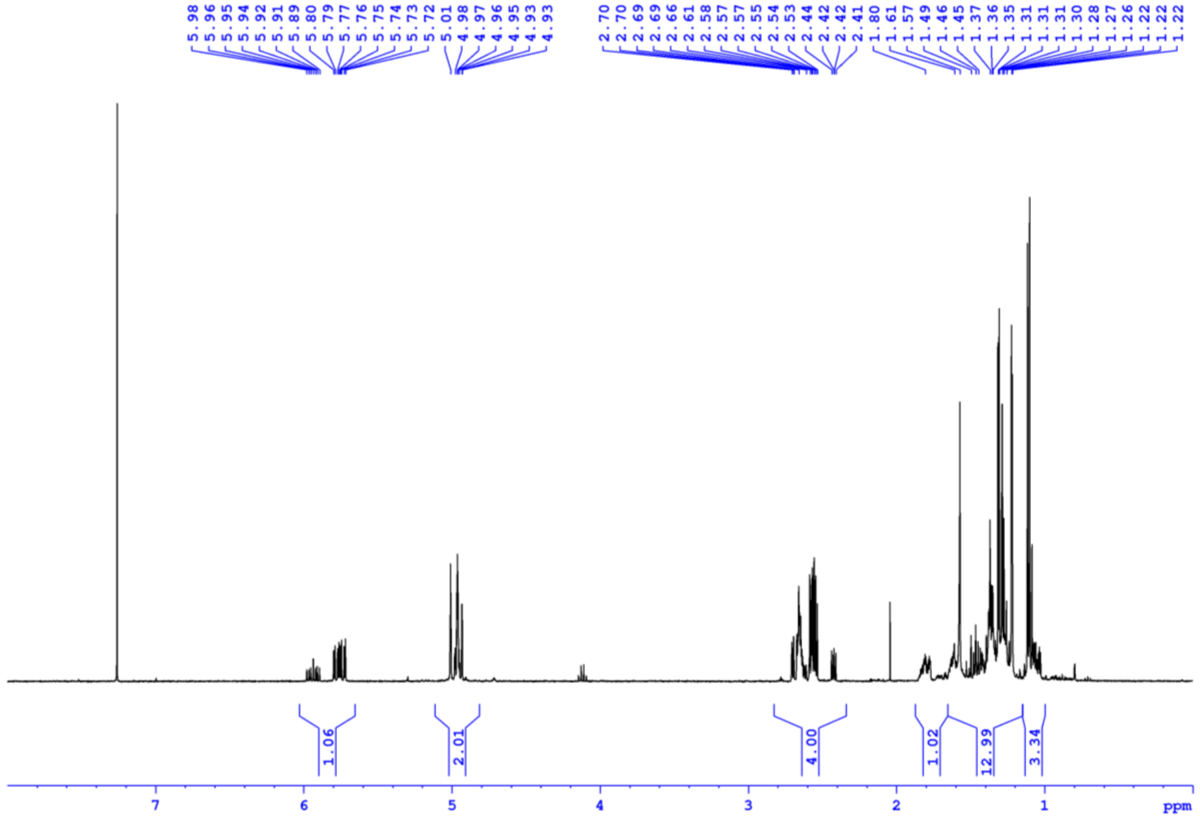

Figure S1. ^{1}H NMR of β-elemene dioxide **BED** (400 MHz, rt, $CDCl_3$).

Synthesis of β-elemene monoxide (BEM) with supported Novozym® 435

A 25 ml round bottom flask was fed with β-elemene (204 mg, 1 mmol), ethyl acetate (5 ml) and Novozym® 435 (0.10 g). Hence H_2O_2 (30%, 100 volume, 1.5 mmol) was slowly added and the flask was gently shaken (100 rpm) at 50 ºC for 4 hours. Upon completion of the reaction, as indicated by TLC, the reaction mixture was filtered through a plug of cotton wool and the organic solvent evaporated prior to dissolution of the residue in DCM (20 ml). The organic layer was then dried using $MgSO_4$, filtered and concentrated in vacuo to afford the target epoxide. The mixture was further purified by column chromatography giving **BEM** (198 mg, 90%) with spectroscopic data in accordance with the literature.[1]

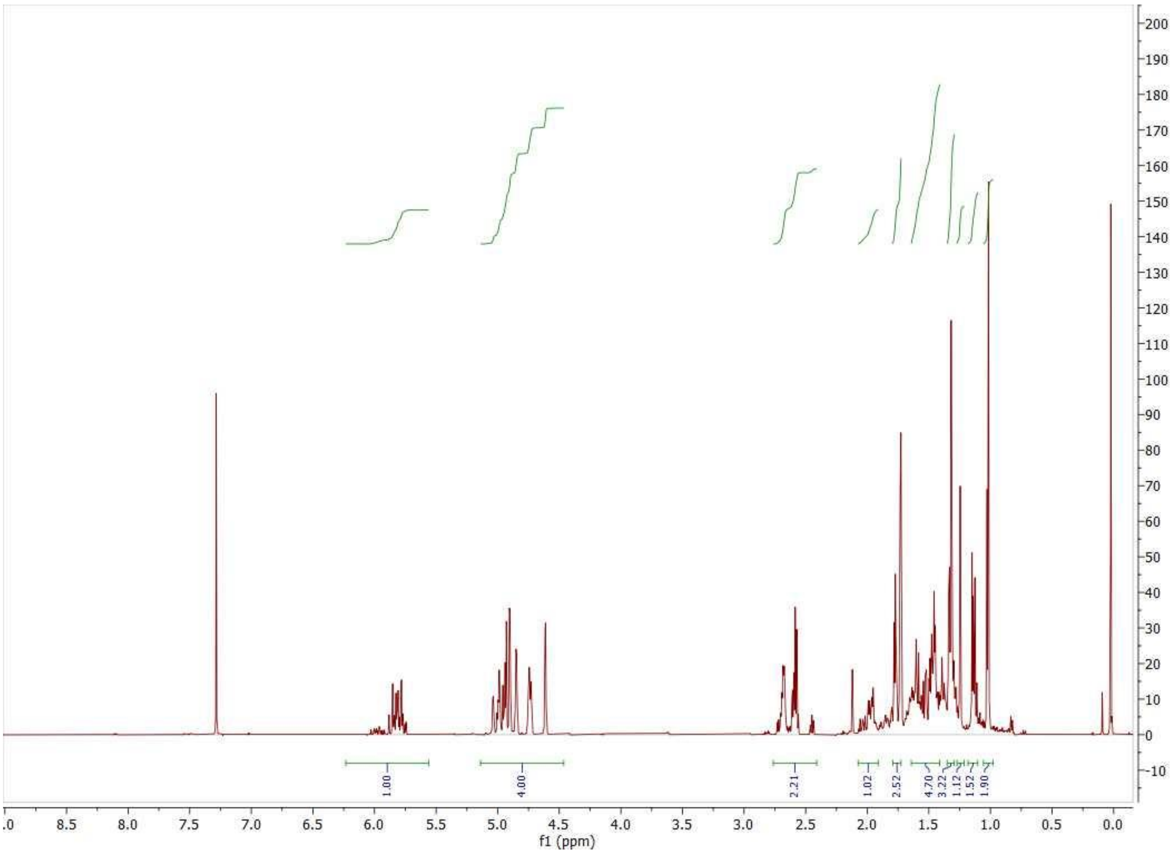

Figure S2. ^{1}H NMR of β-elemene monoxide **BEM** (400 MHz, rt, $CDCl_3$) synthetized with Novozym® 435.

Synthesis of β-elemene dioxide (BED) with supported Novozym® 435

A 25 ml round bottom flask was fed with β-elemene (204 mg, 1 mmol), ethyl acetate (5 ml) and Novozym® 435 (0.20 g). Hence H_2O_2 (30%, 100 volume, 3.0 mmol) was slowly added and the flask was gently shaken (100 rpm) at 50 ºC for 6 hours. Upon completion of the reaction, as indicated by TLC, the reaction mixture was filtered through a plug of cotton wool and the organic solvent evaporated prior to dissolution of the residue in DCM (20 ml). The organic layer was then dried using $MgSO_4$, filtered and concentrated in vacuo to afford the target epoxide. The mixture was further purified by column chromatography giving **BED** (200 mg, 85%) with spectroscopic data in accordance with the literature.[1]

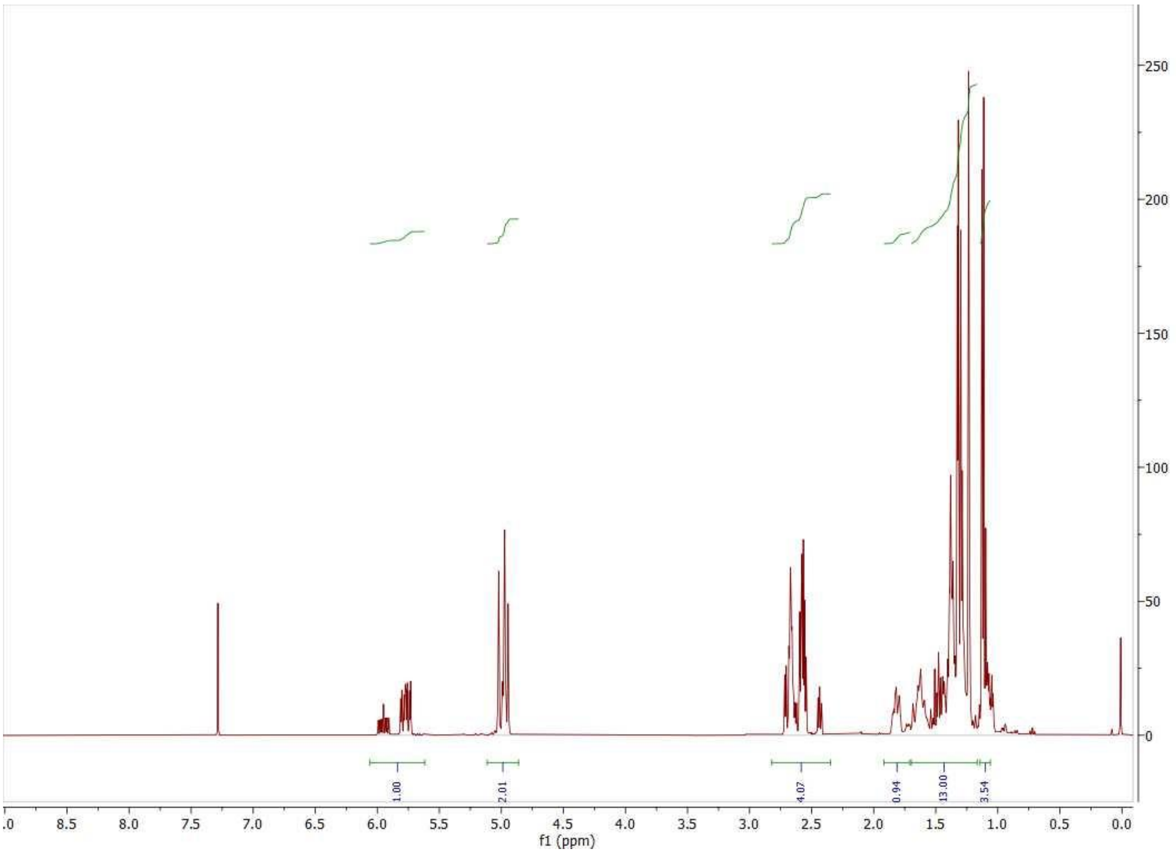

Figure S3. ^{1}H NMR of β-elemene dioxide **BED** (400 MHz, rt, $CDCl_3$) synthetized with Novozym® 435.

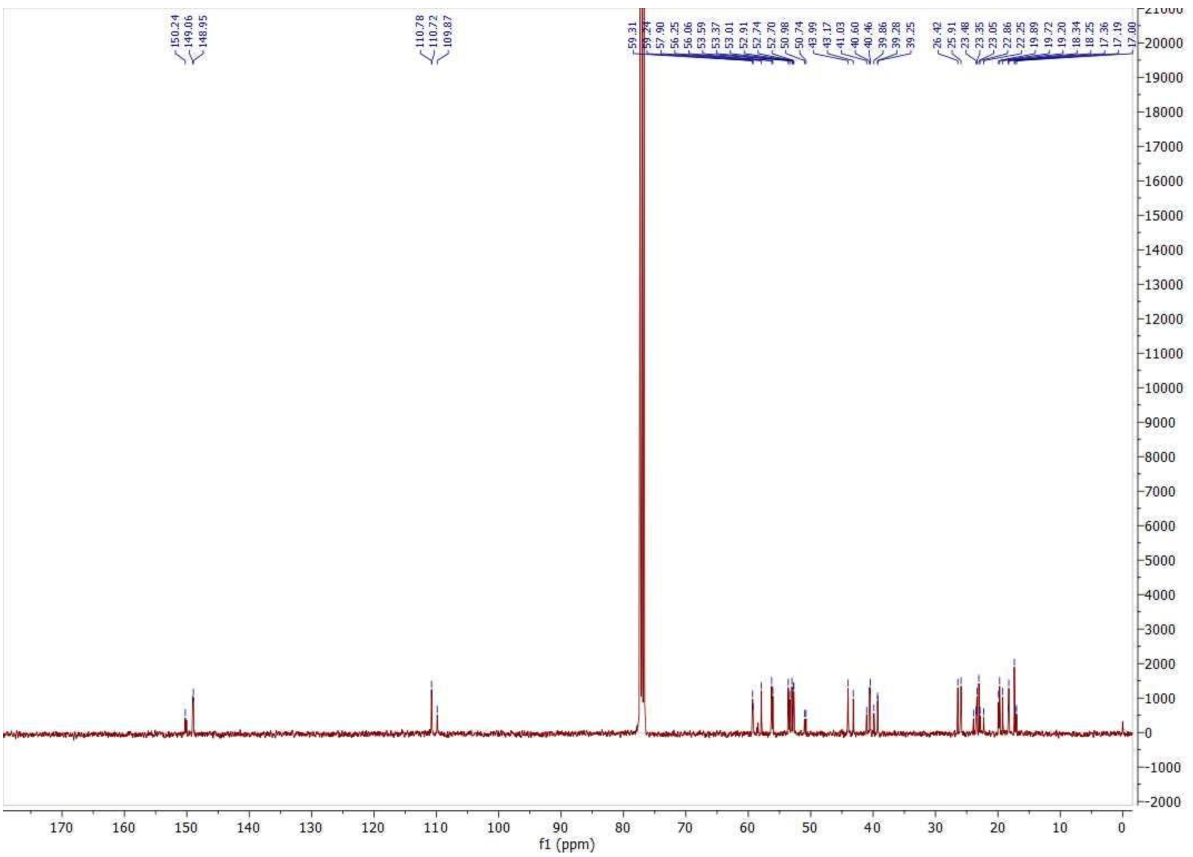

Figure S4. ^{13}C NMR of β-elemene dioxide **BED** (101 MHz, rt, $CDCl_3$) synthetized with Novozym® 435.

S8. Reaction conditions for β-elemene dicarbonate (BEDC) formation

Reaction of **BED** with CO_2 for the synthesis of **BEDC** led to the formation of a mixture of diastereoisomers, due to the composition of the starting bis-epoxide. The formation of **BEDC** proceeds through **BEMC** intermediates and, in this case, eight isomers are virtually possible. Nevertheless, NMR characterization of isolated **BEMC** samples supports the formation of a simpler mixture, likely due to the favorite reaction of the less encumbered epoxide moiety. This results in the selective formation of one major regio-isomer in the form of four different diastereoisomers (**Figure S2**). This scenario is supported by different reactivity observed for double bonds in 3,4 and 11,12 positions of β-elemene during epoxidation with *m*CPBA.[1]

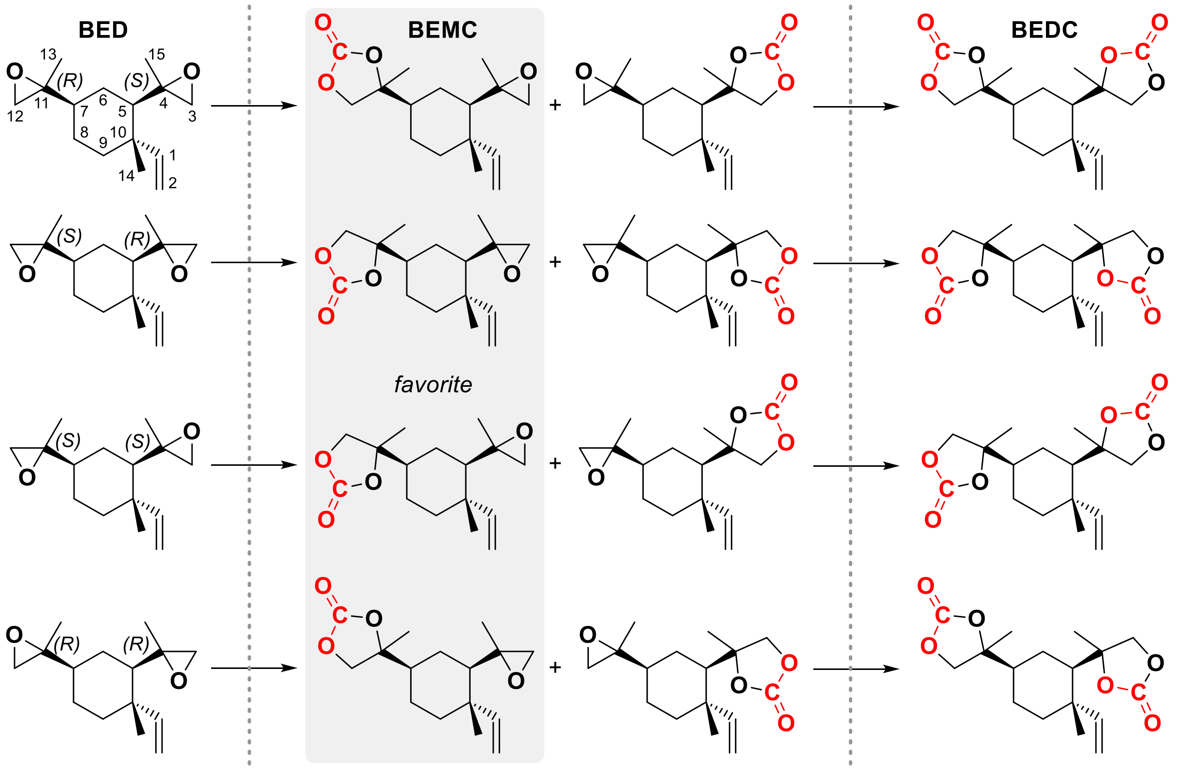

Figure S5. Possible regio- and stereo-isomers for **BEMC** and **BEDC** obtained from **BED**. Proposed favorite **BEMC** regioisomers highlighted with gray background.

S9. Halide salt screening

Screening of halide salts involving TBAC, TBAB and PPNCl was conducted in parallel under the same conditions, using a HEL CAT24 multireactor, according to the following procedure. The **BED** (100 mg, $4.23 \cdot 10^{-4}$ mol) and the selected halide salt catalyst ($1.27 \cdot 10^{-5}$ mol, 1.5 mol% with respect to epoxide groups) are weighted in a glass vessel equipped with a magnetic stirring bar. The vessels with different catalysts are placed into the reactor. The lid was closed, and the system pressurized with CO_2 (5 bar) and vented three times. Then, the reactor was pressurized at the selected pressure of 40 bar, and placed in a heating mantel at 100 °C. After stirring for 24 h, the reactor was cooled down with an ice bath. Mesitylene (5.9 µL, 10 mol%) was added as internal standard, and each reaction analyzed by ^{1}H NMR for conversion and selectivity determination. The reaction mixtures were purified by column chromatography (Hex/EtOAc = 6/4).

The β-elemene monocarbonate (**BEMC**) product was also isolated and fully characterized.

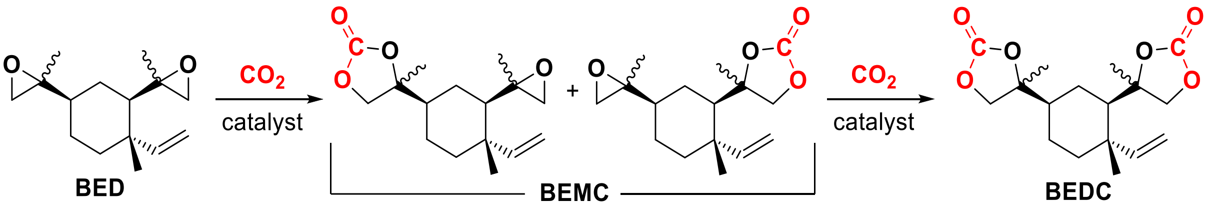

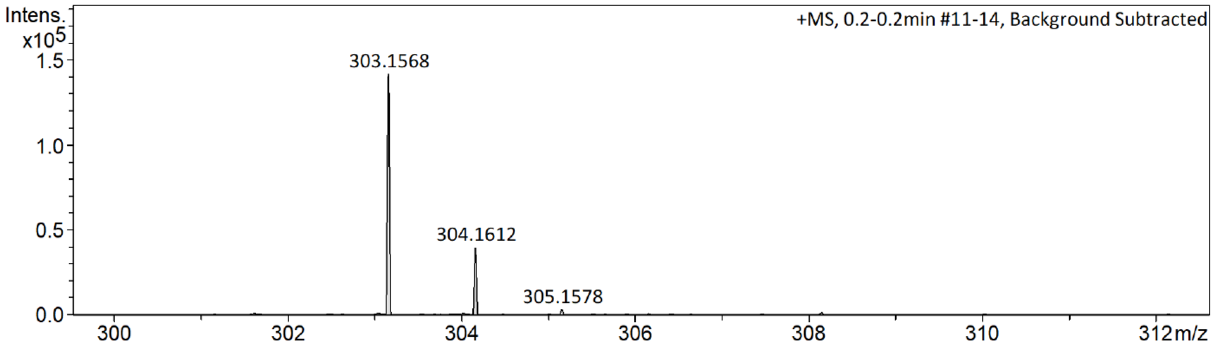

Figure S6. HRMS spectrum of β-elemene monocarbonate **BEMC** $[C_{16}H_{24}O_4Na]^+$ calculated = 303.1567, measured = 303.1568.

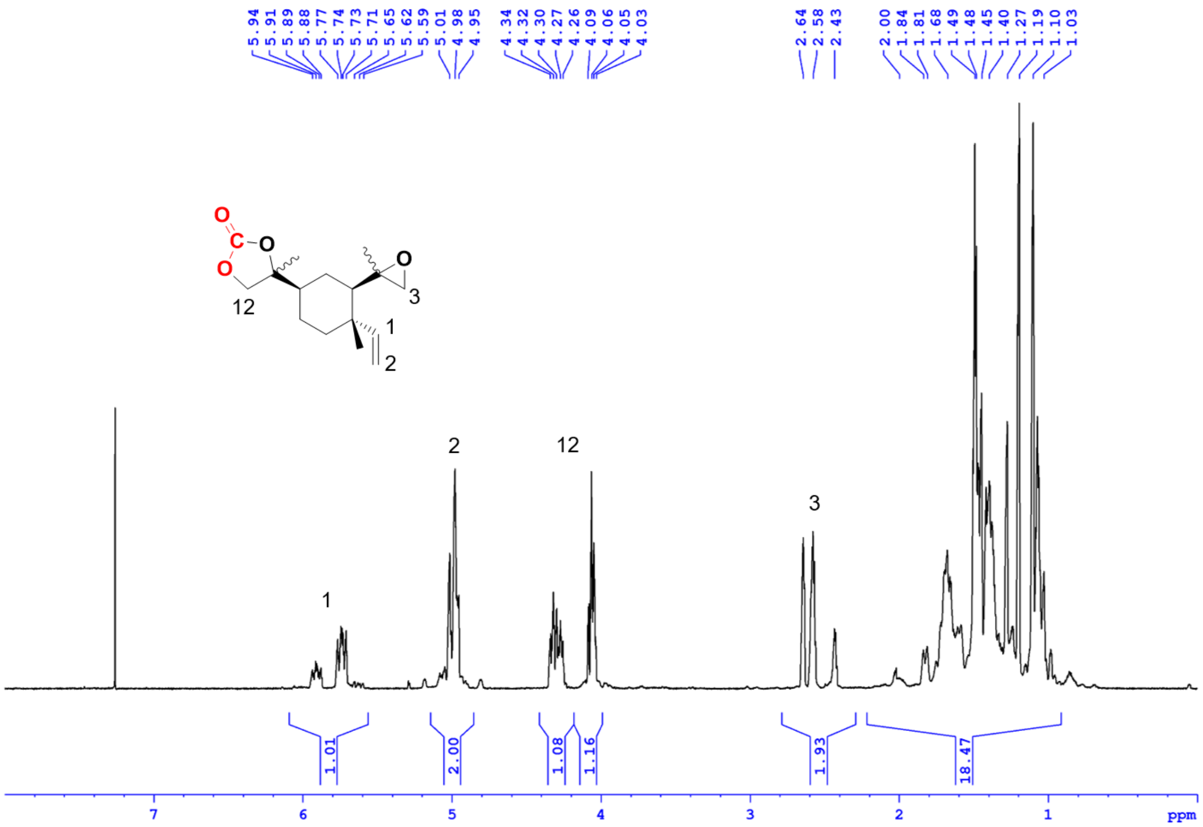

Figure S7. ^{1}H NMR of β-elemene monocarbonate **BEMC** (400 MHz, rt, $CDCl_3$).

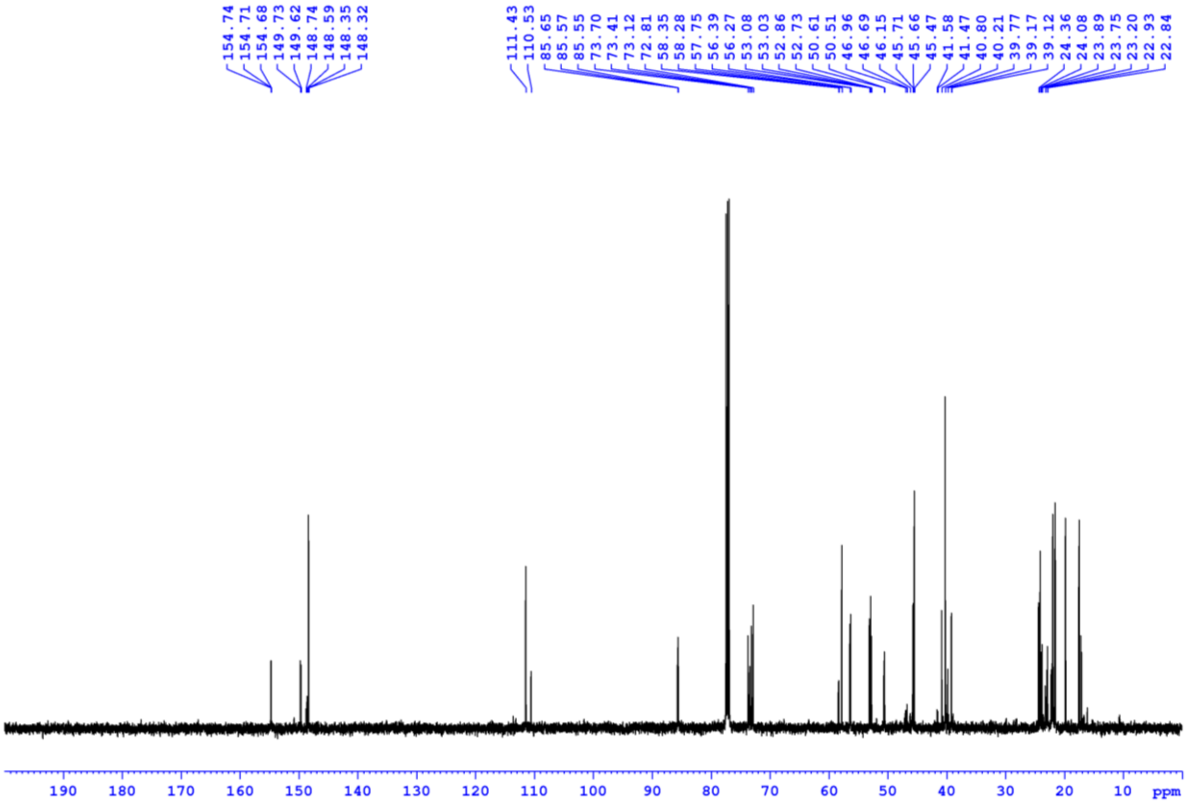

Figure S8. ^{13}C NMR of β-elemene monocarbonate **BEMC** (400 MHz, rt, $CDCl_3$).

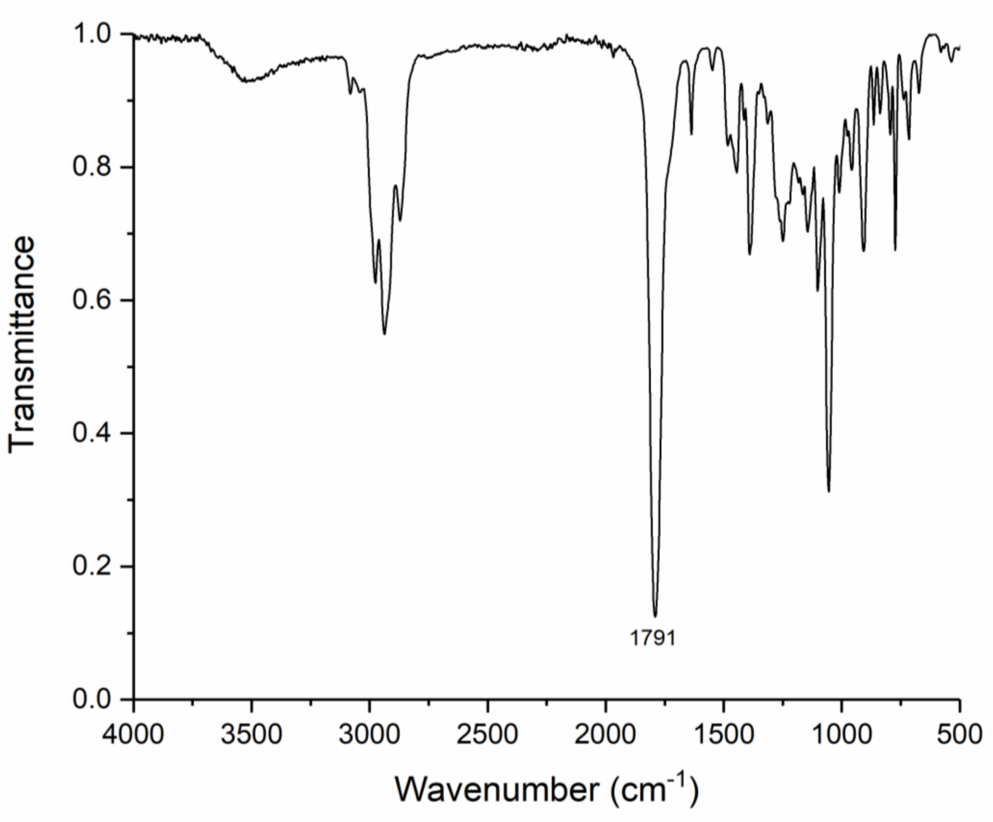

Figure S9. FT-IR of β-elemene monocarbonate **BEMC**.

S12. Screening of BEDC formation with PPNCl

Screening of reaction conditions for the formation of **BEDC** in the presence of PPNCl was conducted using a 40 mL 316SS Berghof reactor, according to the following procedure.

The **BED** (0.1 – 1.0 g, $4.23 \cdot 10^{-4}$ – $4.23 \cdot 10^{-3}$ mol) and PPNCl (1.5 – 2.0 mol% with respect to epoxide groups) are weighted in a Teflon vessel equipped with a magnetic stirring bar and placed into the reactor. The lid is closed, and the system pressurized with CO_2 (5 bar) and vented three times. Then, the reactor is pressurized at the selected pressure of 40 bar, and placed in a heating mantel at 100 – 130 ºC. After stirring for 48-72 h, the reactor is cooled down with an ice bath. Mesitylene (10 mol%) is added as internal standard, and the reaction analyzed by ^{1}H NMR for conversion and selectivity determination.

S13. Gram-scale synthesis of BEDC under optimized conditions

The **BED** (4.0 g, $1.69 \cdot 10^{-2}$ mol) and PPNCl (388 mg, $6.77 \cdot 10^{-4}$ mol, 2.0 mol% with respect to epoxide groups) are weighted in a 100 mL Teflon vessel equipped with a magnetic stirring bar and placed into a 150 mL 316SS Berghof reactor. The lid is closed, and the system pressurized with CO_2 (5 bar) and vented three times. Then, the reactor is pressurized at the selected pressure of 40 bar, and placed in a heating mantel at 130 ºC. After stirring for 72 h, the reactor is cooled down with an ice bath. Mesitylene (10 mol%) is added as internal standard, and the reaction analyzed by ^{1}H NMR for conversion and selectivity determination. The reaction mixture was purified by column chromatography (Hex/EtOAc = 6/4). Yield = 4.79 g (87.3%).

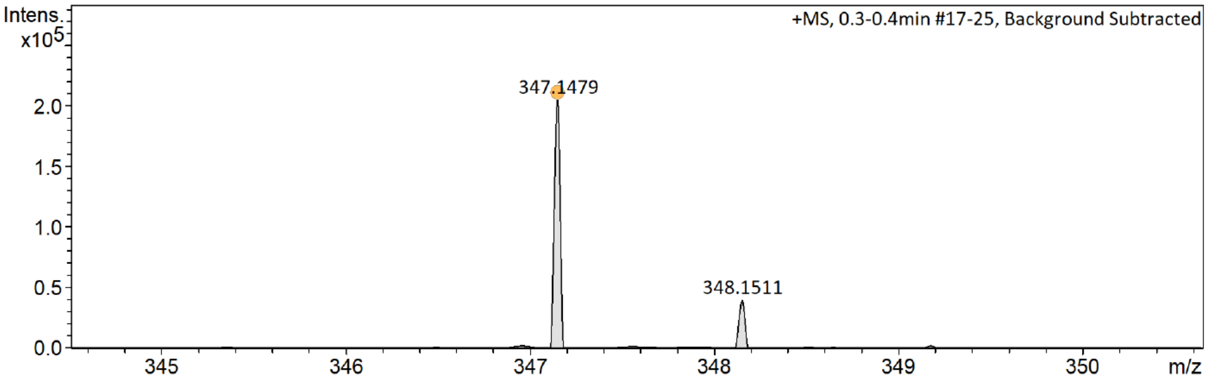

Figure S10. HRMS spectrum of β-elemene dicarbonate **BEDC** $[C_{17}H_{24}O_6Na]^+$ calculated = 347.1465, measured = 347.1479.

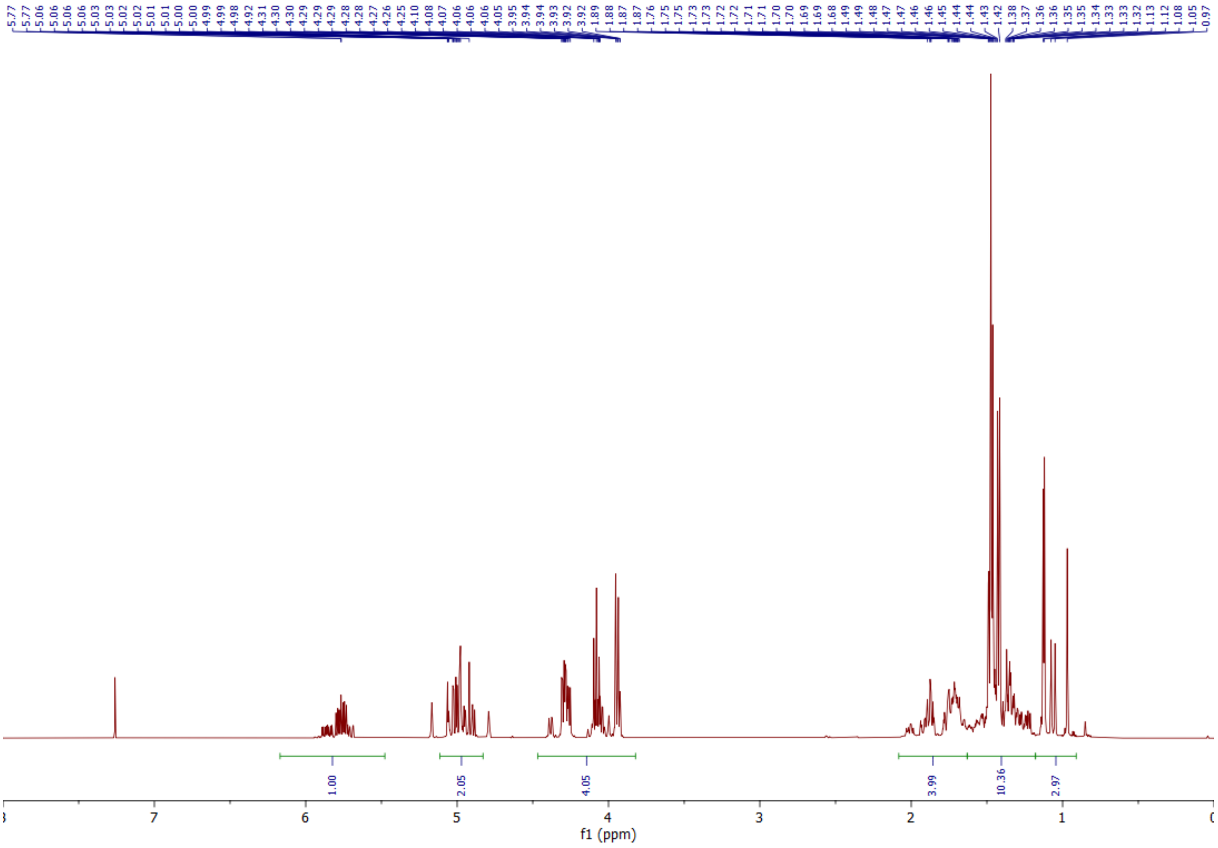

Figure S11. ^{1}H NMR of β-elemene dicarbonate **BEDC** (500 MHz, rt, $CDCl_3$).

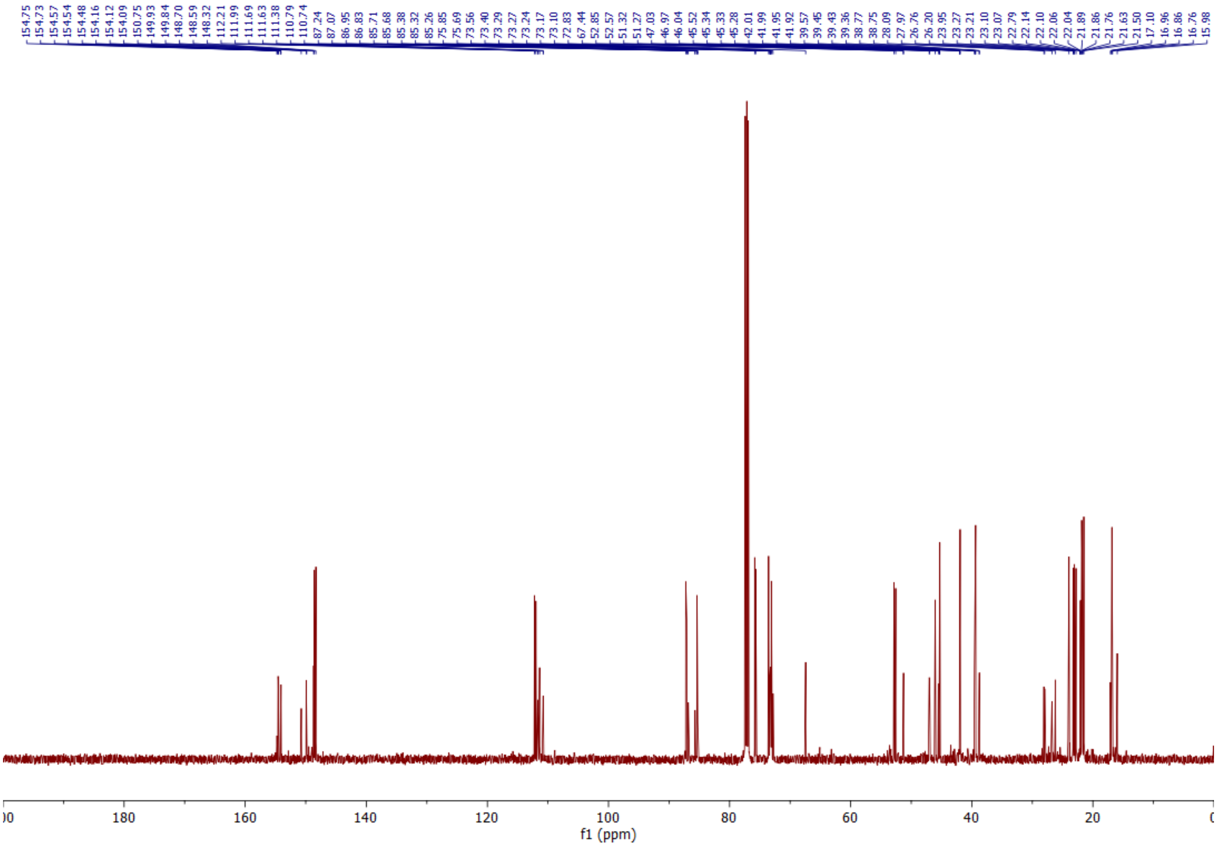

Figure S12. ^{13}C NMR of β-elemene dicarbonate **BEDC** (500 MHz, rt, $CDCl_3$).

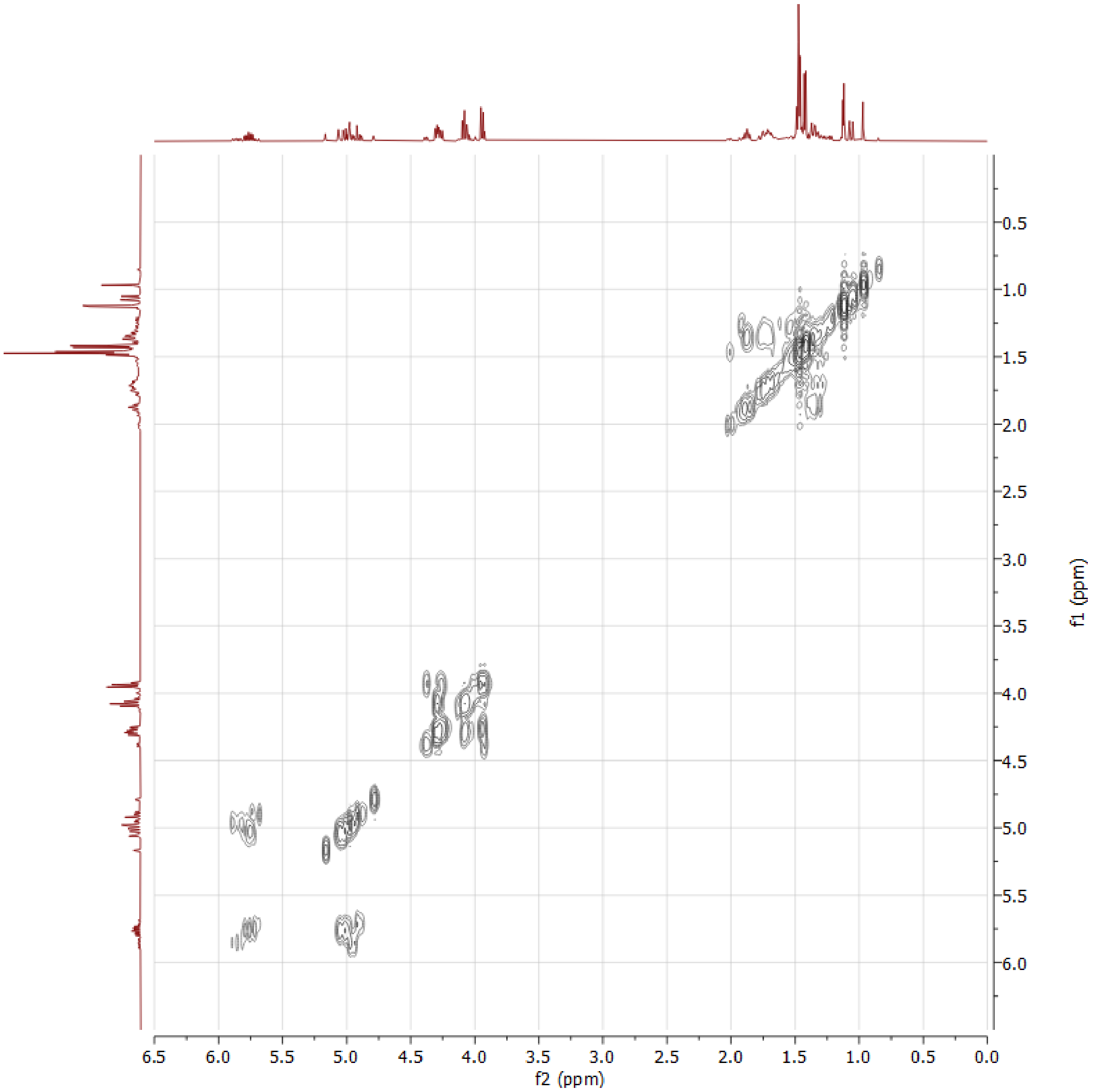

Figure S13. ^{1}H-^{1}H COSY NMR of β-elemene dicarbonate **BEDC** (500 MHz, rt, $CDCl_3$).

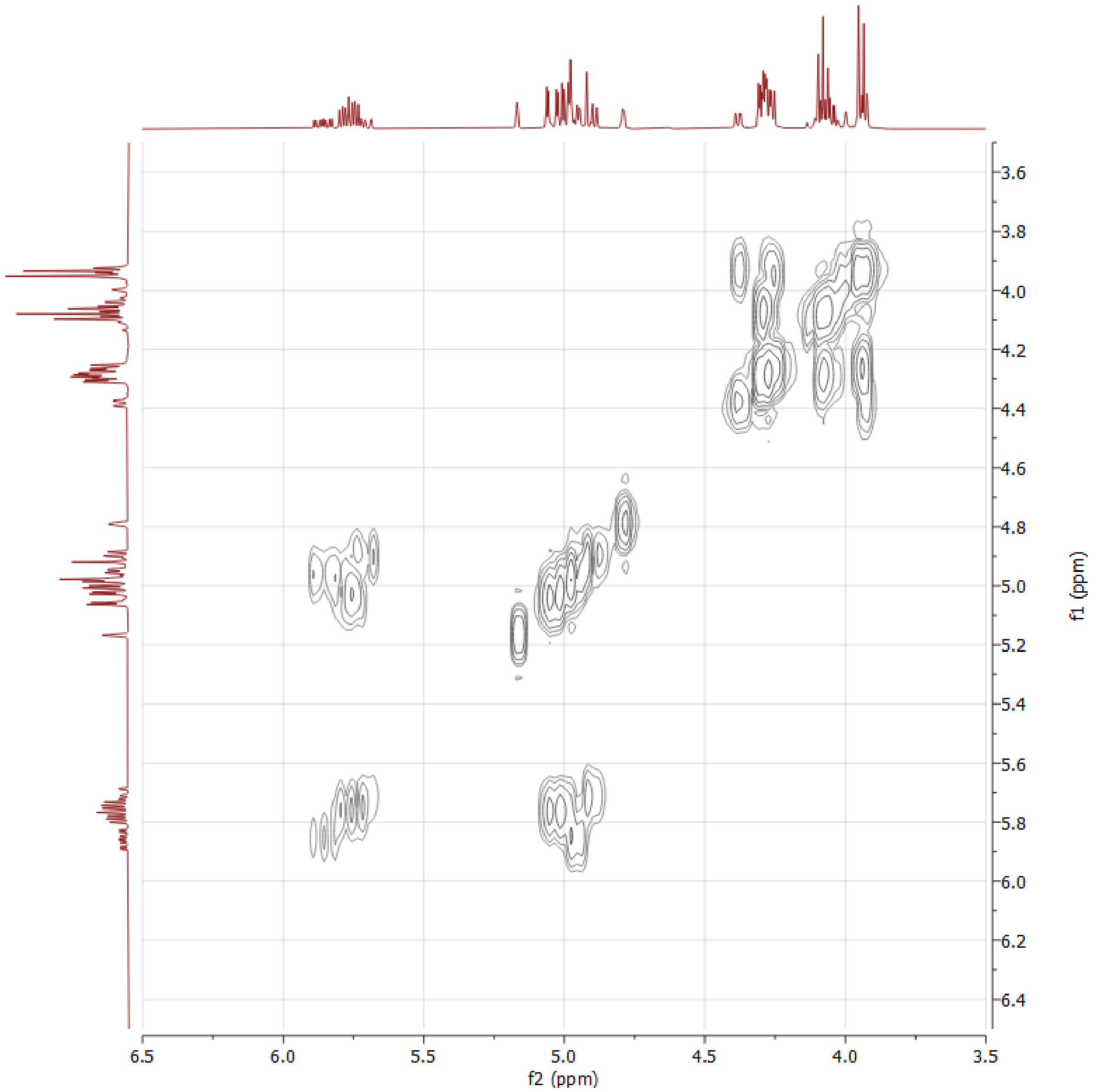

Figure S14. ^{1}H-^{1}H COSY NMR of β-elemene dicarbonate **BEDC** from 6.5 to 3.5 ppm (500 MHz, rt, $CDCl_3$).

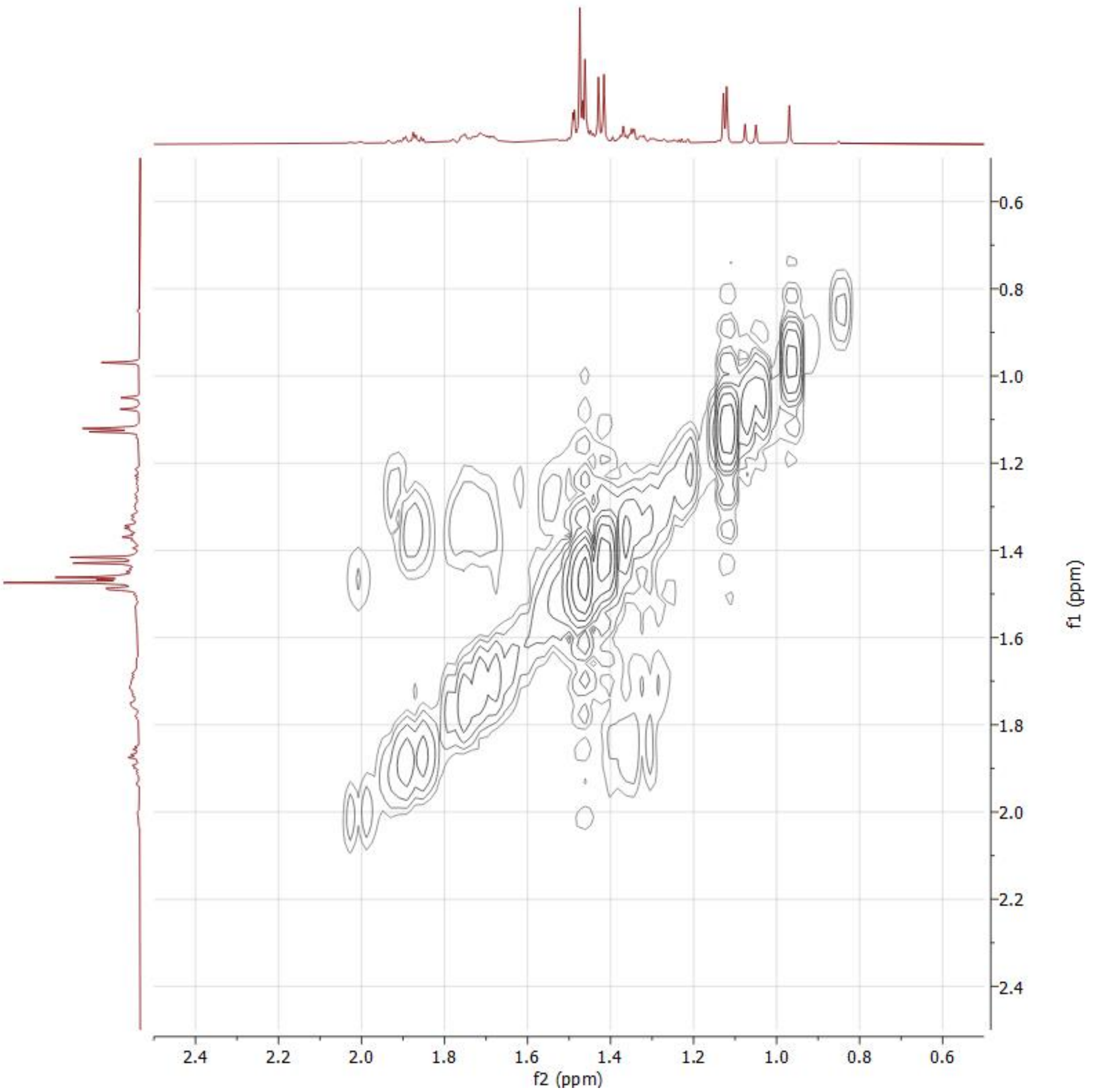

Figure S15. ^{1}H-^{1}H COSY NMR of β-elemene dicarbonate **BEDC** from 2.5 to 0.5 ppm (500 MHz, rt, $CDCl_3$).

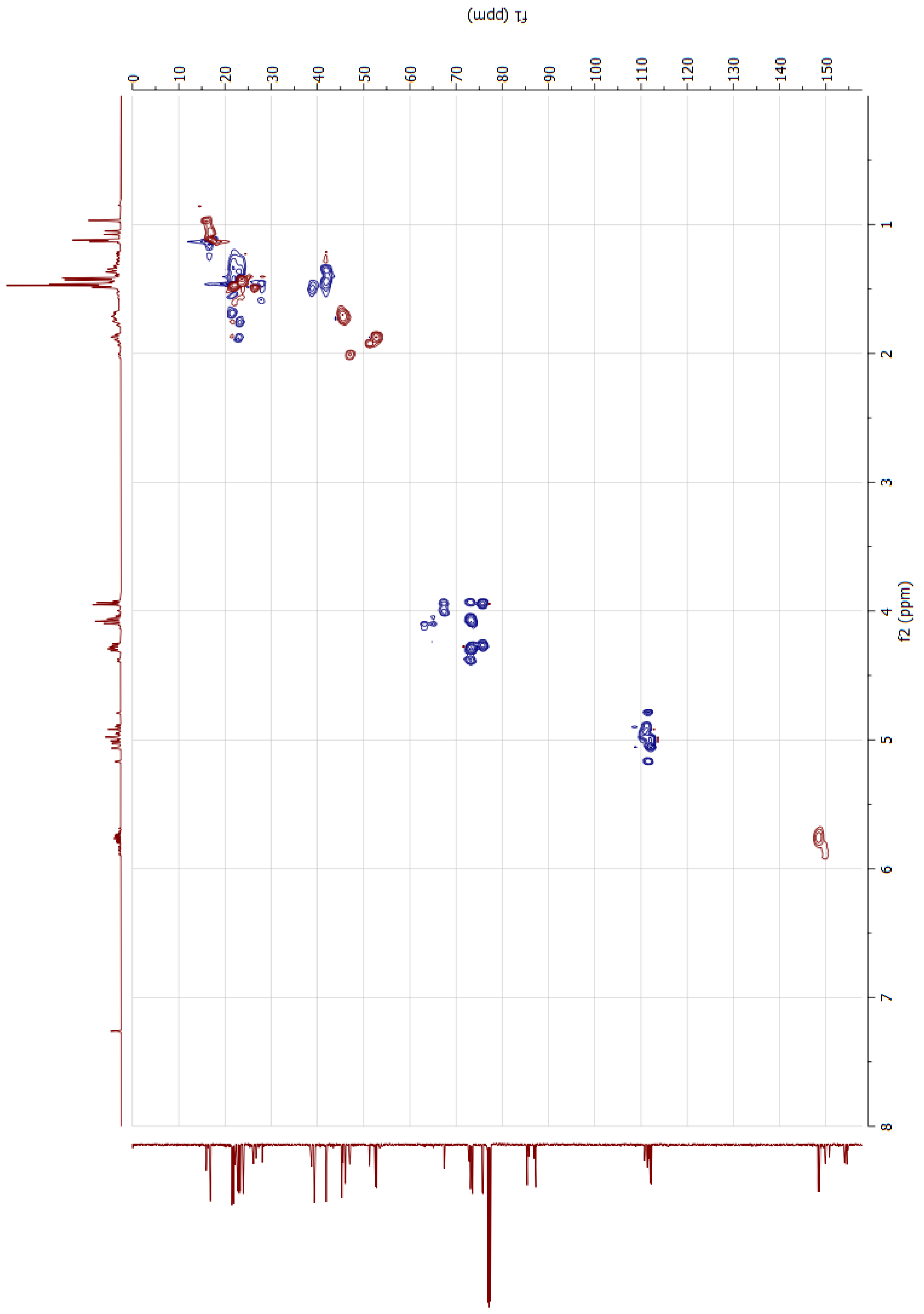

Figure S16. ^{1}H-^{13}C HSQC NMR of β-elemene dicarbonate **BEDC** (400 MHz, rt, $CDCl_3$).

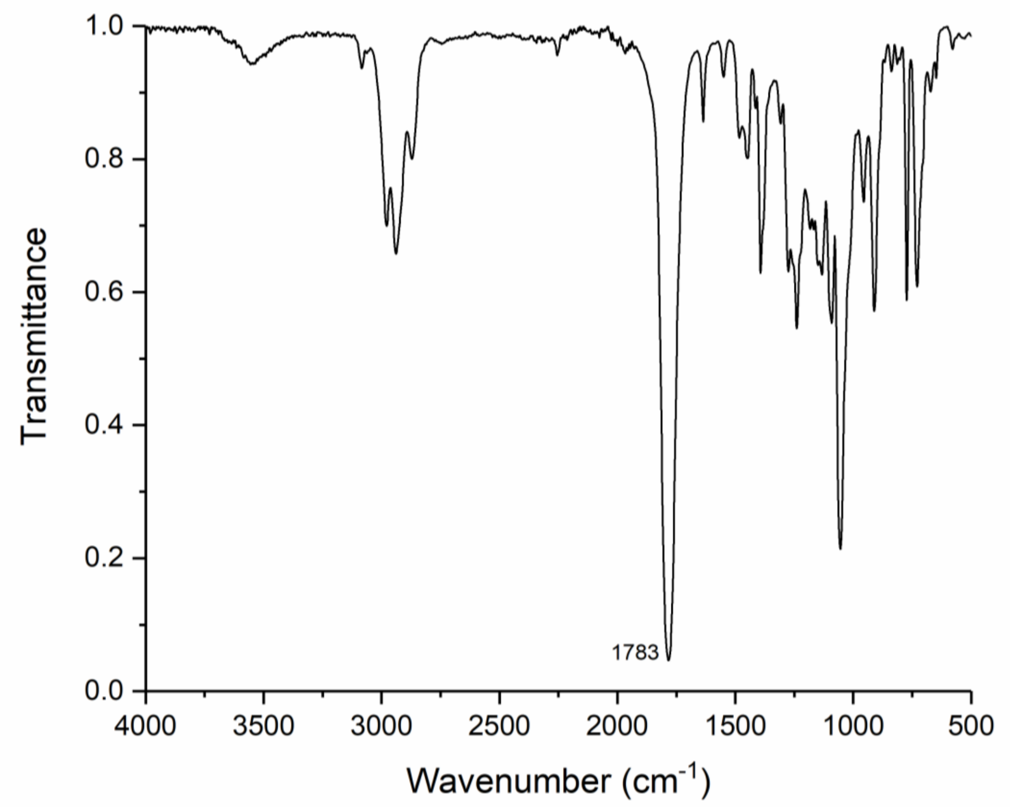

Figure S17. FT-IR of β-elemene dicarbonate **BEDC**.

Purification of BEDC through precipitation

A **BEDC** mixture of diastereoisomers (1.0 g) collected after column chromatography purification was transferred into a 10 mL glass vial, dissolved in EtOAc (1.0 mL), and Et_2O (2.0 mL) was layered on top of the solution. The vial was closed and kept at -20 ºC in the freezer until a white precipitate had formed. The solid was recovered by filtration, washed with cool diethyl ether, and dried in vacuum. Recovered mass: 0.16 g. The diastereoisomer isolated with this procedure was fully characterized via NMR as shown below.

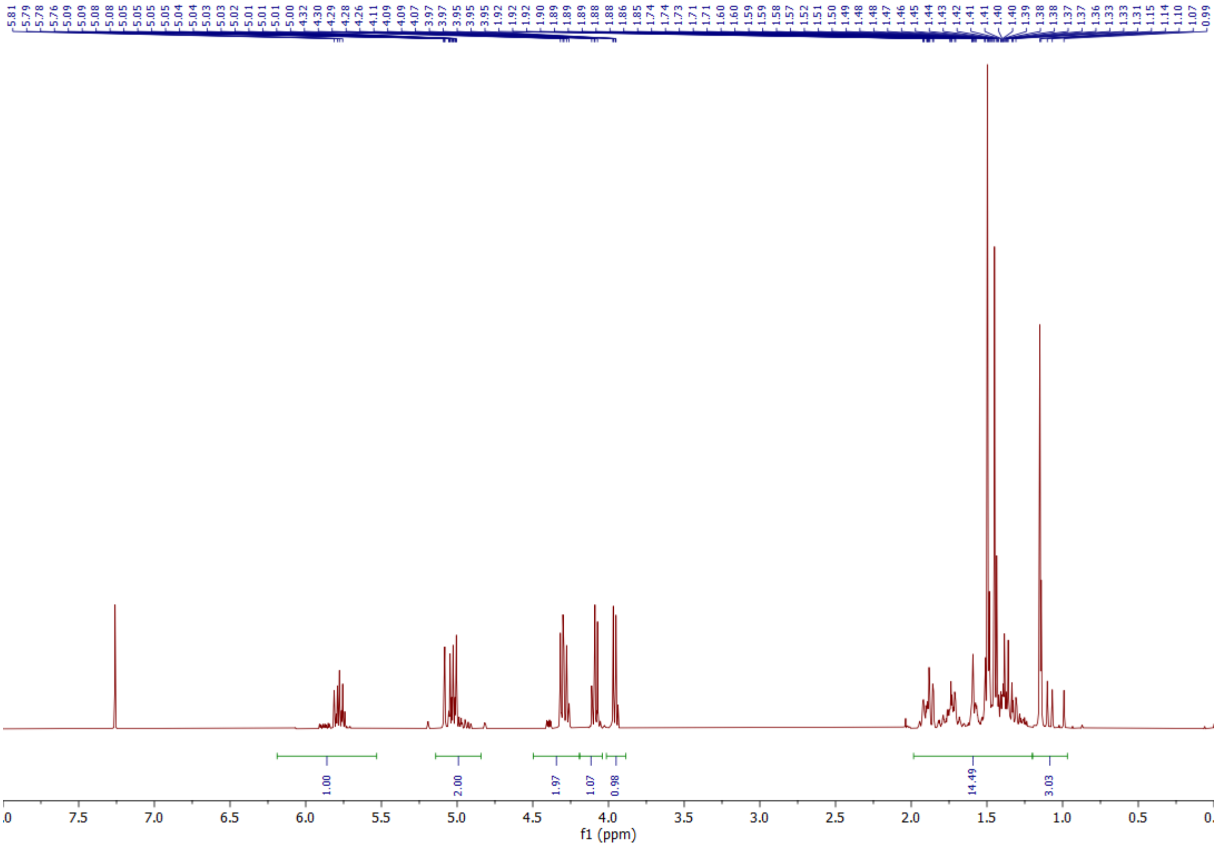

Figure S18. ^{1}H NMR of precipitated **BEDC** (500 MHz, rt, $CDCl_3$).

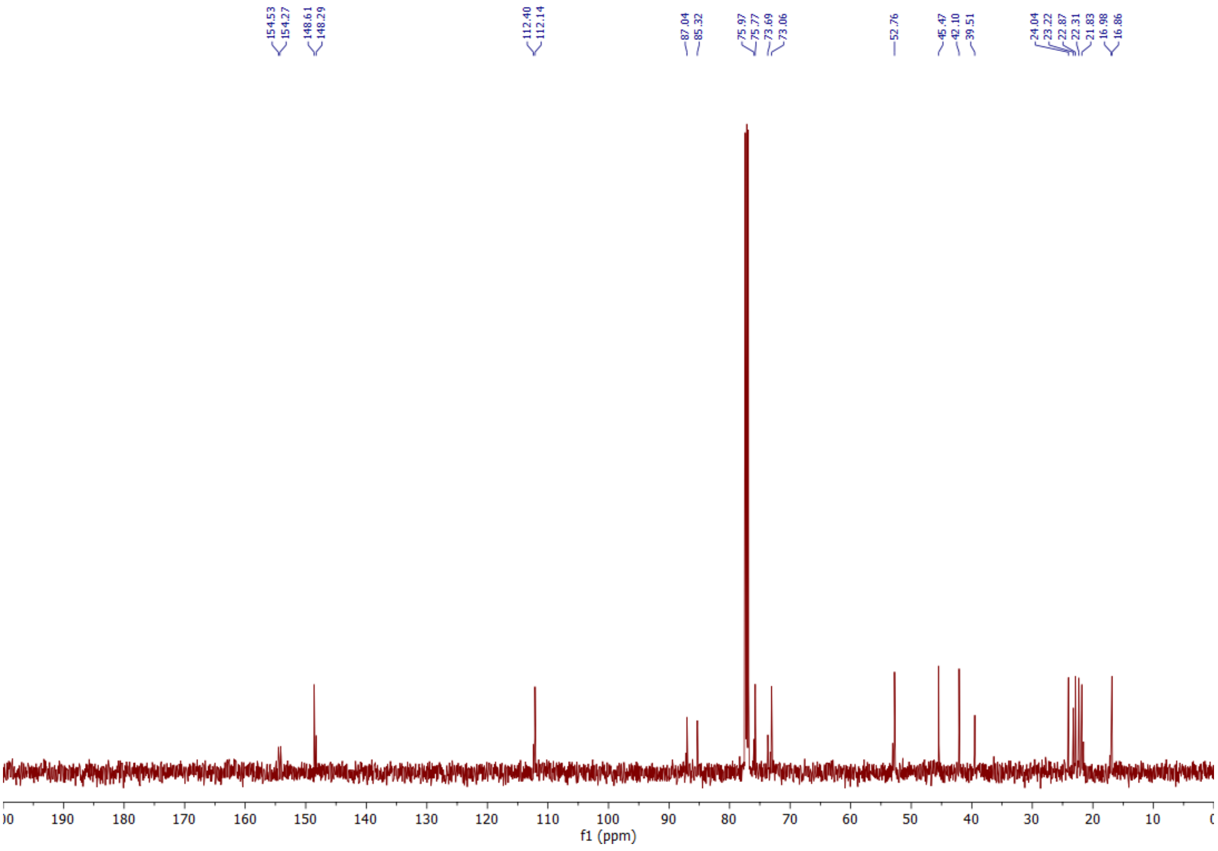

Figure S19. ^{1}H NMR of precipitated **BEDC** (500 MHz, rt, $CDCl_3$).

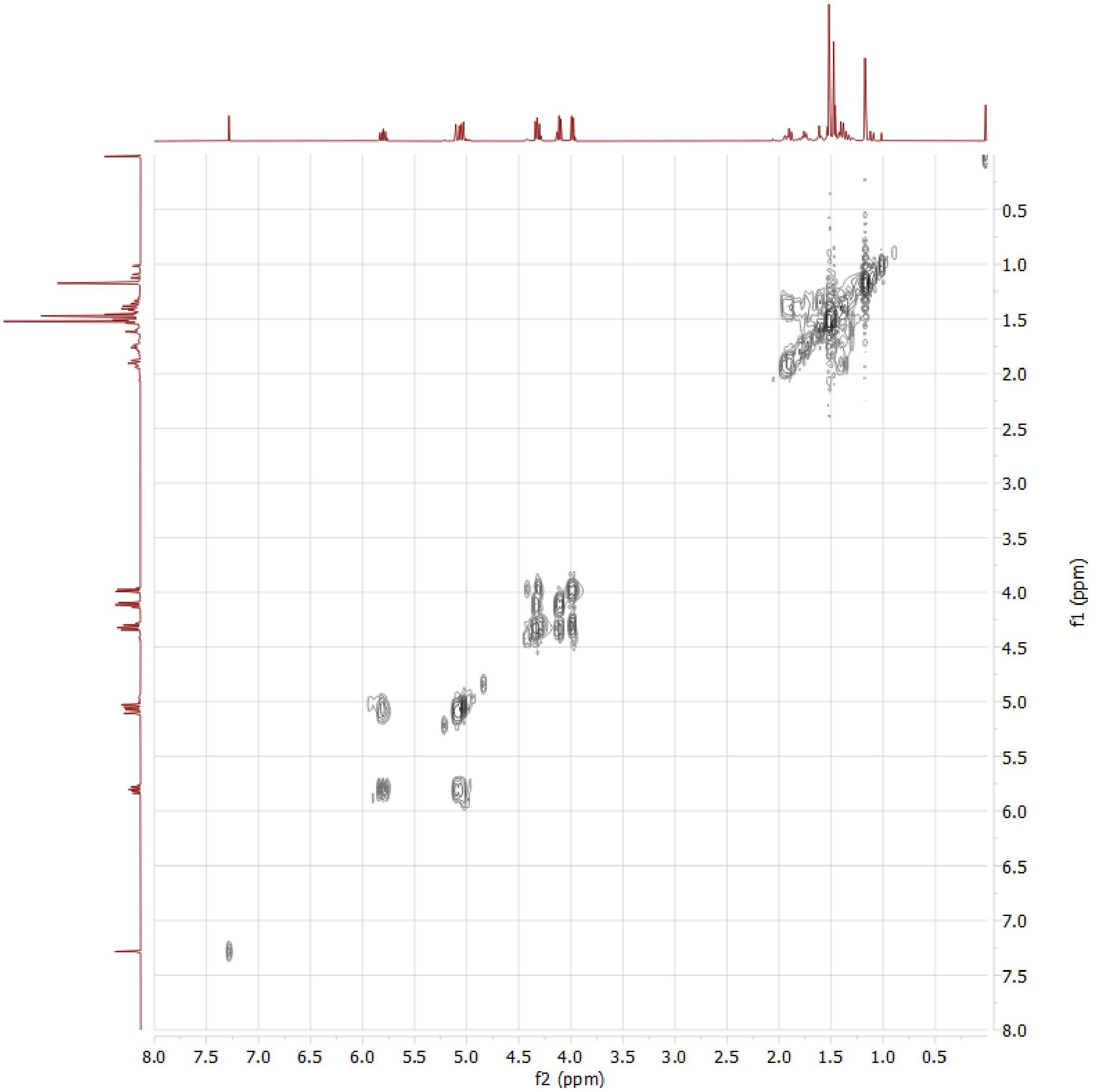

Figure S20. $^{1}H^{1}H$-COSY NMR of precipitated **BEDC** (500 MHz, rt, $CDCl_3$).

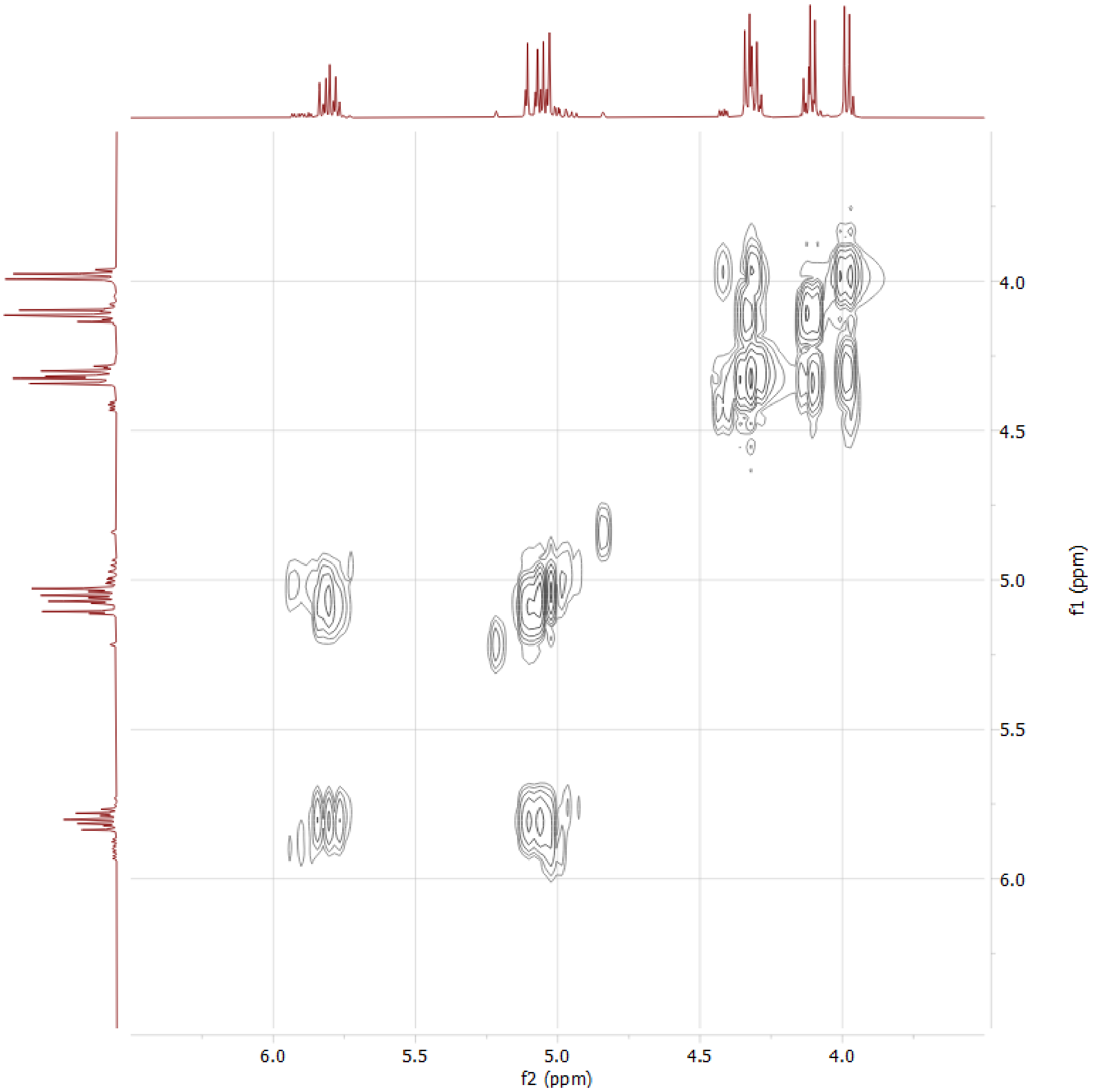

Figure S21. $^{1}H^{1}H$-COSY NMR of precipitated **BEDC** from 6.5 to 3.5 ppm (500 MHz, rt, $CDCl_3$).

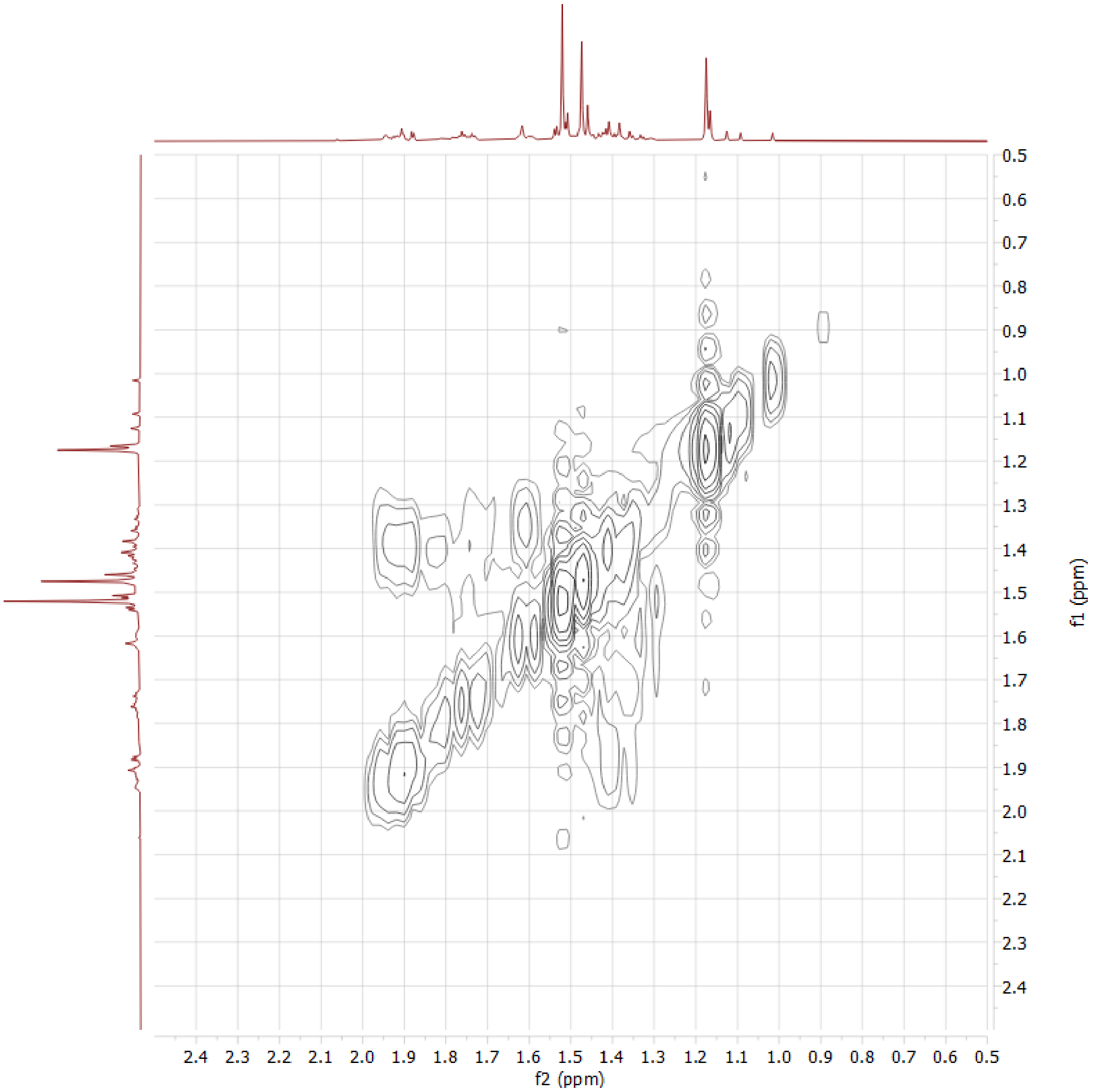

Figure S22. $^1H^1H$-COSY NMR of precipitated **BEDC** from 2.5 to 0.5 ppm (500 MHz, rt, $CDCl_3$).

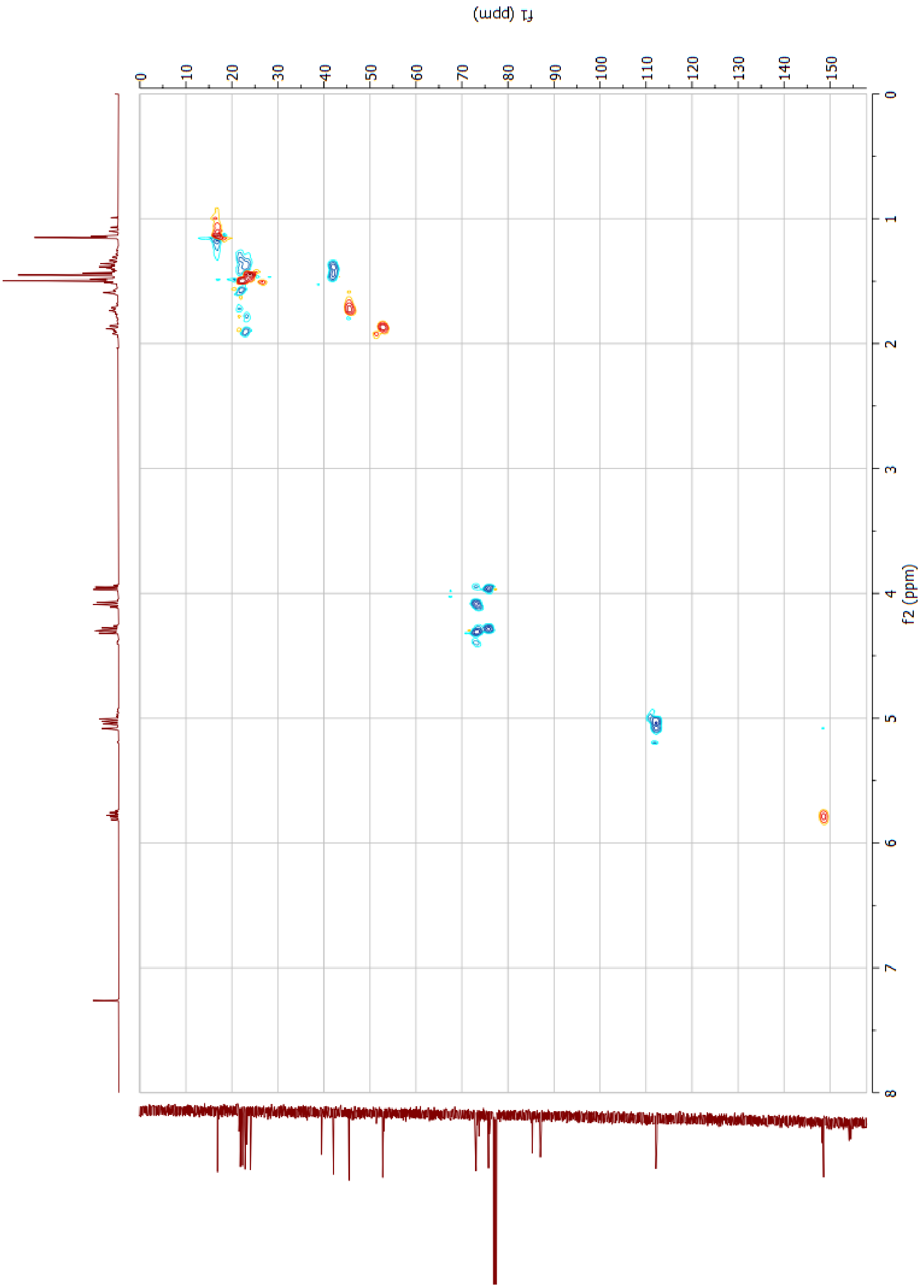

Figure S23. $^{1}H^{13}C$-HSQC NMR of precipitated **BEDC** (500 MHz, rt, $CDCl_3$).

S25. Synthesis of BEMC *via* β-elemene monoxide

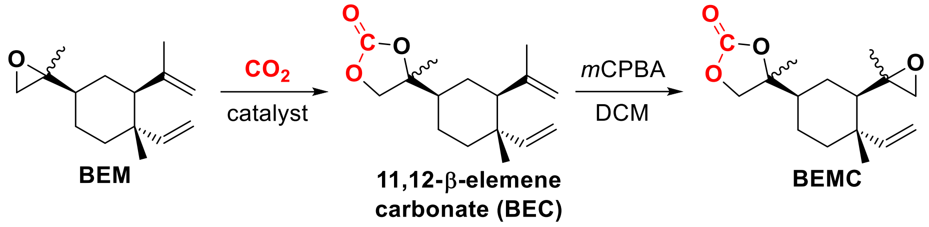

Figure S24. Preparation of **BEMC** through **BEC**.

4-methyl-4-(4-methyl-3-(prop-1-en-2-yl)-4-vinylcyclohexyl)-1,3-dioxolan-2-one (BEC)

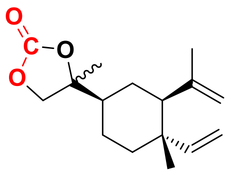

BEM (220 mg, 1.0 mmol) and PPNCl (13 mg, 0.02 mmol, 2.0 mol%) were weighed into a 10 mL Teflon vessel equipped with a magnetic stirring bar and placed into a 15 mL autoclave reactor. The lid was closed, and the system pressurized with CO_2 (10 bar) and vented three times. Then, the reactor was pressurized at the selected pressure of 40 bar and placed in a heating mantel set at 130 ºC. After stirring for 24 h, the reactor was cooled down with an ice bath. Mesitylene (10 mol%) was added as an internal standard, and the reaction analyzed by ^1H NMR ($CDCl_3$) to determine the conversion and selectivity. The reaction mixture was purified by column chromatography (8:2 Hex:EtOAc; R_f = 0.30) to give the title compound (183 mg, 69 %) as a colorless oil. ^1H NMR (500 MHz, $CDCl_3$) δ_H = 5.91 – 5.71 (m, 1H), 5.00 – 4.81 (m, 3H), 4.59 (ddt, *J* = 2.7, 1.9, 0.9 Hz, 1H), 4.32 (dd, *J* = 8.5, 2.6 Hz, 1H), 4.08 (dd, *J* = 8.5, 1.2 Hz, 1H), 2.05 – 1.94 (m, 1H), 1.76 – 1.69 (m, 4H), 1.64 – 1.27 (m, 9H), 1.01 (s, 3H); ^{13}C NMR (126 MHz, $CDCl_3$) δ_C = 154.70, 154.68, 149.25, 149.23, 146.82, 146.76, 112.66, 110.57, 110.55, 85.68, 85.65, 73.29, 73.06, 52.00, 51.91, 46.07, 46.02, 39.65, 39.64, 38.93, 38.91, 27.57, 27.41, 24.87, 24.83, 22.11, 21.96, 21.74, 21.63, 16.52; **IR** (neat) ν_{max} = 3079, 29445, 1793 (C=O) cm^{-1}. **HRMS** (ESI^+) $C_{16}H_{24}NaO_3$ $[M+Na]^+$ found 287.1620, requires 287.1618 (0.8 ppm).

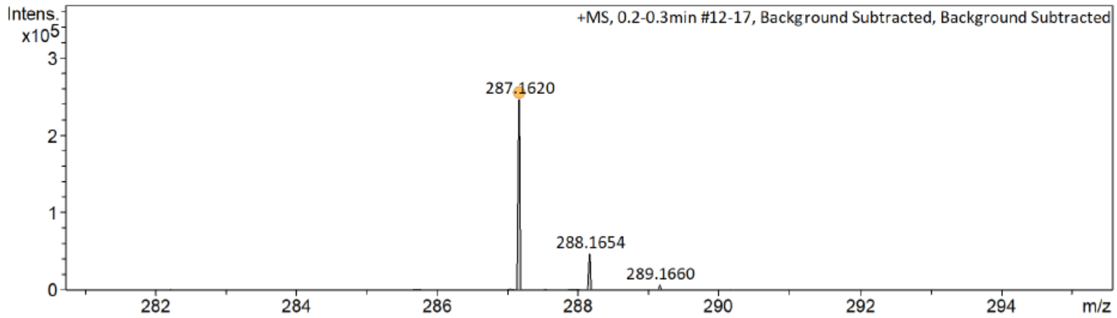

Figure S25. **HRMS** (ESI$^+$) spectrum of β-elemene monocarbonate $C_{16}H_{24}NaO_3$ $[M+Na]^+$ found 287.1620, requires 287.1618 (0.8 ppm).

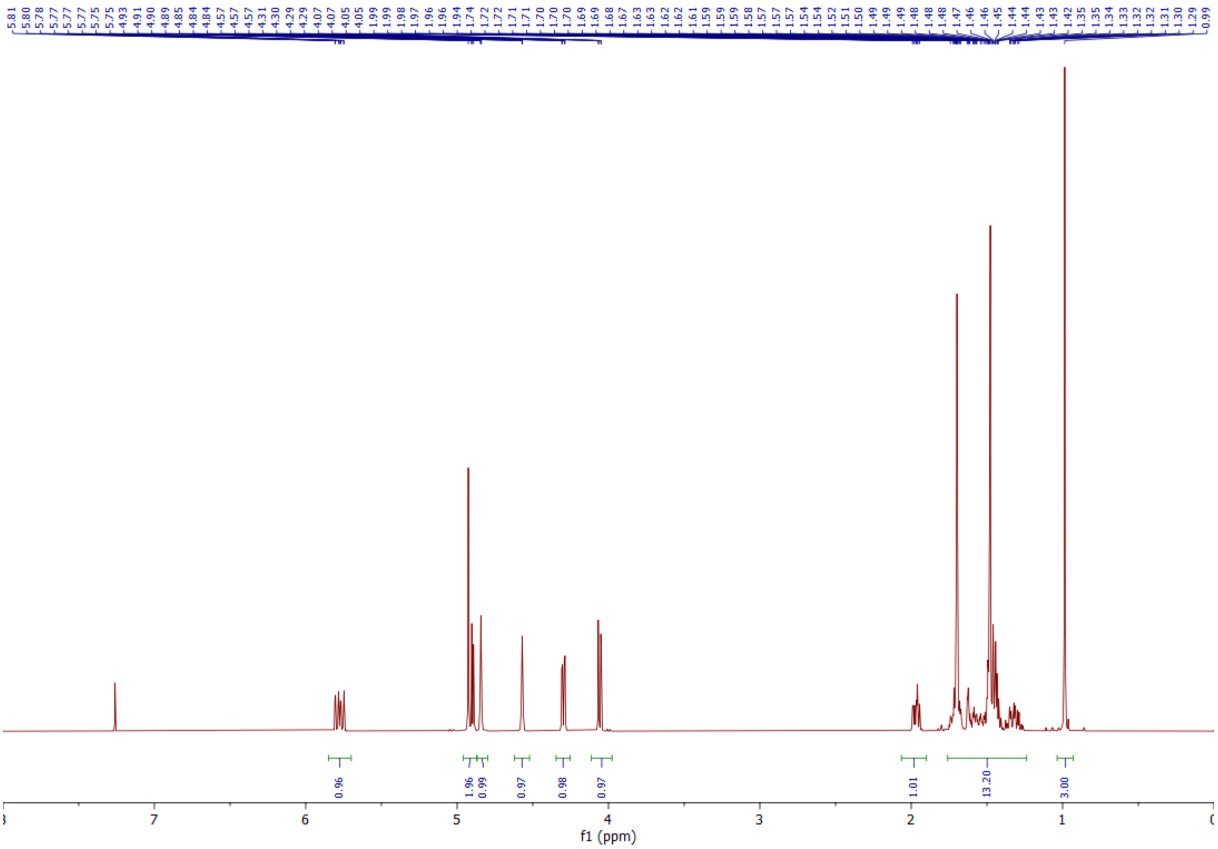

Figure S26. ^{1}H NMR of β-elemene-11,12-carbonate (500 MHz, rt, $CDCl_3$).

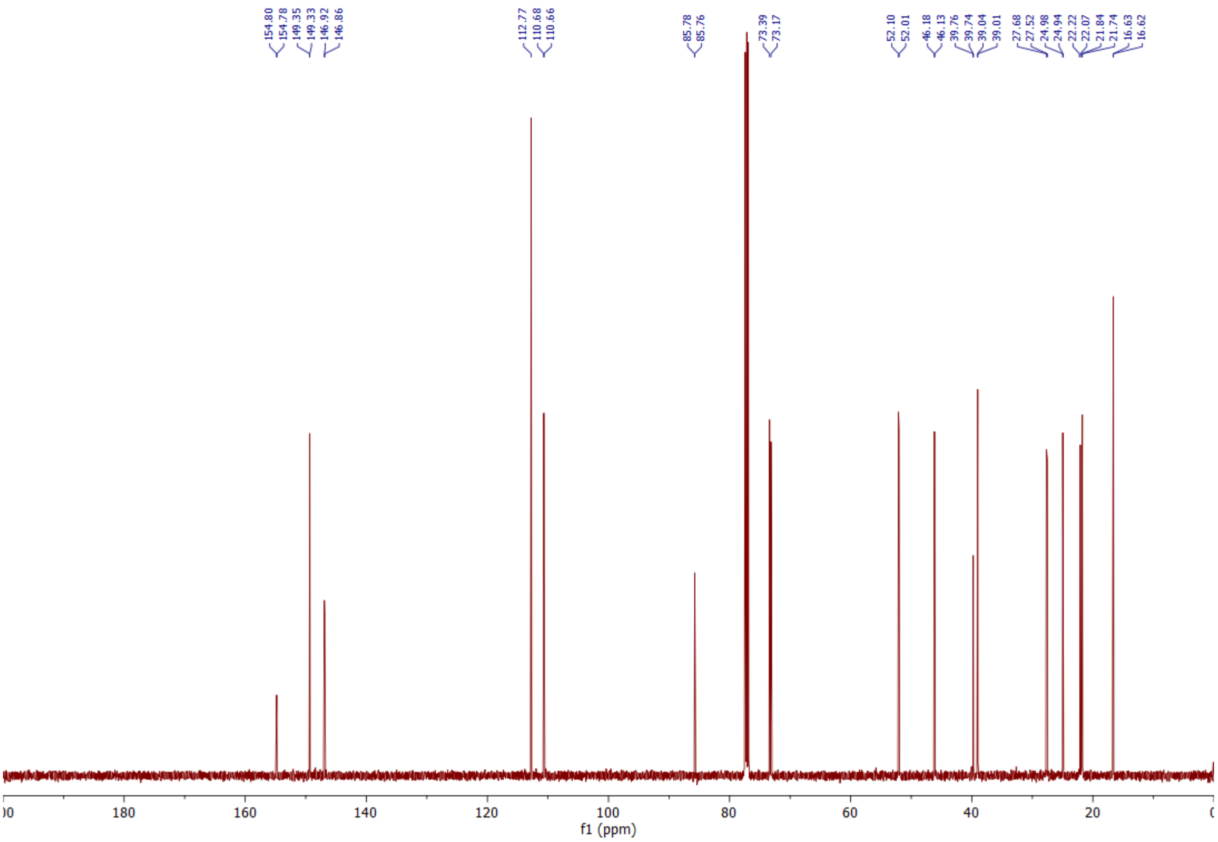

Figure S27. ^{13}C NMR of β-elemene-11,12-carbonate (500 MHz, rt, $CDCl_3$).

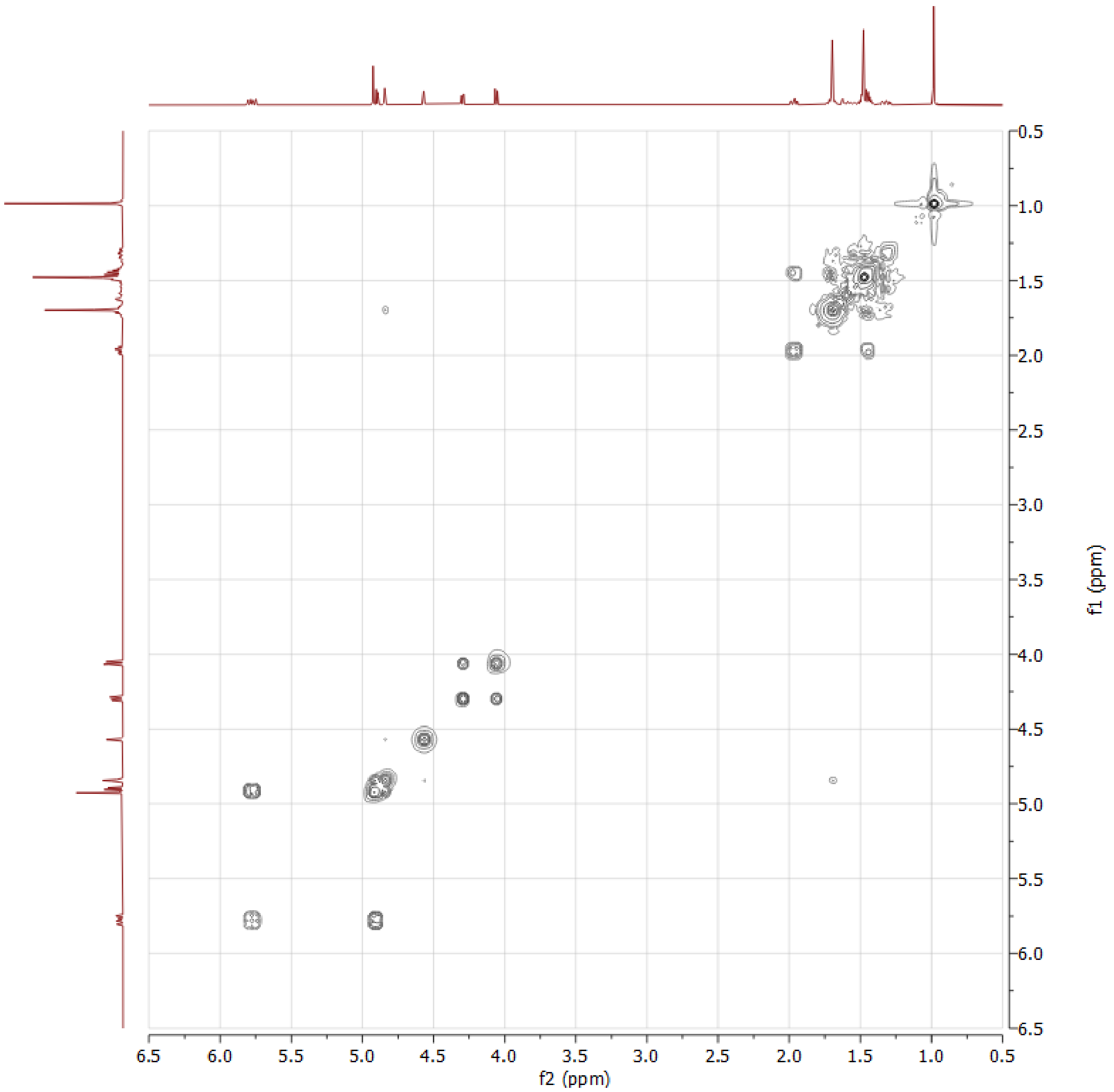

Figure S28. ^{1}H^{1}H-COSY NMR of β-elemene-11,12-carbonate (500 MHz, rt, $CDCl_3$).

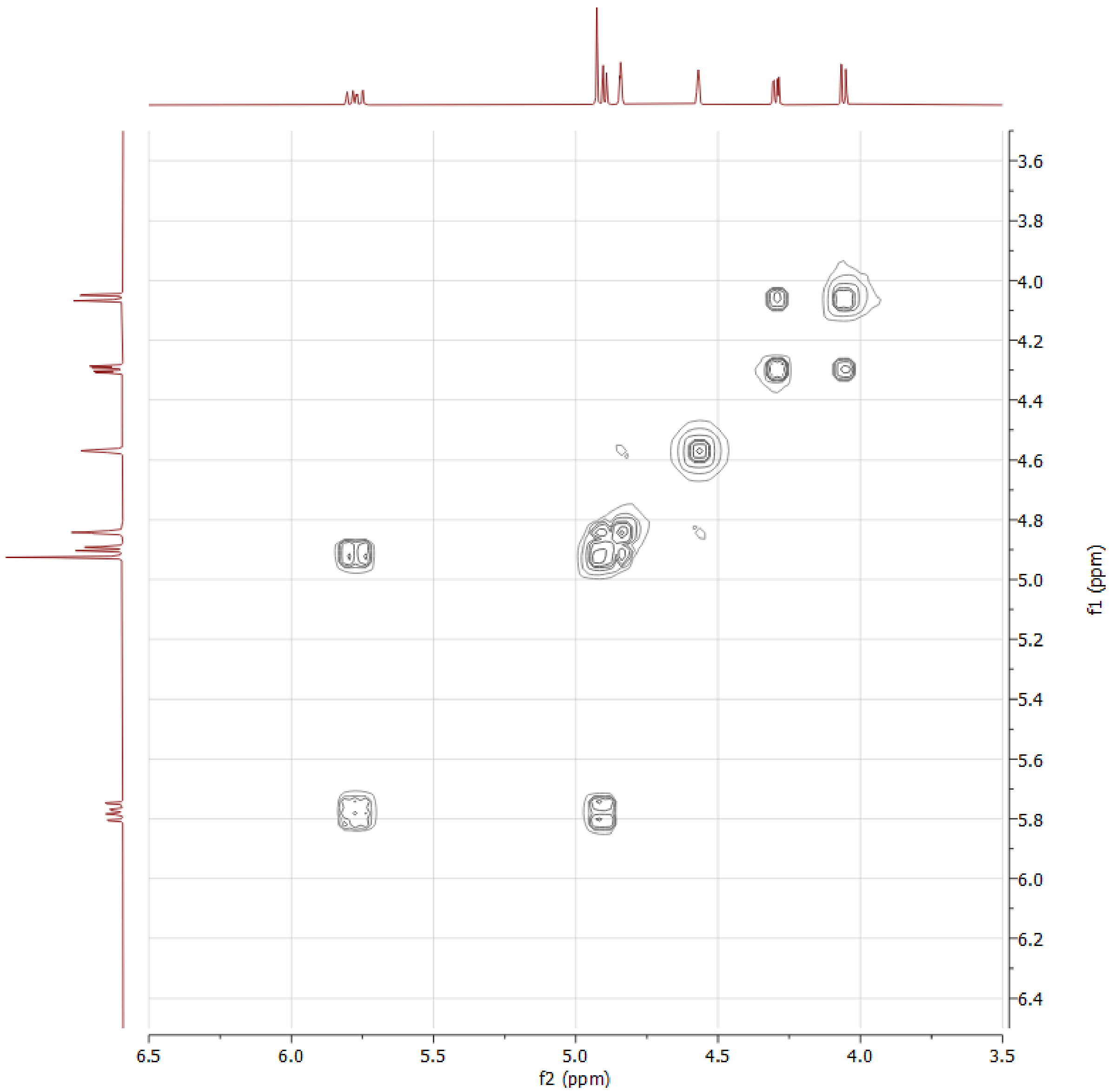

Figure S29. ^{1}H^{1}H-COSY NMR of β-elemene-11,12-carbonate from 6.5 to 3.5 ppm (500 MHz, rt, $CDCl_3$).

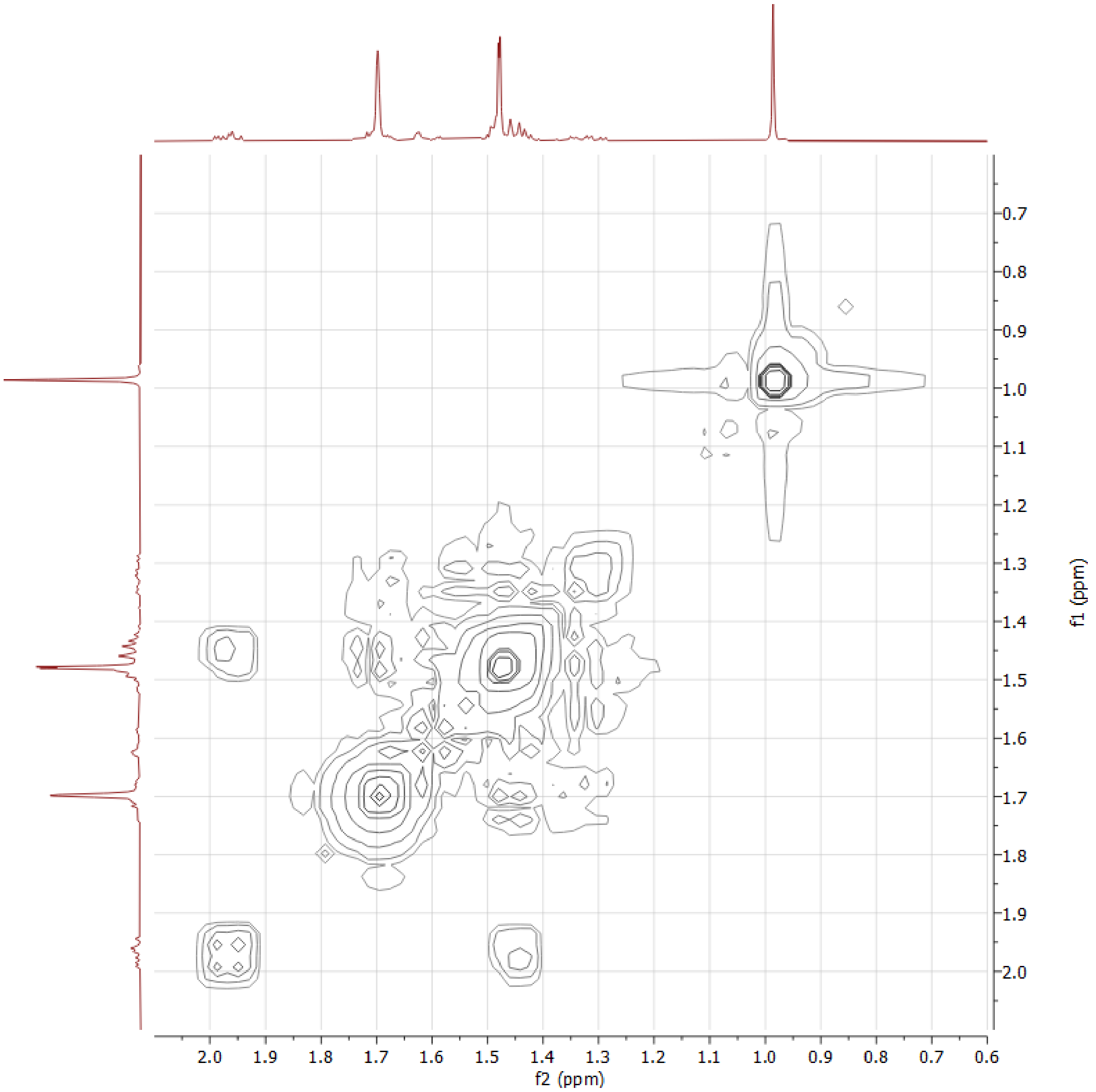

Figure S30. $^1H^1H$-COSY NMR of β-elemene-11,12-carbonate from 2.1 to 0.6 ppm (500 MHz, rt, $CDCl_3$).

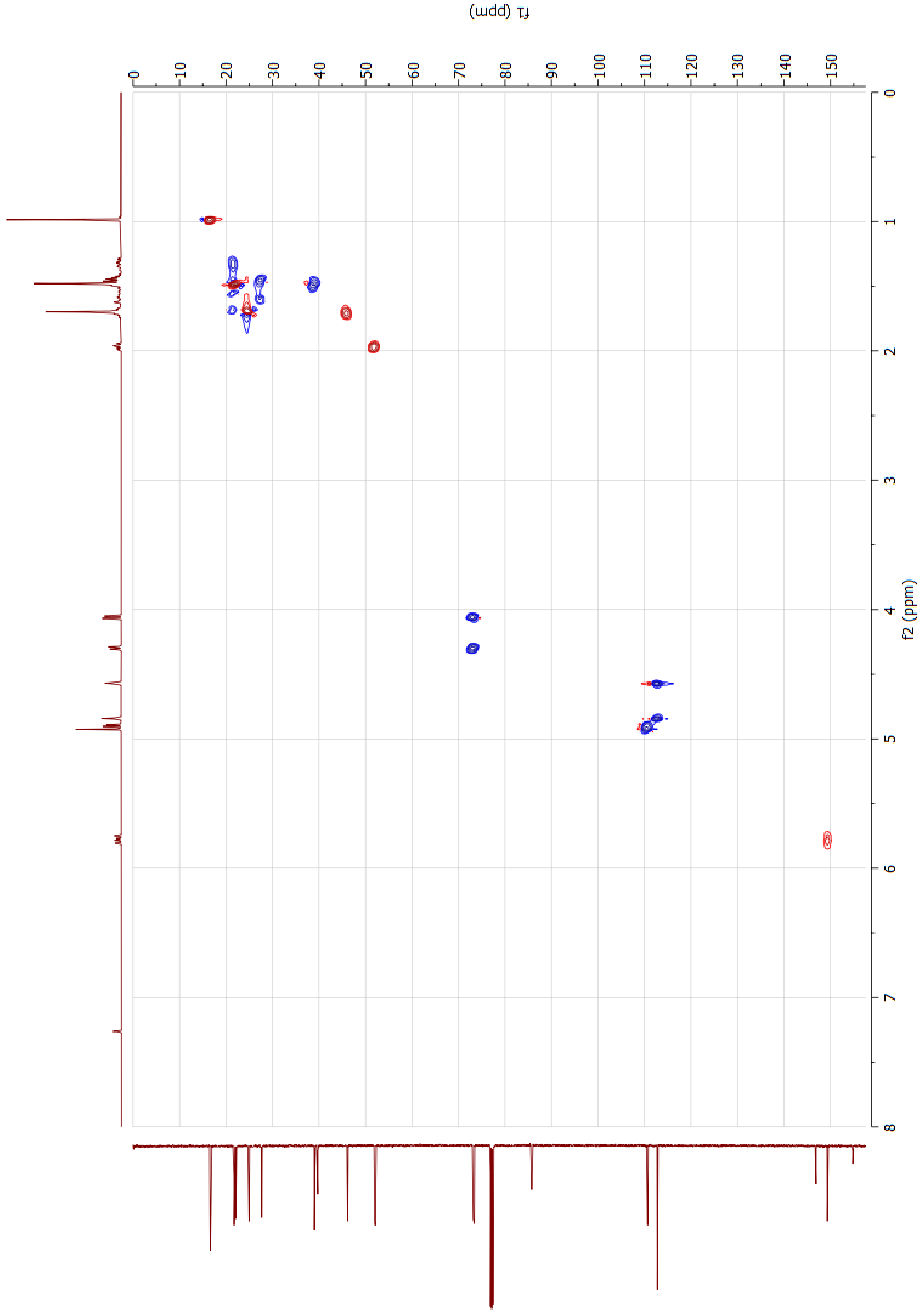

Figure S31. $^{1}H^{13}C$-HSQC NMR of β-elemene-11,12-carbonate (500 MHz, rt, $CDCl_3$).

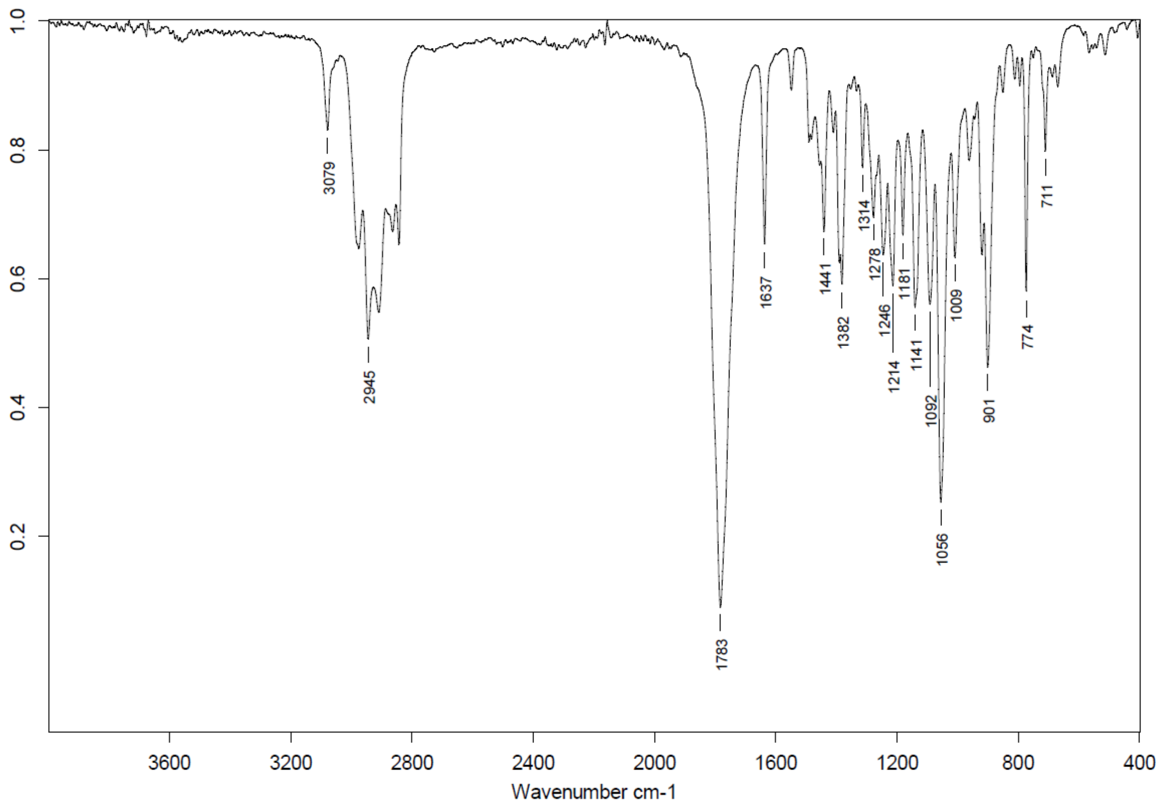

Figure S32. FT-IR of β-elemene dicarbonate monocarbonate.

4-methyl-4-(4-methyl-3-(2-methyloxiran-2-yl)-4-vinylcyclohexyl)-1,3-dioxolan-2-one (BEMC)

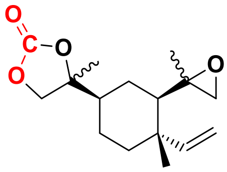

BEMC (264 mg, 1.0 mmol) was dissolved in DCM (10 mL) in a 25 mL round bottom flask and cooled at 0 ºC with an ice bath. *m*CPBA (77 % w/w, 270 mg, 1.2 mol) was added and the reaction mixture stirred at 0 ºC. After 2 hours, the suspension was filtered, the solid washed with hexane and the solvent removed by rotary evaporation. The oil was dissolved in hexane (20 mL) The solution was then washed with an aqueous solution of Na_2SO_3 (3 × 5 mL, 1 M), a saturated aqueous solution of $NaHCO_3$ (3 × 5 mL) and brine. The organic phase was dried over sodium sulfate and the product was obtained after removal of the solvent *in vacuo* using a rotary evaporator. The product was purified through column chromatography (8:2 Hex:EtOAc; R_f = 0.29) to give the title compound (140 mg, 50%) as a colorless oil. (See above for the full characterization).

^{1}H NMR (500 MHz, $CDCl_3$) δ_H= 6.01 – 5.67 (m, 1H), 5.16 – 4.89 (m, 2H), 4.44 – 4.22 (m, 1H), 4.17 – 3.98 (m, 1H), 2.76 – 2.42 (m, 2H), 1.83 – 1.02 (m, 17H); **^{13}C NMR** (126 MHz, $CDCl_3$) δ_C= 154.65, 154.62, 149.62, 149.49, 148.23, 148.19, 111.35, 111.33, 110.45, 110.42, 85.55, 85.47, 85.44, 73.62, 73.31, 73.01, 72.69, 58.26, 58.18, 57.66, 56.31, 56.18, 52.98, 52.94, 52.75, 52.62, 50.49, 50.39, 45.60, 45.57, 45.55, 45.34, 40.67, 40.09, 39.65, 39.06, 39.00, 29.70, 24.24, 23.95, 23.77, 23.62, 23.10, 22.87, 22.72, 22.10, 21.93, 21.83, 21.80, 21.73, 21.43, 21.37, 19.70, 19.63, 17.38, 17.32, 17.02, 16.93; **HRMS** (ESI$^+$) $C_{16}H_{24}NaO_4$ $[M+Na]^+$ found 303.1567, requires 303.1567 (0.0 ppm).

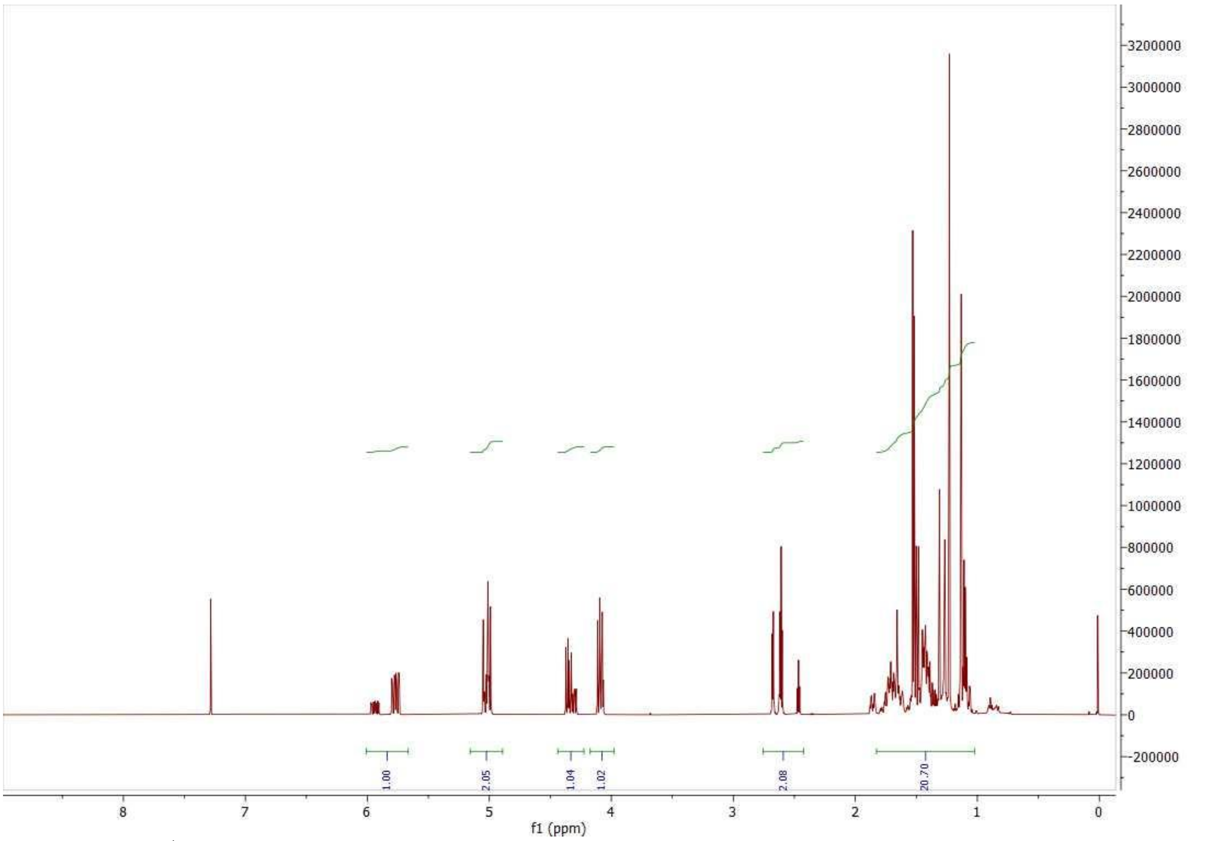

Figure S33. ^{1}H NMR of β-elemene monocarbonate **BEMC** (500 MHz, rt, $CDCl_3$).

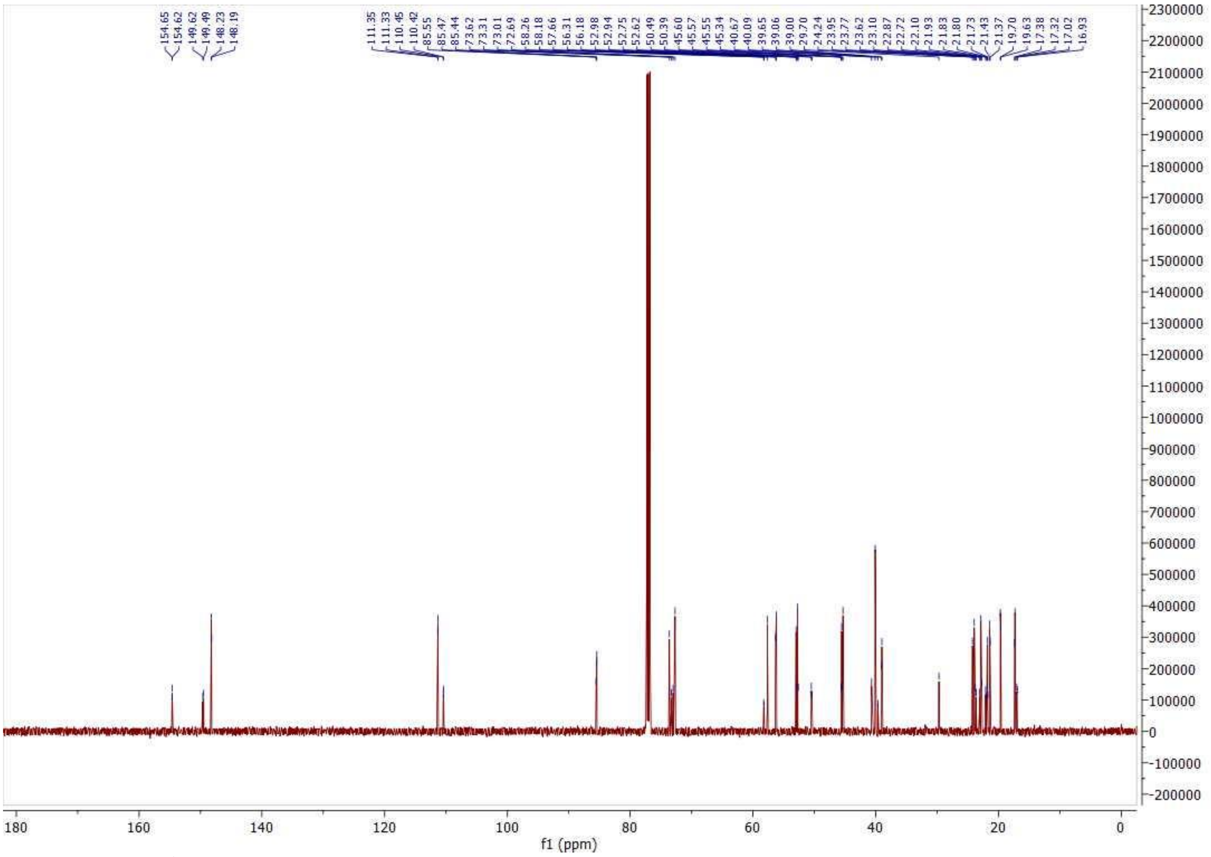

Figure S34. ^{1}H NMR of β-elemene monocarbonate **BEMC** (126 MHz, rt, $CDCl_3$).

S35. General procedure for the urethane synthesis

In a Schlenk tube, both carbonate and the corresponding diamine were combined in a 1:2 ratio (2 equivalent of amine for each carbonate group). After purging with N_2, the reaction mixture was heated up to 100 ºC and let stirring for 24 h. The excess of amine was removed under vacuum and the product was purified through column chromatography. The scale up of **BEDCU6** was realized linearly taking into account the amounts mentioned above.

2-hydroxy-2-(4-methyl-3-(prop-1-en-2-yl)-4-vinylcyclohexyl)propyl hexylcarbamate (BECU1)

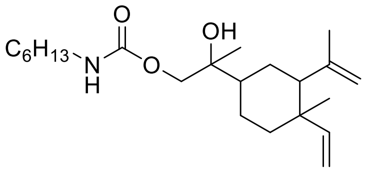

According to the general procedure, **BEC** (50 mg; 0.19 mmol) and hexylamine (50 µL, 0.38 mmol, 2.0 equiv.) were added in a Schlenk tube and stirred for 24 h at 100 ºC. Hereafter, the excess of amine was removed under vacuum and the product was purified through column chromatography (8:2 Cyclohexane:EtOAc) to give the title compound (59 mg, 85%) as a yellowish oil.

^{1}H NMR (500 MHz, $CDCl_3$) δ_H= 5.90 – 5.71 (m, 1H), 4.99 – 4.71 (m, 4H), 4.60 (dd, *J* = 6.9, 2.0 Hz, 1H), 4.07 (dt, *J* = 29.6, 11.3 Hz, 2H), 3.20 (q, *J* = 6.5 Hz, 2H), 2.17 (s, 1H), 1.97 (ddd, *J* = 12.2, 5.3, 3.1 Hz, 1H), 1.72 (d, J = 1.7 Hz, 3H), 1.59 – 1.25 (m, 15H), 1.17 (d, *J* = 1.9 Hz, 3H), 1.00 (s, 3H), 0.94 – 0.87 (m, 3H); **^{13}C NMR** (126 MHz, $CDCl_3$) δ_C= 156.87, 150.08, 147.61, 147.59, 112.20, 112.18, 109.97, 109.95, 73.70, 73.66, 70.52, 52.68, 45.55, 45.46, 41.20, 39.80, 39.75, 31.46, 29.88, 29.70, 28.46, 27.43, 26.41, 24.80, 24.73, 22.70, 22.56, 21.62, 21.21, 20.97, 16.61, 14.01; **IR** (neat) ν_{max} = 3338 (NH, OH), 1696 (C=O) cm^{-1}; **HRMS** (ESI^+) $C_{22}H_{39}NNaO_3$ $[M+Na]^+$ found 388.2818, requires 388.2822 (−0.4 ppm).

2-hydroxy-2-(4-methyl-3-(prop-1-en-2-yl)-4-vinylcyclohexyl)propyl hexyl(methyl)carbamate (BECU2)

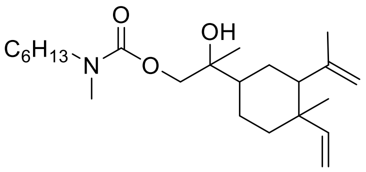

According to the general procedure, **BEC** (50 mg; 0.19 mmol) and N-methylhexylamine (57 µL, 0.38 mmol, 2.0 equiv.) were added in a Schlenk tube and stirred for 24 h at 100 ºC. Hereafter, the excess of amine was removed under vacuum and the product was purified through column chromatography (8:2 Cyclohexane:EtOAc) to give the title compound (60 mg, 82%) as a yellowish oil.

^{1}H NMR (500 MHz, $CDCl_3$) δ_H= 5.91 – 5.74 (m, 1H), 5.00 – 4.79 (m, 3H), 4.60 (ddt, *J* = 7.1, 1.9, 0.8 Hz, 1H), 4.19 – 3.99 (m, 2H), 3.27 (t, *J* = 7.2 Hz, 2H), 2.93 (d, *J* = 1.5 Hz, 3H), 1.96 (ddd, *J* = 11.5, 7.7, 3.0 Hz, 1H), 1.76 – 1.67 (m, 4H), 1.63 – 1.25 (m, 15H), 1.18 (d, *J* = 2.1 Hz, 3H), 1.00 (s, 3H), 0.94 – 0.87 (m, 3H); **^{13}C NMR** (126 MHz, $CDCl_3$) δ_C= 156.90, 150.12, 150.09, 147.64, 147.59, 112.20, 112.16, 109.96, 109.92, 73.72, 73.68, 71.22, 52.82, 52.74, 49.18, 45.78, 39.86, 39.80, 39.76, 31.61, 28.50, 27.48, 26.44, 24.79, 24.71, 22.70, 22.61, 21.66, 21.18, 16.62, 14.03; **IR** (neat) ν_{max} = 3453 (OH), 1682 (C=O) cm^{-1}; **HRMS** (ESI^+) $C_{23}H_{41}NNaO_3$ $[M+Na]^+$ found 402.2971, requires 402.2979 (−0.8 ppm).

2-hydroxy-2-(4-methyl-3-(2-methyloxiran-2-yl)-4-vinylcyclohexyl)propyl hexylcarbamate (BEMCU1)

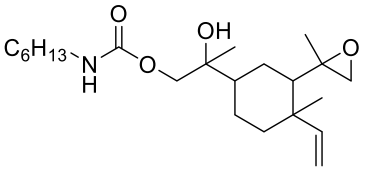

According to the general procedure, **BEMC** (50 mg; 0.18 mmol) and hexylamine (48 µL, 0.36 mmol, 2.0 equiv.) were added in a Schlenk tube and stirred for 24 h at 100 ºC. Hereafter, the excess of amine

was removed under vacuum and the product was purified through column chromatography (7:3 Cyclohexane:EtOAc) to give the title compound (62 mg, 91%) as a yellowish oil.

^{1}H NMR (400 MHz, $CDCl_3$) δ_H= 6.10 – 5.62 (m, 1H), 5.20 – 4.71 (m, 3H), 4.25 – 3.90 (m, 2H), 3.19 (q, *J* = 6.8 Hz, 2H), 2.75 – 2.31 (m, 2H), 1.71 – 1.03 (m, 26H), 0.95 – 0.86 (m, 3H); **^{13}C NMR** (126 MHz, $CDCl_3$) δ_C= 156.84, 150.21, 150.12, 149.11, 110.69, 109.77, 109.72, 73.65, 73.62, 73.60, 73.58, 70.34, 58.78, 58.11, 56.16, 56.13, 53.46, 53.22, 51.53, 44.91, 41.46, 41.43, 41.20, 40.96, 40.90, 39.79, 39.63, 39.20, 39.18, 31.46, 29.87, 26.91, 26.41, 24.87, 24.83, 24.07, 24.02, 22.74, 22.56, 22.54, 22.45, 22.43, 22.13, 21.34, 21.03, 19.82, 19.78, 17.38, 17.37, 17.23, 17.16, 14.01; **IR** (neat) ν_{max} = 3347 (NH and OH), 1682 (C=O) cm^{-1}; **HRMS** (ESI^+) $C_{22}H_{39}NNaO_4$ $[M+Na]^+$ found 404.2768, requires 404.2771 (−0.8 ppm).

2-hydroxy-2-(4-methyl-3-(2-methyloxiran-2-yl)-4-vinylcyclohexyl)propyl hexyl(methyl)carbamate (BEMCU2)

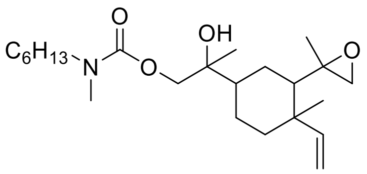

According to the general procedure, **BEMC** (50 mg; 0.18 mmol) and N-methylhexylamine (55 µL, 0.36 mmol, 2.0 equiv.) were added in a Schlenk tube and stirred for 24 h at 100 ºC. Hereafter, the excess of amine was removed under vacuum and the product was purified through column chromatography (8:2 Cyclohexane:EtOAc) to give the title compound (64 mg, 90%) as a yellowish oil.

^{1}H NMR (400 MHz, $CDCl_3$) δ_H= 6.08 – 5.72 (m, 1H), 5.26 – 4.78 (m, 2H), 4.28 – 3.93 (m, 2H), 3.28 (d, *J* = 8.8 Hz, 2H), 2.93 (d, *J* = 1.9 Hz, 3H), 2.76 – 2.35 (m, 2H), 2.12 – 0.68 (m, 29H); **^{13}C NMR** (126 MHz, $CDCl_3$) δ_C= 156.93, 151.43, 149.49, 111.24, 110.81, 73.69, 71.15, 67.56, 53.22, 49.31, 49.07, 47.91, 45.78, 41.02, 39.64, 34.78, 34.02, 31.60, 28.02, 27.41, 26.51, 26.32, 22.60, 21.09, 16.00,

14.03; **IR** (neat) ν_{max} = 3347 (OH), 1684 (C=O) cm^{-1}; **HRMS** (ESI^+) $C_{23}H_{41}NNaO_4$ $[M+Na]^+$ found 418.2922, requires 418.2928 (−0.6 ppm).

(4-methyl-4-vinylcyclohexane-1,3-diyl)bis(2-hydroxypropane-2,1-diyl) bis(hexylcarbamate) (**BEDCU1**)

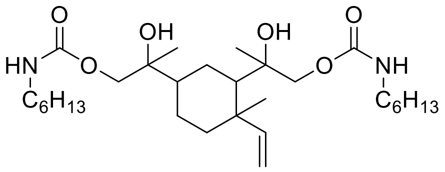

According to the general procedure, **BEDC** (50 mg; 0.15 mmol) and hexylamine (82 µL, 0.6 mmol, 4.0 equiv.) were added in a Schlenk tube and stirred for 24 h at 100 ºC. Hereafter, the excess of amine was removed under vacuum to give the title compound (79 mg, 99%) as a yellow oil.

^{1}H NMR (500 MHz, $CDCl_3$) δ_H= 6.11 – 5.66 (m, 1H), 5.25 – 4.75 (m, 4H), 4.35 – 3.79 (m, 4H), 3.17 (p, *J* = 6.7 Hz, 4H), 2.09 – 1.89 (m, 1H), 1.83 – 0.82 (m, 40H); **^{13}C NMR** (126 MHz, $CDCl_3$) δ_C= 156.98, 156.88, 156.73, 151.71, 151.41, 149.45, 110.78, 109.71, 75.69, 75.60, 73.73, 73.68, 73.61, 70.87, 70.47, 53.15, 45.93, 45.38, 43.89, 43.57, 41.19, 40.17, 40.08, 39.95, 39.94, 39.63, 39.55, 31.47, 31.18, 29.91, 29.87, 27.90, 26.59, 26.43, 26.24, 24.05, 23.31, 23.07, 22.56, 22.42, 22.28, 21.44, 17.58, 17.11, 15.99, 14.02, 13.95; **IR** (neat) ν_{max} = 3331 (NH and OH), 1694 (C=O) cm^{-1}; **HRMS** (ESI^+) $C_{29}H_{54}N_2NaO_6$ $[M+Na]^+$ found 549.3871, requires 549.3874 (−0.6 ppm).

(4-methyl-4-vinylcyclohexane-1,3-diyl)bis(2-hydroxypropane-2,1-diyl) bis(hexyl(methyl)carbamate) (BEDCU2)

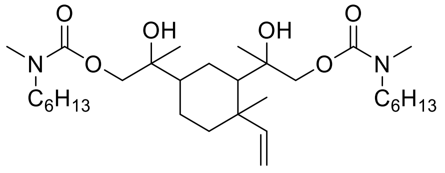

According to the general procedure, **BEDC** (50 mg; 0.15 mmol) and N-methylhexylamine (93 µL, 0.6 mmol, 4.0 equiv.) were added in a Schlenk tube and stirred for 24 h at 100 ºC. Hereafter, the excess of amine was removed under vacuum to give the title compound (83 mg, 99%) as a yellow oil. ^{1}**H NMR** (500 MHz, $CDCl_3$) δ_H= 5.99 (ddd, *J* = 17.6, 10.8, 2.0 Hz, 1H), 5.20 – 4.83 (m, 2H), 4.24 – 3.94 (m, 4H), 3.27 (td, *J* = 7.4, 4.8 Hz, 4H), 3.00 – 2.84 (m, 6H), 2.14 – 1.91 (m, 4H), 1.74 – 1.65 (m, 1H), 1.52 – 1.04 (m, 30H), 0.91 (tt, *J* = 7.0, 3.1 Hz, 6H). ^{13}**C NMR** (101 MHz, CDCl3) δ_C= 156.89, 156.36, 82.08, 79.30, 73.81, 71.58, 68.50, 58.24, 49.15, 43.37, 36.20, 31.61, 27.99, 27.46, 26.39, 25.44, 22.59, 22.02, 20.99, 20.58, 14.03; **IR** (neat) ν_{max} = 3435 (OH), 1684 (C=O) cm^{-1}; **HRMS** (ESI^+) $C_{31}H_{58}N_2NaO_6$ $[M+Na]^+$ found 577.4176, requires 577.4187 (−1.9 ppm).

Full characterization of BEDCU1 synthetized from precipitated BEDC

(4-methyl-4-vinylcyclohexane-1,3-diyl)bis(2-hydroxypropane-2,1-diyl) bis(hexylcarbamate) (BEDCU1)

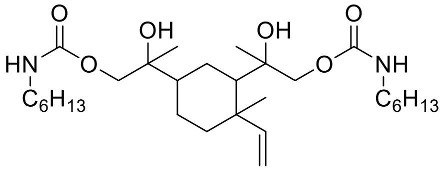

According to the general procedure for urethane synthesis, but using precipitated **BEDC**.

^{1}**H NMR** (500 MHz, $CDCl_3$) δ_H= 5.97 (ddd, *J* = 17.7, 10.8, 2.8 Hz, 1H), 5.27 – 4.72 (m, 4H), 4.29 – 3.83 (m, 4H), 3.19 (p, *J* = 6.9 Hz, 4H), 2.66 – 2.30 (m, 2H), 2.11 – 1.09 (m, 33 H), 0.90 (dt, *J* = 7.0, 3.3 Hz, 6H); ^{13}**C NMR** (126 MHz, $CDCl_3$) δ_C= 156.85, 156.71, 151.69, 109.74, 75.68, 75.59, 73.71,

73.67, 70.84, 70.52, 53.21, 45.49, 43.88, 41.20, 41.16, 39.96, 31.47, 29.91, 29.88, 26.42, 23.96, 23.26, 23.13, 22.57, 20.74, 17.14, 14.02.

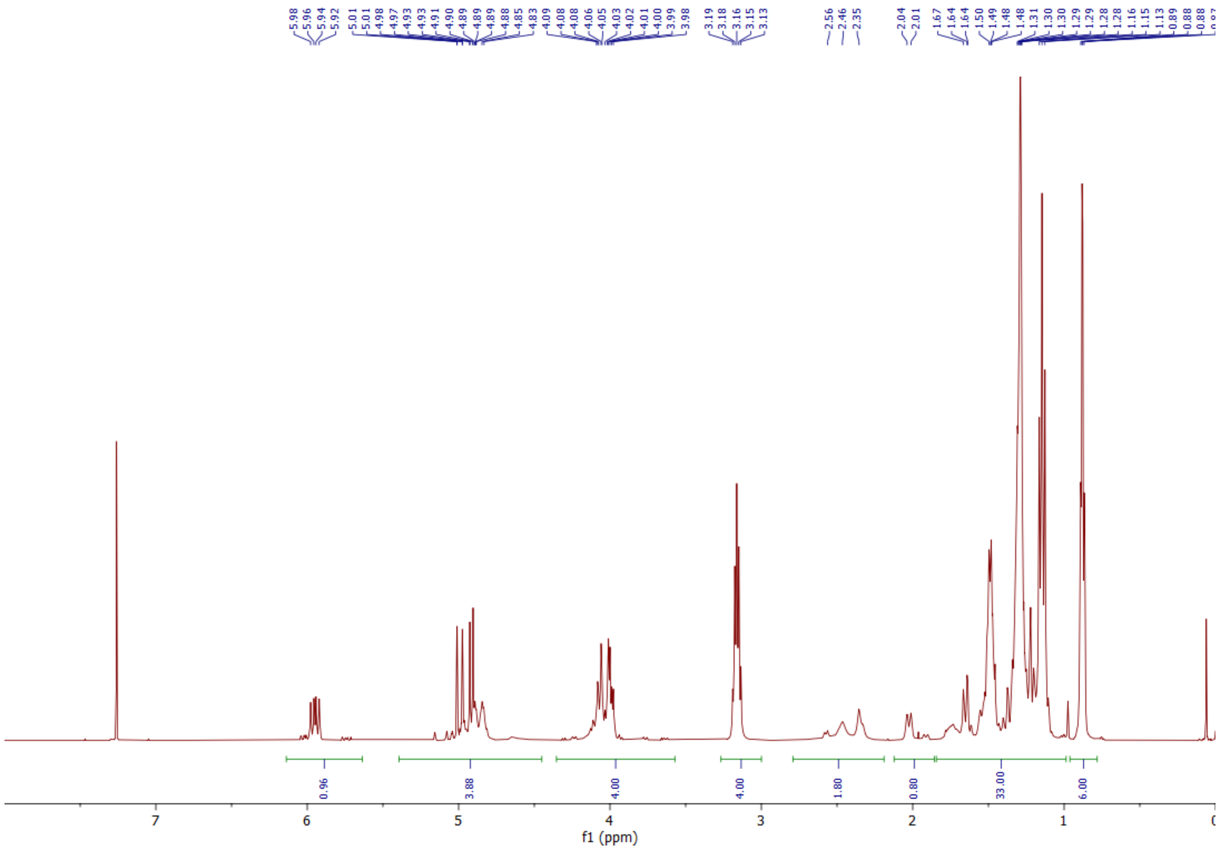

Figure S35. ^{1}H NMR of **BEDCU1** from precipitated **BEDC** (500 MHz, rt, $CDCl_3$).

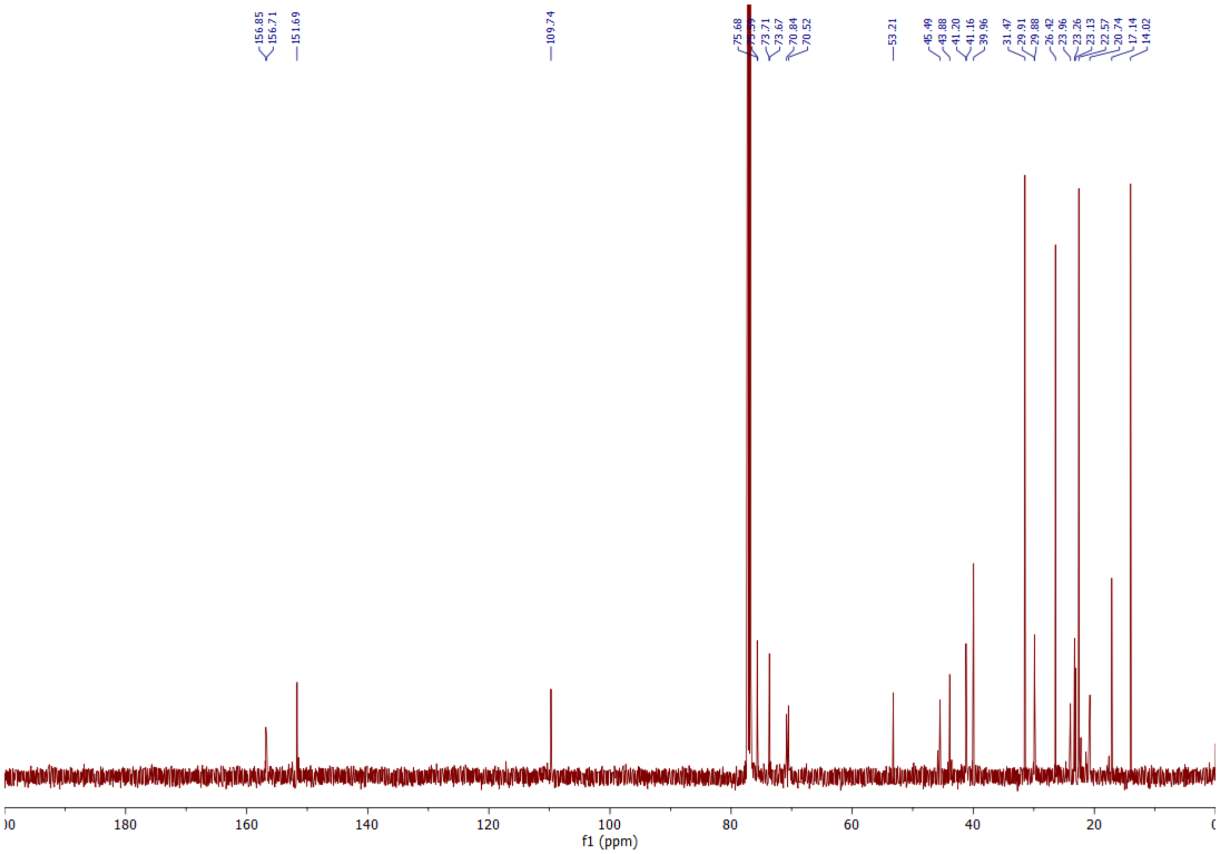

Figure S36. ^{13}C NMR of **BEDCU1** from precipitated **BEDC** (500 MHz, rt, $CDCl_3$).

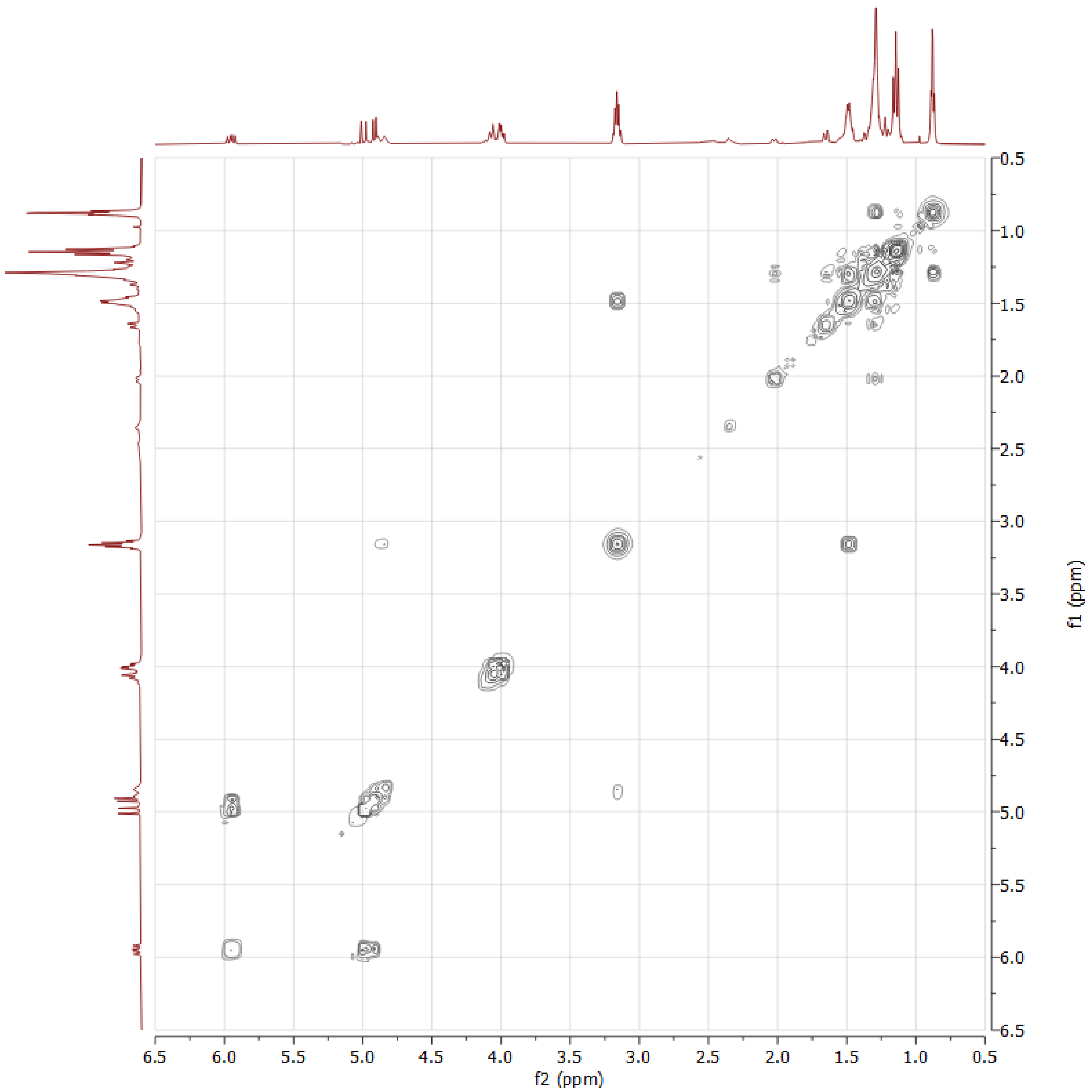

Figure S37. ^{1}H^{1}H-COSY NMR of **BEDCU1** from precipitated **BEDC** (500 MHz, rt, $CDCl_3$).

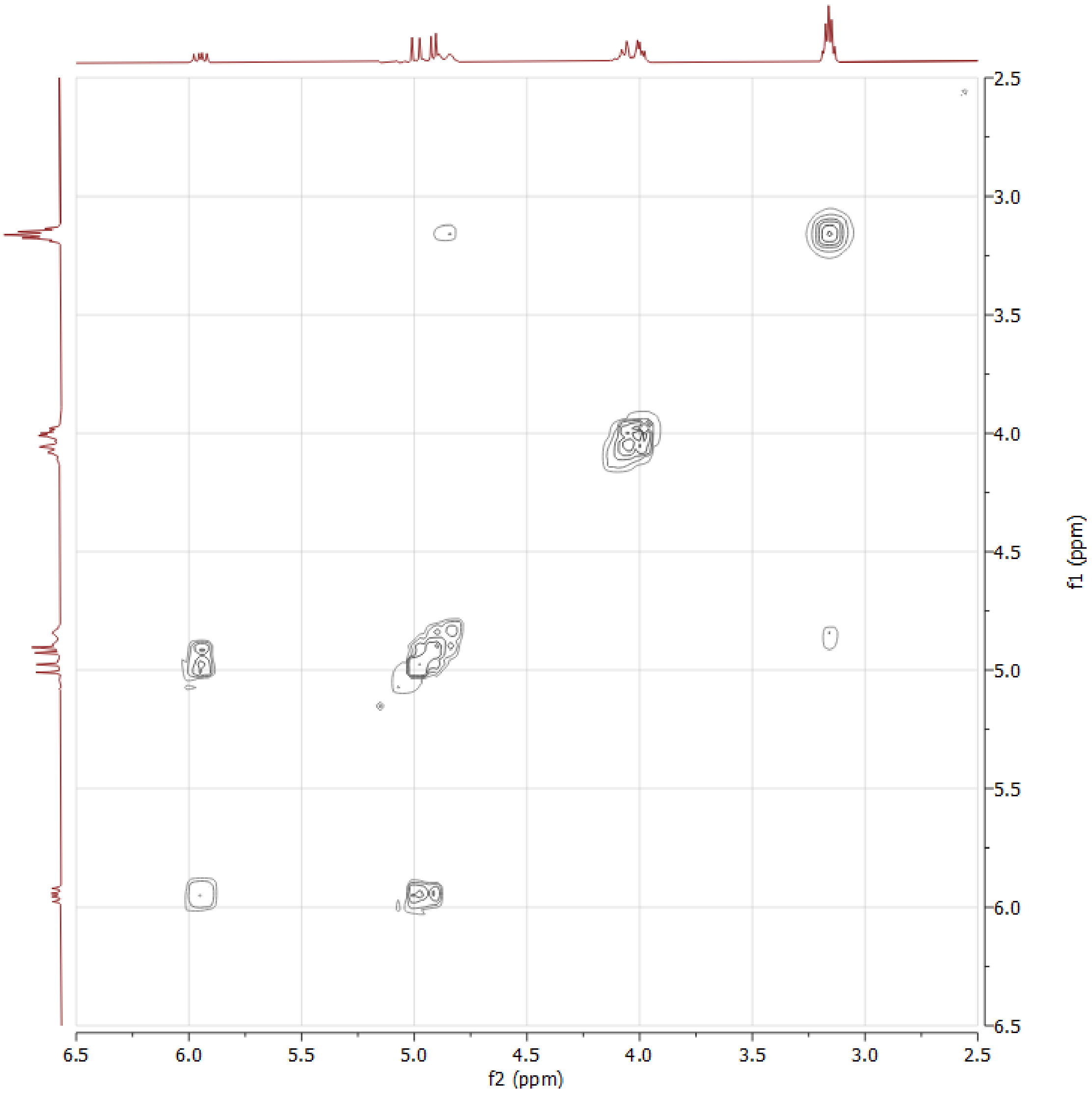

Figure S38. $^{1}H^{1}H$-COSY NMR of **BEDCU1** from precipitated **BEDC** from 6.5 to 2.5 ppm (500 MHz, rt, $CDCl_3$).

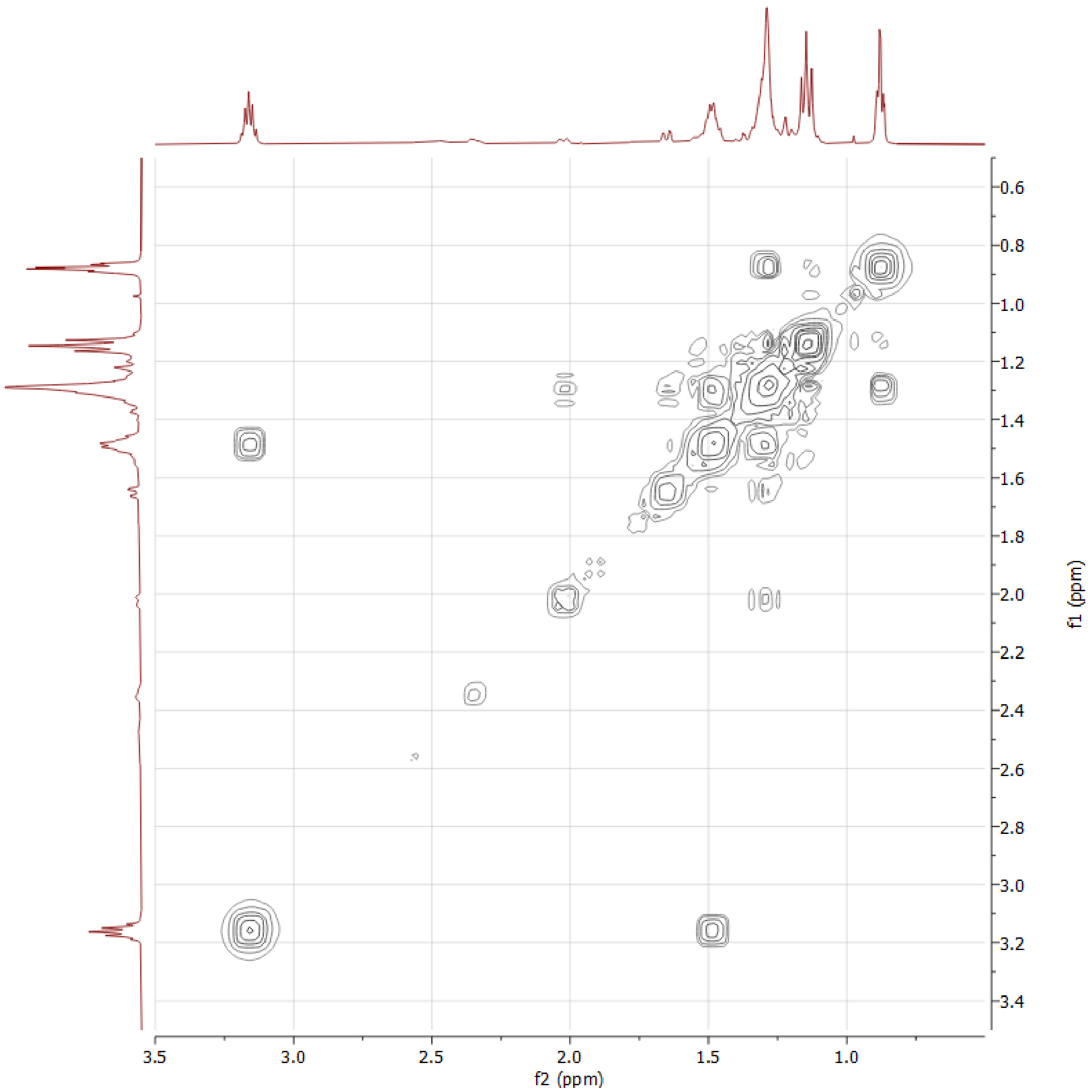

Figure S39. ^{1}H^{1}H-COSY NMR of **BEDCU1** from precipitated **BEDC** from 3.5 to 0.5 ppm (500 MHz, rt, $CDCl_3$).

S45. General procedure for NIPU synthesis using various diamines and BEDC

In a Schlenk tube, both **BEDC** and the corresponding diamine were combined in a 1:1 ratio. After purging with N_2, the reaction mixture was heated up to 130 ºC and let stirring for 48 h. Hereafter, the reaction mixture was allowed to reach room temperature, dissolved in MeOH and precipitated by addition of hexane. This work up procedure was repeated three times and the precipitated polymer was then dried under vacuum. For the M_n, $Đ$ and T_g values of all isolated NIPU oligomers, see Table 3.

BEDCU3

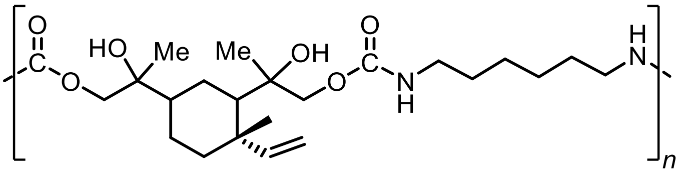

According to the general procedure, **BEDC** (1.0 g, 3.0 mmol) and diamine **A1** (420 mL, 1 equiv) were added in a Schlenk tube and stirred for 48 h at 130 ºC. Hereafter, the reaction mixture was allowed to reach room temperature, dissolved in MeOH and precipitated by addition of hexane. This work up procedure was repeated three times and the precipitated polymer was then dried under vacuum affording the product as a orange sticky oil (1.12 g, 83%, M_n = 1.6 kg/mol, $Đ$ = 1.5).

^{1}H NMR (500 MHz, DMSO-d_6) δ_H= 6.02 – 5.62 (m, 1H), 5.15 – 4.60 (m, 2H), 4.19 – 3.55 (m, 4H), 3.03 – 2.66 (m, 4H), 1.89 – 0.81 (m, 24H); **^{13}C NMR** (125 MHz, DMSO-d_6) δ_C= 156.82, 154.81, 154.38, 153.59, 153.37, 152.73, 152.68, 152.13, 151.66, 150.68, 150.21, 149.64, 110.57, 110.48, 110.00, 109.95, 108.18, 87.79, 87.77, 87.57, 86.48, 86.32, 86.29, 86.23, 75.08, 74.66, 74.26, 74.23, 74.05, 72.85, 72.49, 72.39, 72.24, 72.20, 72.16, 70.77, 69.76, 69.58, 69.40, 69.24, 65.63, 52.34, 51.86, 51.46, 50.91, 47.67, 46.71, 45.76, 45.66, 45.61, 45.18, 44.84, 44.48, 44.25, 44.08, 42.98, 41.23, 29.88, 29.72, 26.44, 26.25, 26.06, 25.85, 24.47, 23.06, 22.83, 22.64, 22.51, 22.37, 22.25, 22.13, 21.99, 21.86,

21.80, 21.64, 21.50; **IR** (neat) ν_{max} = 3339 (NH and NH_2), 2931, 2863, 1790 (carbonate), 1692 (C=O amide), 1637, 1520 (NH amide), 1460, 1379, 1251 (C−O urethane), 1058, 906, 774, 668 cm^{-1}.

BEDCU4

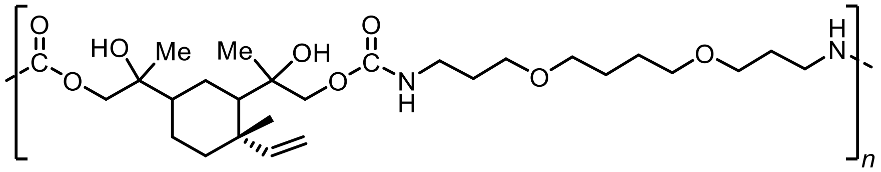

According to the general procedure, **BEDC** (1.0 g, 3.0 mmol) and diamine **A2** (612 mg, 1 equiv) were added in a Schlenk tube and stirred for 48 h at 130 ºC. Hereafter, the reaction mixture was allowed to reach room temperature, dissolved in MeOH and precipitated by addition of hexane. This work up procedure was repeated three times and the precipitated polymer was then dried under vacuum affording the product as an orange sticky oil (1.2 g, 76%, M_n = 1.8 kg/mol, *Đ* = 1.5).

^{1}H NMR (500 MHz, DMSO-d_6) δ_H= 6.16 – 5.69 (m, 1H), 5.09 – 4.38 (m, 2H), 4.21 – 3.55 (m, 4H), 3.51 – 3.12 (m, 16H), 3.06 – 2.74 (m, 4H), 1.90 – 0.81 (m, 21H); **^{13}C NMR** (125 MHz, DMSO-d_6) δ_C= 169.74, 169.71, 158.87, 158.84, 156.87, 156.84, 154.81, 154.74, 154.45, 154.39, 152.74, 152.64, 152.58, 151.59, 150.97, 150.87, 150.70, 150.34, 150.17, 149.61, 149.58, 110.91, 110.49, 110.38, 110.29, 109.47, 109.34, 87.84, 87.78, 87.75, 87.53, 87.49, 86.45, 86.26, 86.24, 86.09, 75.57, 75.26, 75.15, 75.09, 74.23, 73.76, 73.71, 73.52, 73.49, 73.39, 73.07, 72.90, 72.42, 72.26, 72.22, 72.18, 70.48, 70.38, 70.31, 70.29, 69.62, 69.24, 69.20, 68.22, 68.20, 68.15, 68.07, 67.96, 67.82, 67.48, 67.45, 67.38, 65.65, 65.62, 61.18, 61.00, 60.97, 52.31, 52.25, 51.84, 48.99, 47.75, 47.67, 46.68, 46.65, 46.59, 46.56, 45.73, 45.65, 45.60, 44.89, 44.82, 44.72, 44.57, 44.44, 44.35, 44.30, 44.20, 44.00, 43.74, 43.28, 43.11, 43.09, 42.98, 41.95, 40.46, 40.37, 40.29, 40.20, 40.12, 40.03, 39.95, 39.87, 39.78, 39.70, 39.63, 39.59, 39.53, 39.36, 38.82, 38.62, 38.09, 38.04, 37.03, 37.01, 36.98, 36.35, 34.92, 32.96, 31.42, 30.83, 30.71, 30.14, 29.81, 29.72, 29.66, 29.62, 29.60, 28.75, 27.88, 27.80, 27.73, 27.71, 26.49, 26.47, 26.44, 26.41, 26.38, 26.34, 26.27, 25.58, 25.54, 25.42, 24.91, 24.79, 24.71, 24.66, 24.62, 24.58, 24.52, 24.47, 24.43, 24.38, 23.24, 23.05, 22.99, 22.92, 22.86, 22.71, 22.64, 22.53, 22.45, 22.29, 22.24, 22.14, 22.03, 21.95,

21.87, 21.82, 21.80, 21.77, 21.74, 21.63, 21.48, 21.43, 21.40, 21.37, 18.26, 17.35, 17.32, 17.25, 17.20, 17.17, 17.12, 16.75, 16.69, 16.49, 16.46, 16.41, 14.38; **IR** (neat) ν_{max} = 3336 (NH and NH_2), 2936, 2862, 1794 (carbonate), 1692 (C=O amide), 1542 (NH amide), 1465, 1376, 1252 (C−O urethane), 1105, 1059, 906, 774 cm^{-1}.

BEDCU5

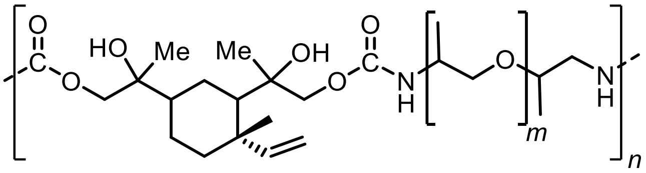

According to the general procedure, **BEDC** (1.0 g, 3.0 mmol) and diamine **A3** (1.2 g, 1 equiv) were added in a Schlenk tube and stirred for 48 h at 130 ºC. Hereafter, the reaction mixture was allowed to reach room temperature, dissolved in MeOH and precipitated by addition of hexane. This work up procedure was repeated three times and the precipitated polymer was then dried under vacuum affording the product as a brownish sticky oil (1.66 g, 74%, M_n = 2.0 kg/mol, *Đ* = 1.6).

^{1}H NMR (500 MHz, DMSO-d_6) δ_H= 6.13 – 5.55 (m, 1H), 5.17 – 4.58 (m, 2H), 4.51 – 3.09 (m, 4H), 1.55 – 1.26 (m, 4H), 1.23 – 0.57 (m, 21H); **^{13}C NMR** (125 MHz, DMSO-d_6) δ_C= 156.19, 154.72, 154.68, 154.20, 152.12, 152.07, 150.98, 150.06, 150.00, 149.65, 149.63, 110.95, 110.39, 109.97, 109.94, 87.83, 87.67, 87.51, 87.48, 87.39, 86.25, 86.23, 86.18, 86.11, 86.08, 75.63, 75.14, 75.05, 74.94, 74.83, 74.81, 74.70, 73.54, 73.38, 73.33, 72.93, 72.91, 72.77, 72.72, 72.55, 71.24, 68.14, 67.87, 67.46, 65.76, 65.68, 65.65, 52.32, 51.83, 51.46, 51.45, 50.94, 50.86, 49.02, 47.10, 46.96, 46.90, 46.71, 46.68, 45.67, 45.63, 45.36, 45.18, 44.93, 44.77, 44.76, 43.77, 43.10, 43.01, 41.99, 41.97, 41.94, 38.85, 38.83, 31.43, 27.82, 27.73, 25.58, 24.62, 24.48, 24.37, 23.02, 22.92, 22.83, 22.58, 22.52, 22.48, 22.45, 22.24, 22.01, 21.97, 21.91, 21.82, 21.80, 21.70, 21.65, 21.55, 21.50, 21.47, 21.41, 20.70, 18.76, 18.57, 18.38, 17.75, 17.69, 17.63, 17.53, 17.51, 16.77, 16.75, 16.71, 16.49, 16.45, 16.32, 16.21, 14.3; **IR**

(neat) ν_{max} = 3339 (NH and NH_2), 2924, 1790 (carbonate), 1695 (C=O amide), 1537 (NH amide), 1462, 1385, 1241 (C−O urethane), 1059, 905, 774 cm^{-1}.

BEDCU6

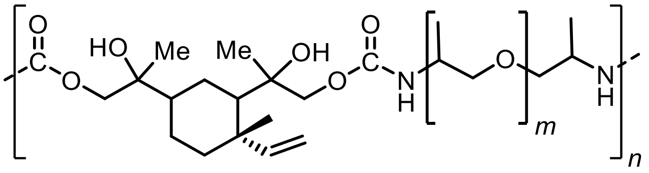

According to the general procedure, **BEDC** (1.0 g, 3.0 mmol) and diamine **A3** (6.0 g, 1 equiv) were added in a Schlenk tube and stirred for 48 h at 130 ºC. Hereafter, the reaction mixture was allowed to reach room temperature, dissolved in MeOH and precipitated by addition of hexane. This work up procedure was repeated three times and the precipitated polymer was then dried under vacuum affording the product as a brownish sticky oil (4.00 g, 57%, M_n = 6.3 kg/mol, *Đ* = 2.1).

^{1}H NMR (500 MHz, DMSO-d_6) δ_H= 6.03 – 5.59 (m, 1H), 5.11 – 4.67 (m, 2H), 4.54 – 3.65 (m, 4H), 3.61 – 3.14 (m, 19H), 2.01 – 0.61 (m, 33H) ppm; **^{13}C NMR** (125 MHz, DMSO-d_6) δ_C= 157.57, 156.10, 154.80, 154.79, 154.73, 154.70, 154.43, 154.37, 154.26, 154.22, 154.21, 153.39, 152.92, 152. 84, 152.72, 152.67, 152.11, 152.06, 151.28, 150.96, 150.87, 150.70, 150.21, 150.04, 149.98, 149.66, 149.63, 132.39, 126.79, 110.99, 110.96, 110.56, 110.48, 110.43, 110.36, 109.98, 109.95, 109.45, 109.25, 87.86, 87.85, 87.79, 87.77, 87.59, 87.56, 87.54, 87.53, 87.51, 87.48, 87.41, 86.74, 86.57, 86.53, 86.47, 86.29, 86.26, 86.24, 86.20, 86.14, 86.11, 75.51, 75.29. 75.23, 75.10, 75.04, 74.32, 74.09, 73.63, 73.48, 73.44, 73.41, 73.40, 73.30, 73.25, 73.12, 73.01, 72.89, 72.84, 72.48, 72.33, 72.23, 70.82, 68.13, 68.04, 67.86, 67.78, 66.99, 65.66, 65.63, 63.47, 63.38, 61.38, 60.20, 55.93, 54.82, 54.54, 52.69, 52.32, 52.27, 52.21, 51.81, 51.46, 51.44, 50.92, 50.85, 48.23, 47.76, 47.75, 47.66, 47.58, 47.53, 47.44, 47.08, 46.94, 46.71, 46.67, 46.53, 45.75, 45.66. 45.62, 44.92, 44.91, 44.79, 44.76. 44.66, 44.61, 44.45, 43.76, 43.51, 43.30, 43.12, 43.09, 42.99, 41.98, 41.95, 41.23, 41.05, 40.76, 38.85, 38.83, 37.88, 36.86, 36.73, 36.52, 36.23, 36.10, 36.05, 35.47, 35.33, 35.30, 35.27, 31.98, 31.91, 31.87, 31.78, 31.76, 31.74,

31.70, 30.33, 30.08, 29.75, 28.51, 27.98, 27.89, 27.86, 27.84, 27.82, 27.72, 27.32, 26.66, 25.88, 25.56, 25.34, 25.24, 24.86, 24.69, 24.62, 24.56, 24.47, 24.37, 24.25, 23.64, 23.59, 23.47, 23.37, 23.02, 22.93, 22.81, 22.61, 22.58, 22.47, 22.44, 22.36, 22.25, 22.14, 22.04, 21.98, 21.95, 21.85, 21.80, 21.77, 21.70, 21.64, 21.57, 21.50, 21.47, 21.40, 21.12, 17.53, 17.26, 17.18, 17.05, 16.80, 16.78, 16.76, 16.75, 16.62, 16.54, 16.53, 16.50, 16.48, 16.46, 16.25, 14.55; **IR** (neat) ν_{max} = 3348 (NH and NH2), 2971, 2870, 1796 (carbonate), 1714, 1650, 1537 (NH amide), 1455, 1375, 1258 (C−O urethane), 1089, 1059, 909, 775 cm^{-1}.

BEDCU7

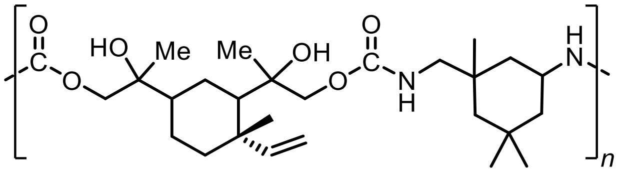

According to the general procedure, **BEDC** (1.0 g, 3.0 mmol) and diamine **A3** (553 mL, 1 equiv) were added in a Schlenk tube and stirred for 48 h at 130 ºC. Hereafter, the reaction mixture was allowed to reach room temperature, dissolved in MeOH and precipitated by addition of hexane. This work up procedure was repeated three times and the precipitated polymer was then dried under vacuum affording the product as a brownish sticky oil (1.39 g, 92%, M_n = 1.3 kg/mol, $Đ$ = 1.3).

^{1}H NMR (500 MHz, DMSO-d_6) δ_H= 5.98 – 5.65 (m, 1H), 5.11 – 4.66 (m, 2H), 4.52 – 3.71 (m, 4H), 3.69 – 3.57 (m, 4H), 3.56 – 3.13 (m, 84H); **^{13}C NMR** (125 MHz, DMSO-d_6) δ_C= 157.42, 156.22, 154.81, 154.79, 154.74, 154.70, 154.44, 154.42, 154.40, 154.36, 154.30, 154.26, 154.22, 154.21, 152.12, 152.07, 151.25, 150.98, 150.89, 150.39, 150.05, 150.00, 149.66, 149.64, 111.02, 110.98, 110.47, 110.34, 110.29, 109.97, 109.94, 109.75, 87.87, 87.85, 87.80, 87.77, 87.75, 87.55, 87.51, 87.49, 87.42, 86.29, 86.27, 86.25, 86.21, 86.15, 86.12, 75.36, 75.31, 75.29, 75.23, 75.15, 75.09, 75.04, 74.99, 74.96, 74.92, 74.83, 74.68, 73.49, 73.43, 73.41, 73.40, 73.30, 73.11, 73.01, 72. 90, 72.85, 72.63, 72.55, 70.65, 69.21, 68.13, 68.05, 67.87, 65.76, 65.68, 65.66, 65.63, 52.69, 52.31, 52.26, 51.81, 51.45,

51.44, 50.92, 50.85, 47.75, 47.14, 47.00, 46.92, 46.71, 46.67, 45.66, 45.62, 45.37, 45.18, 44.92, 44.76, 44.74, 44.30, 44.19, 43.76, 43.31, 43.12, 43.09, 42.99, 41.98, 41.95, 38.85, 38.83, 27.81, 27.72, 25.86, 25.67, 25.54, 24.91, 24.86, 24.72, 24.63, 24.48, 24.38, 23.02, 22.83, 22.58, 22.47, 22.25, 22.14, 22.03, 21.97, 21.94, 21.84, 21.81, 21.70, 21.64, 21.56, 21.50, 21.46, 21.40, 20.75, 18.71, 18.36, 18.04, 17.72, 17.64, 17.60, 17.54, 17.50, 17.40, 17.17, 17.05, 16.79, 16.78, 16.76, 16.74, 16.73, 16.52, 16.50, 16.46, 16.06, 15.91, 15.79; **IR** (neat) ν_{max} = 3310 (NH and NH_2), 2970, 2886, 1803 (carbonate), 1453, 1373, 1093, 925 cm^{-1}. Other representative signals were not clearly observed by IR.

S51. Thermal curing

Thiol-ene reactions

In 10 vials, 100 mg of the **BEDCU6** was charged and then AIBN (2 mg, 2 wt.%) was added as initiator, and hereafter the desired amount of linker. Three out of these ten vials were mixed with a 1:1 ratio of alkene:thiol (that means 1 equiv of alkene to ¼ equiv of **PETMP**, 5.2 mg), three of them with a 1:1.2 ratio (i.e., 6.2 mg **PETMP**) and the other three with a 1:0.8 ratio (i.e., 4.2 mg **PETMP**). The remaining vial was used as a blank (absence of linker). The reaction mixtures were poured into an aluminum tray providing a homogeneous film. Then, temperatures of 100 ºC, 120 ºC and 140 ºC were screened for the three ratios of alkene : thiol used. After cooking for 40 minutes, the trays were allowed to cool down and the dryness of the films obtained in all experiments was evaluated.

In the case of **GDMP**, the reaction conditions were 140 ºC during 40 min, using 2 wt % AIBN, and functional group ratios of 1:0.8 (4.1 mg **GDMP**), 1:1 (5.1 mg **GDMP**) and 1:1.2 (6.1 mg **GDMP**). Further on, the procedure followed was the same as explained above for those reactions done with **PETMP**.

Dual Curing

BEDCU6 (73 wt%) was premixed with Irgacure® 2100 (7 wt%, photo-initiator) and with Laromer® 9000 in ratio as reported in Table S1. The formulations were put in a vial and dissolved in 20 wt % of methyl isobutyl ketone (MIBK) and later applied onto a metallic panel employing a spiral squeegee or coating knife with a certain thickness associated. Then, the wet film was cured for 5 min under UV irradiation and subsequently heated for 20 min in an oven at 140 ºC. The coatings produced were thus subjected to the following physical tests.

Table S1. Formulations used for the coating preparation from **BEDCU6** and Laromer® 9000.

Entry	Code	**BEDCU6** (wt %)	Laromer (wt %)
1	Formulation 1	73	0
2	Formulation 2	53	20
3	Formulation 3	43	30
4	Formulation 4	0	73

S53. Physical tests

All the physical tests performed at BASF are optimized for the testing of coatings applied in automotive industry. Their procedures are reported below while all the data are gathered in Table 3.

Drying test

An "L" shape metal piece is placed in the middle of the panel with a 100 g weight for 20 sec. The weight is removed after 20 sec which triggers a toppling of the L-shaped metal piece. The drying test is failed if the toppling-time is more than 1 sec.

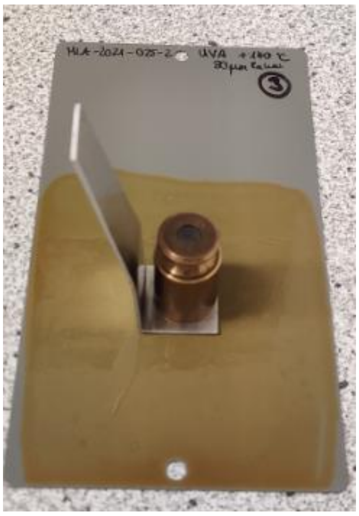

Figure S40. Dryness test example with an "L" shape metal piece placed in the middle of the panel containing the coating.

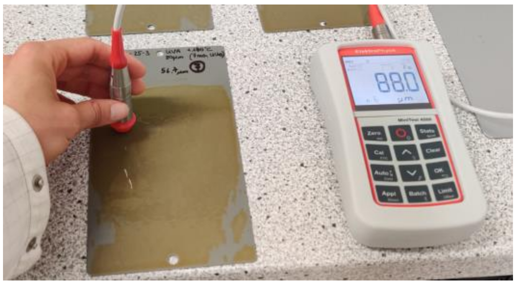

Figure S41. Thickness measurement of a dry coating.

Thickness measurements

The thickness is measured in five different point of the coating, using the film thickness monitor.

Gloss

The gloss of the coating is measured in three different areas with the use of a gloss reflectometer.

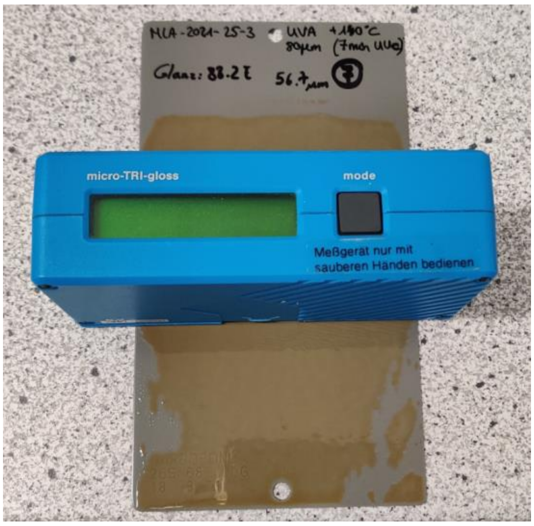

Figure S42. Reflectometer measuring the gloss of a coating sample.

Crosscut

The total thickness of the coating is calculated by addition of the e-coat thickness (25 microns) to the previously measured coating thickness. The choice of crosscut blade depends on the layer thickness. After the surface is cleaned with a brush two cuts are made in a cross shape. A tape is applied to the cut surface. After removal of the tape the damage is evaluated according to the classification (Figure 43, Class 1-5).

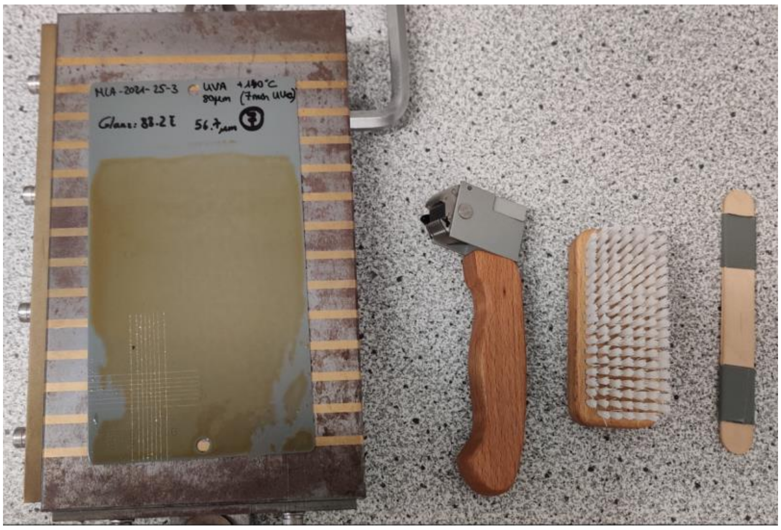

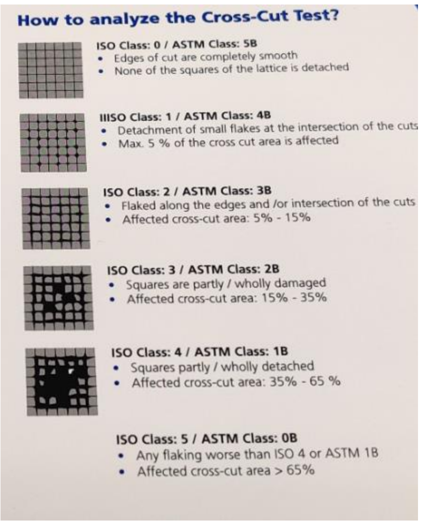

Figure S43. LEFT: Instrumentation for cross-cut test. RIGHT: Classification of cross-cut result tests.

Buchholz indentation hardness

The indenter is placed on the panel for 30 sec. Once removed. the lengths of the cut should be measured within another 30 sec.

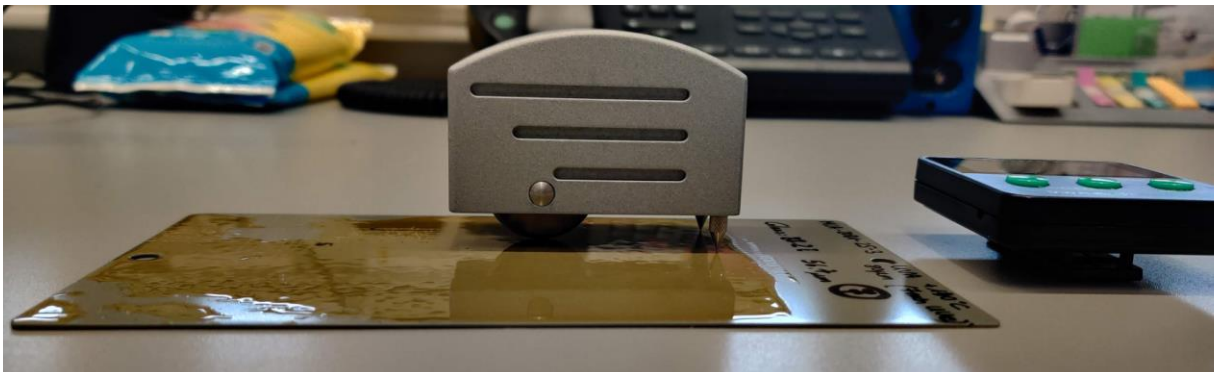

Figure S44. Buchholz hardness indenter placed on the surface of a panel to perform a hardness test.

MEK test

The solvent resistance is determined by the use of a special MEK hammer. The instrument is soaked in MEK and rubbed on the surface of the coating panel. After each 25 double rubs the coating is checked for defects. The procedure is repeated until a damage is detected or to a maximum of 200 double rubs. The solvent resistance is measured in the number of double rubs.

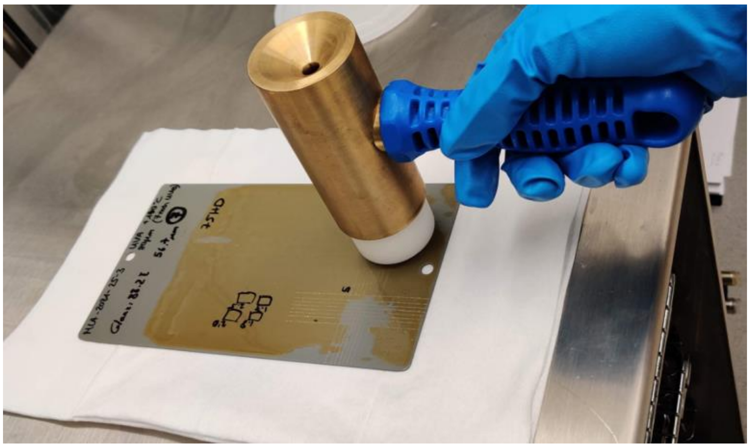

Figure S45. MEK hammer used for the solvent resistant test.

S56. NMR Spectra

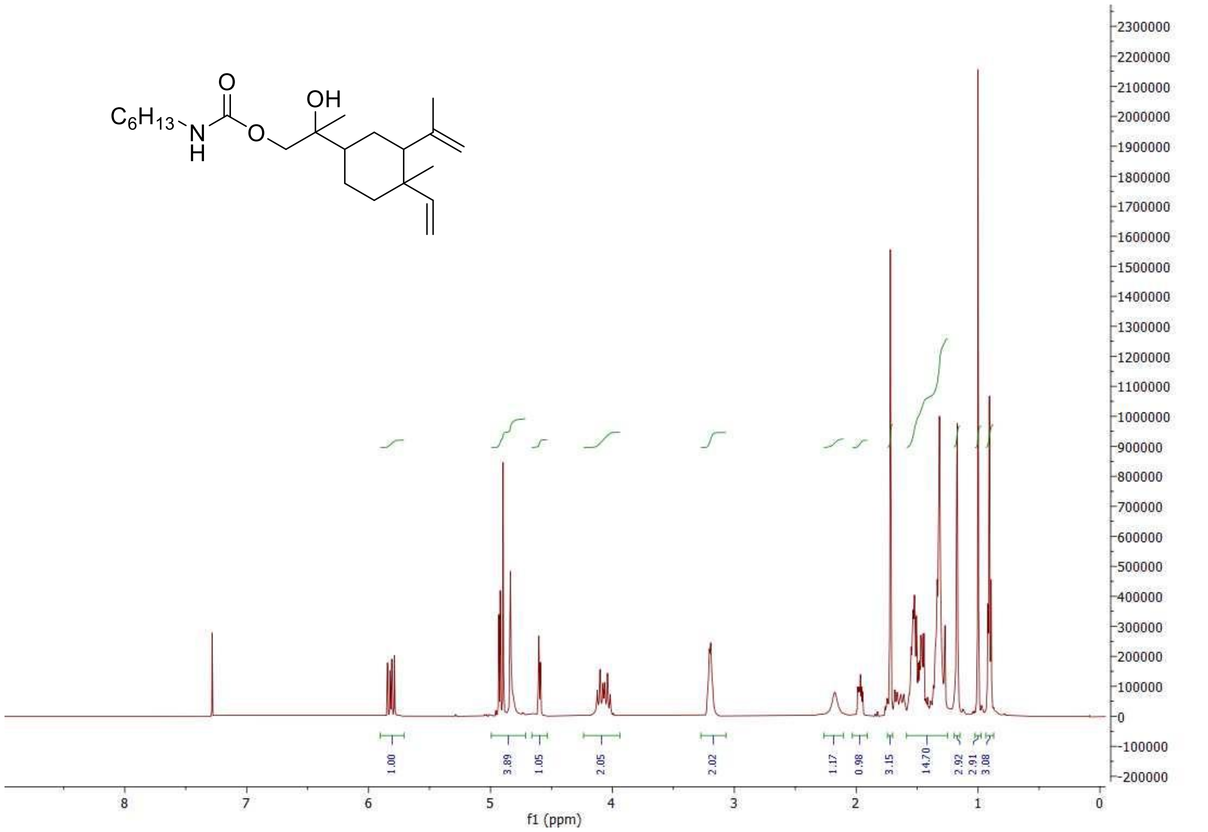

Figure S46. ^{1}H NMR ($CDCl_3$, 500 MHz) of **BECU1**.

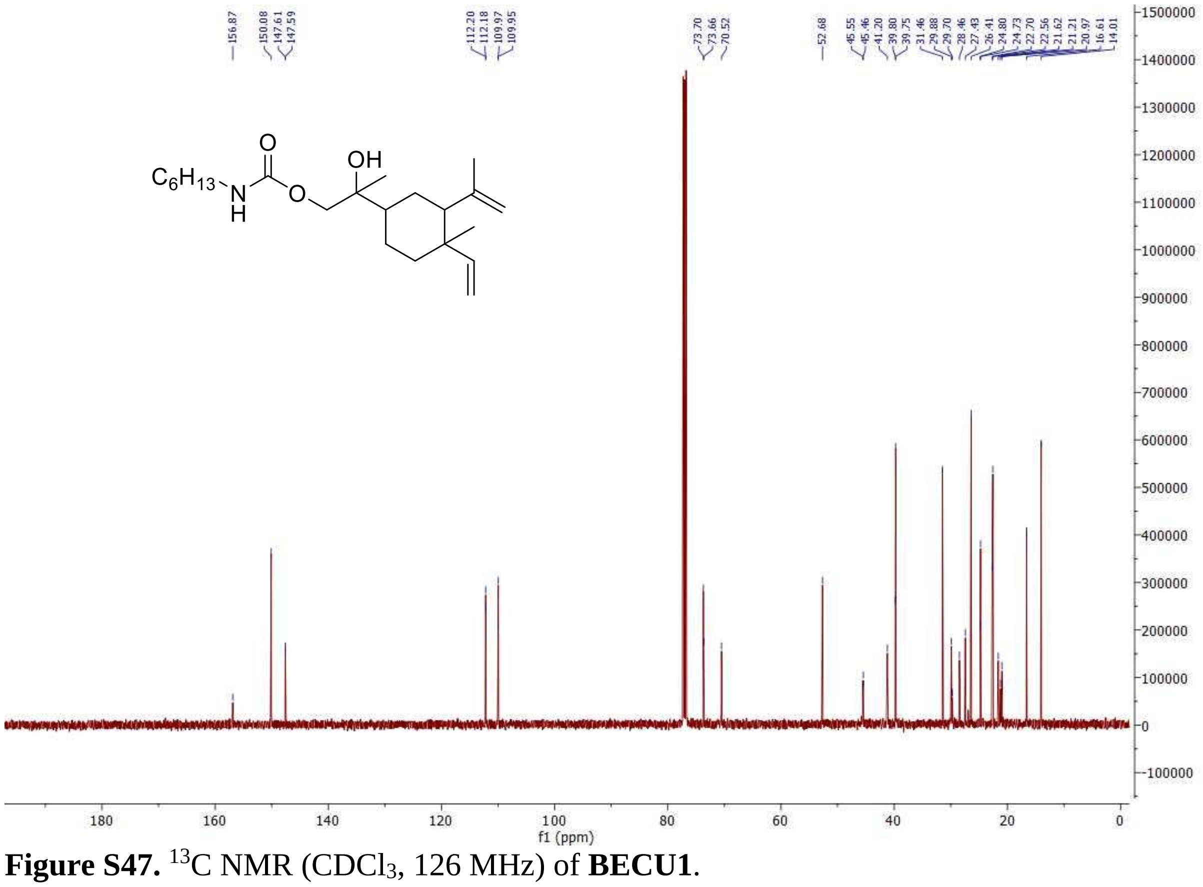

Figure S47. ^{13}C NMR ($CDCl_3$, 126 MHz) of **BECU1**.

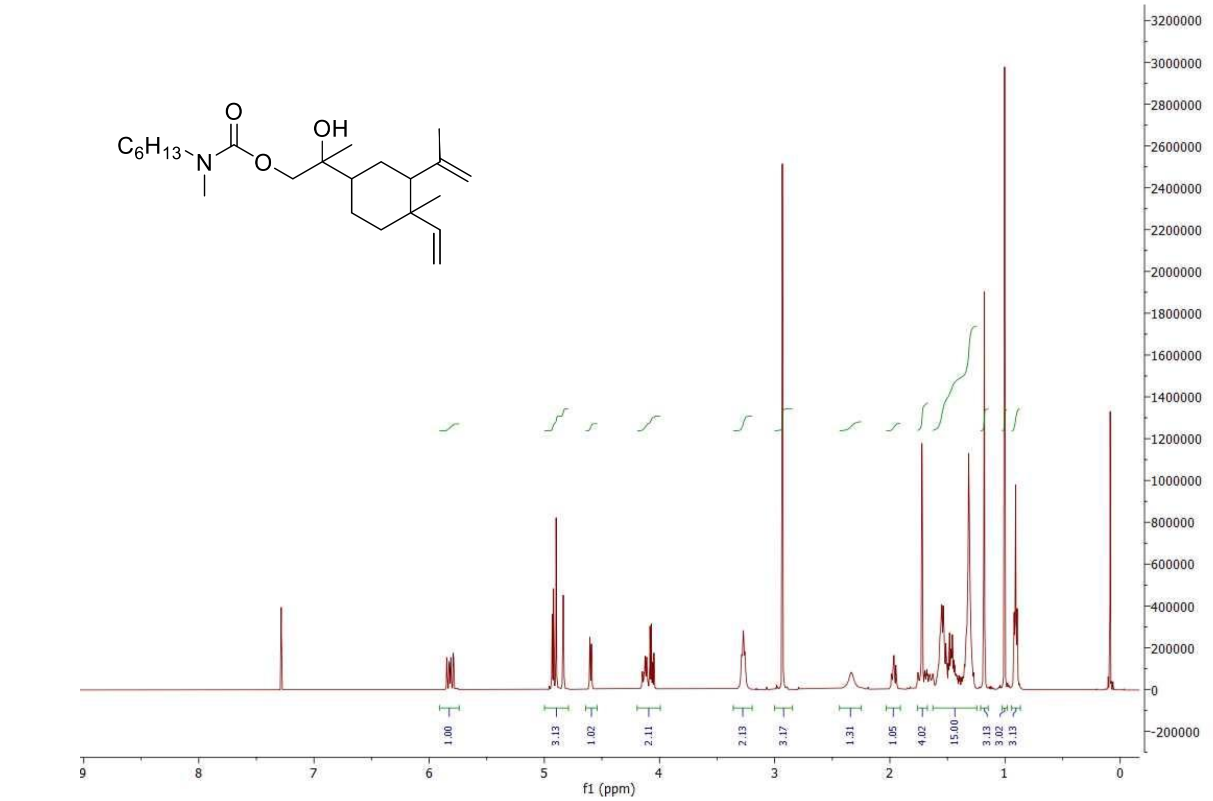

Figure S48. ^{1}H NMR ($CDCl_3$, 500 MHz) **BECU2**.

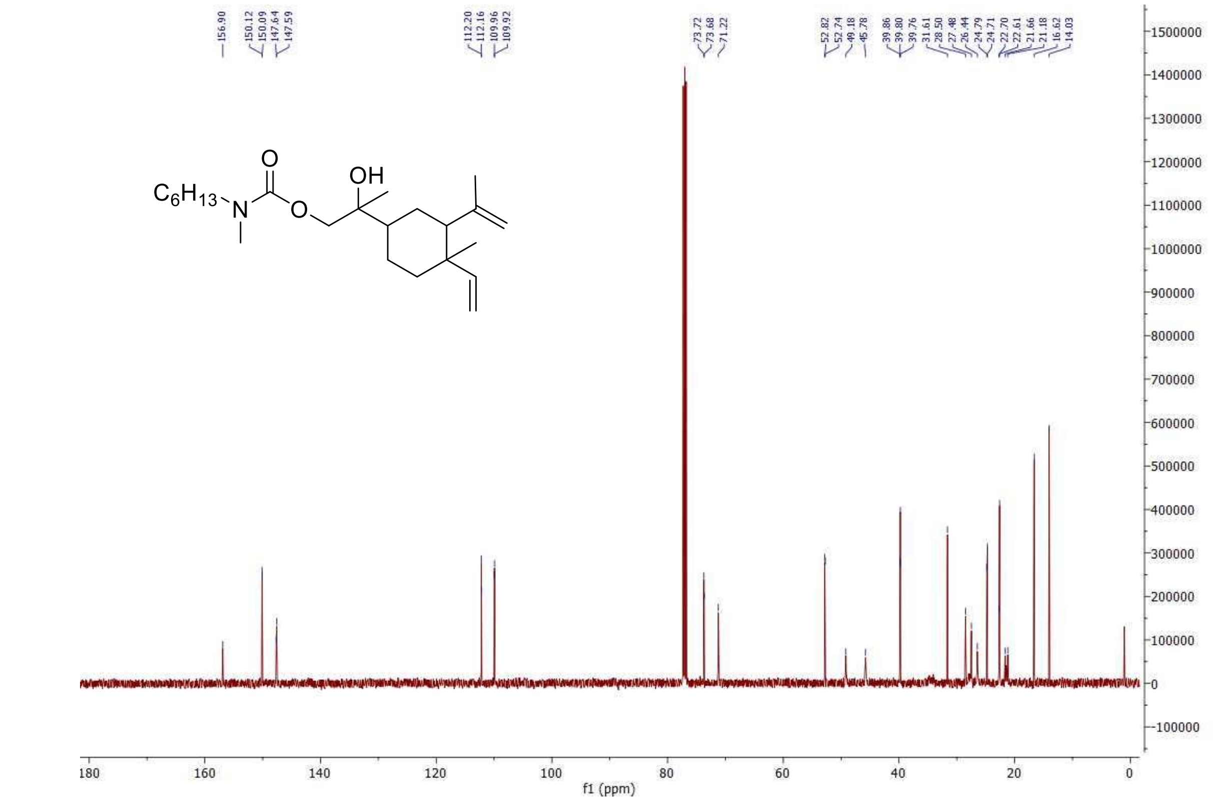

Figure S49. ^{13}C NMR ($CDCl_3$, 126 MHz) of **BECU2**.

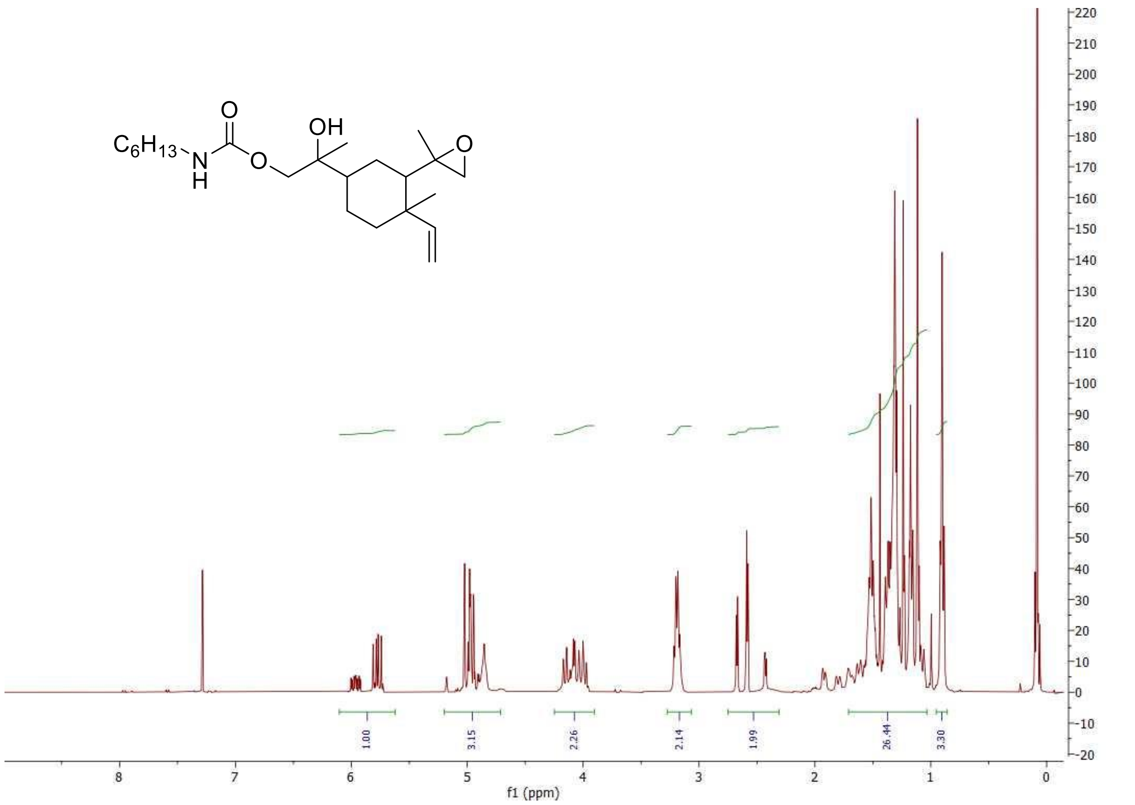

Figure S50. ^{1}H NMR ($CDCl_3$, 400 MHz) of **BEMCU1**.

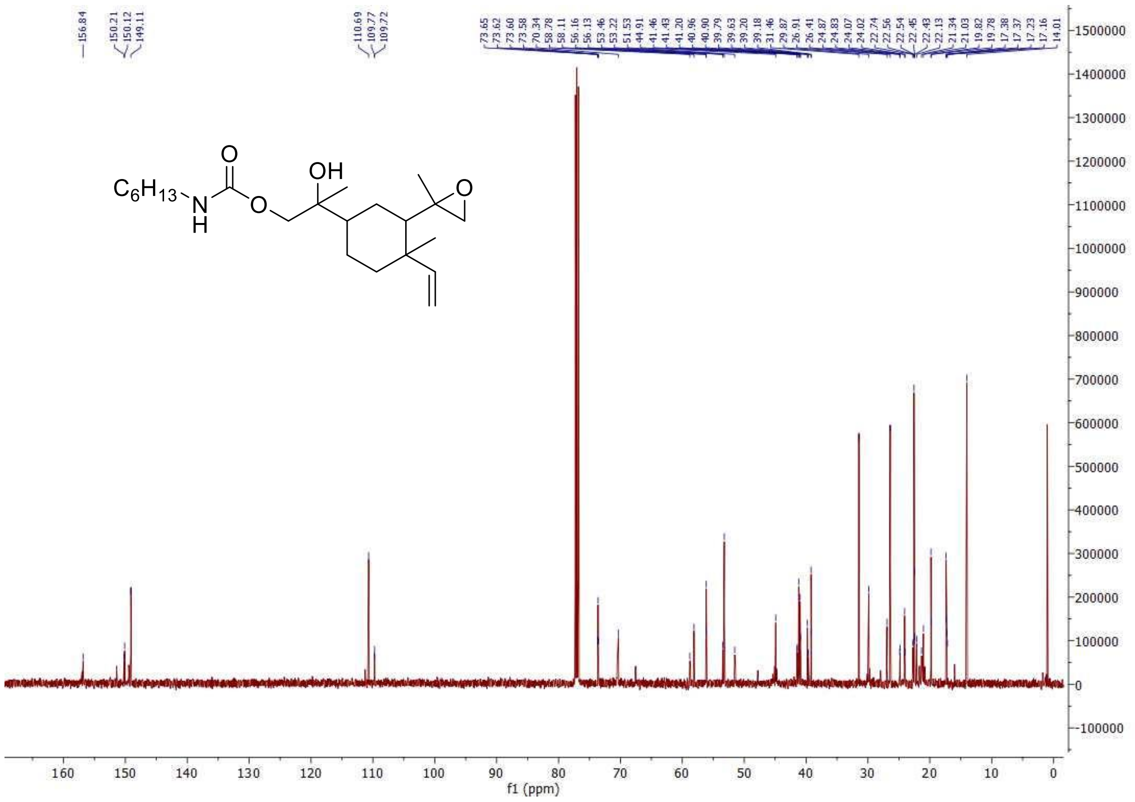

Figure S51. ^{13}C NMR ($CDCl_3$, 126 MHz) of **BEMCU1**.

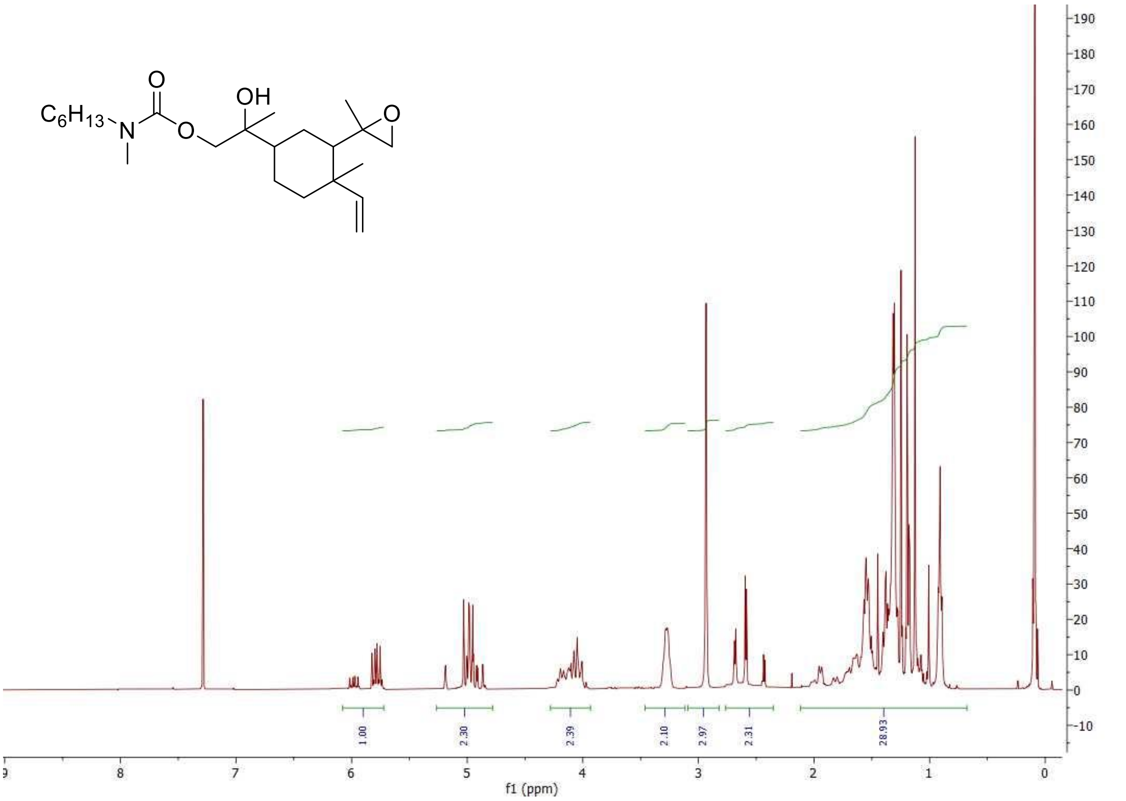

Figure S52. ^{1}H NMR ($CDCl_3$, 400 MHz) of **BEMCU2**.

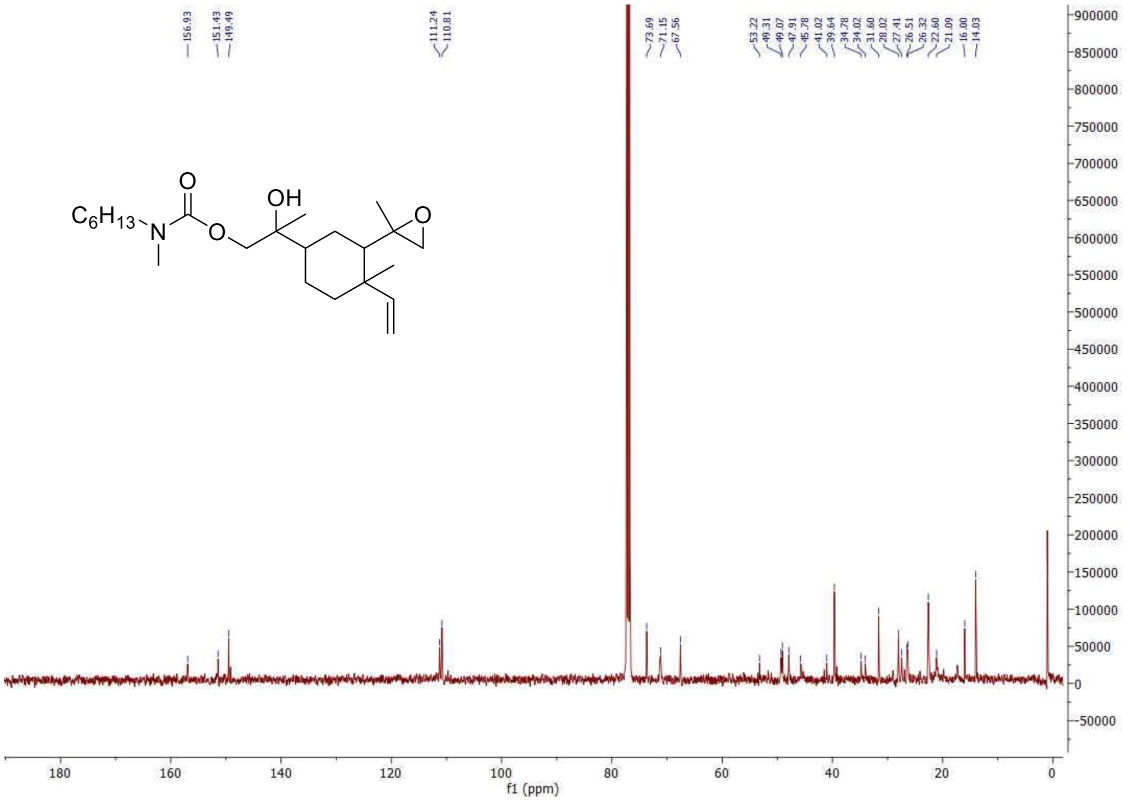

Figure S53. ^{13}C NMR ($CDCl_3$, 126 MHz) of **BEMCU2**.

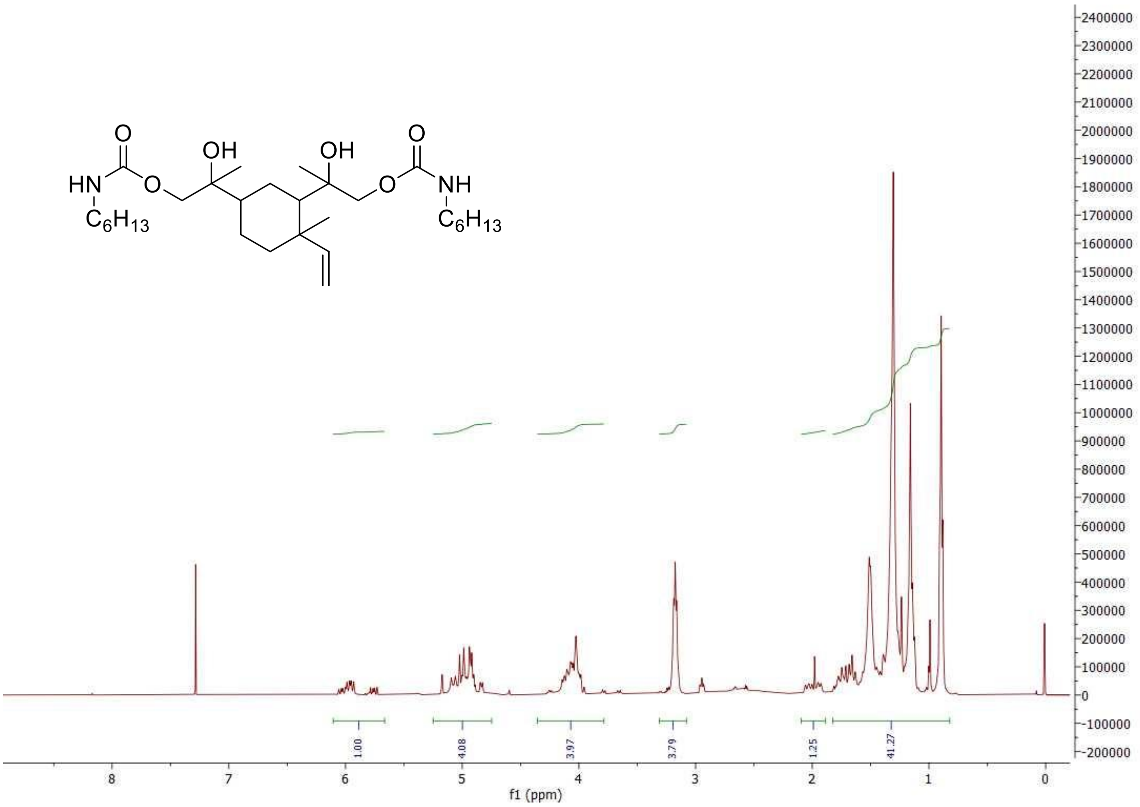

Figure S54. ^{1}H NMR ($CDCl_3$, 500 MHz) of **BEDCU1**.

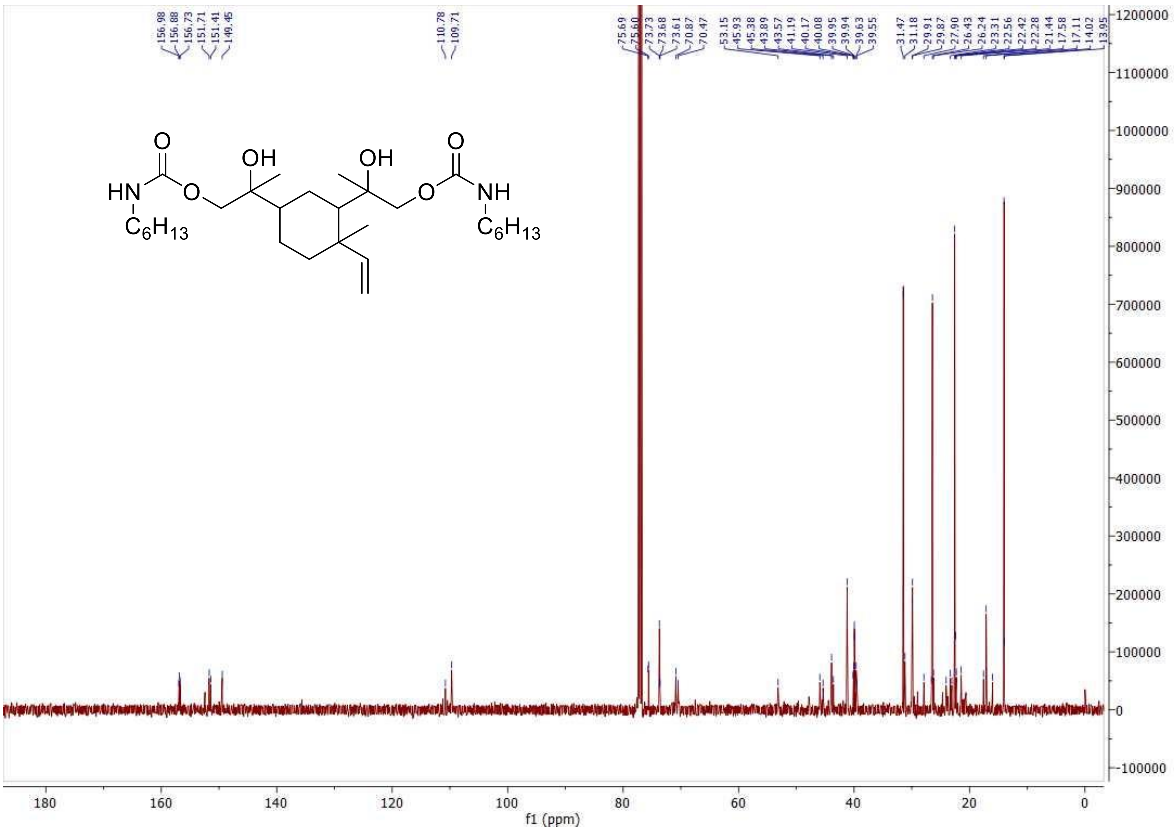

Figure S55. ^{13}C NMR ($CDCl_3$, 126 MHz) of **BEDCU1**.

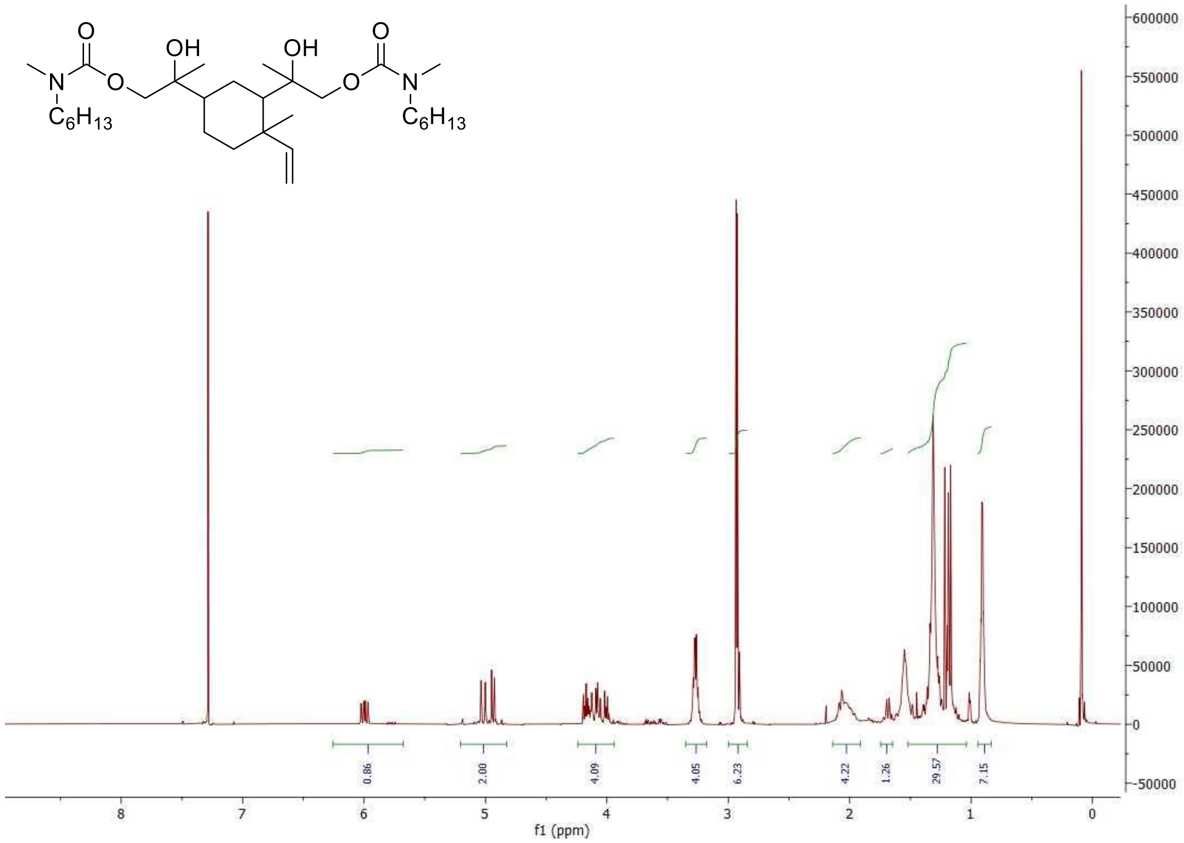

Figure S56. ^{1}H NMR ($CDCl_3$, 500 MHz) of **BEDCU2**.

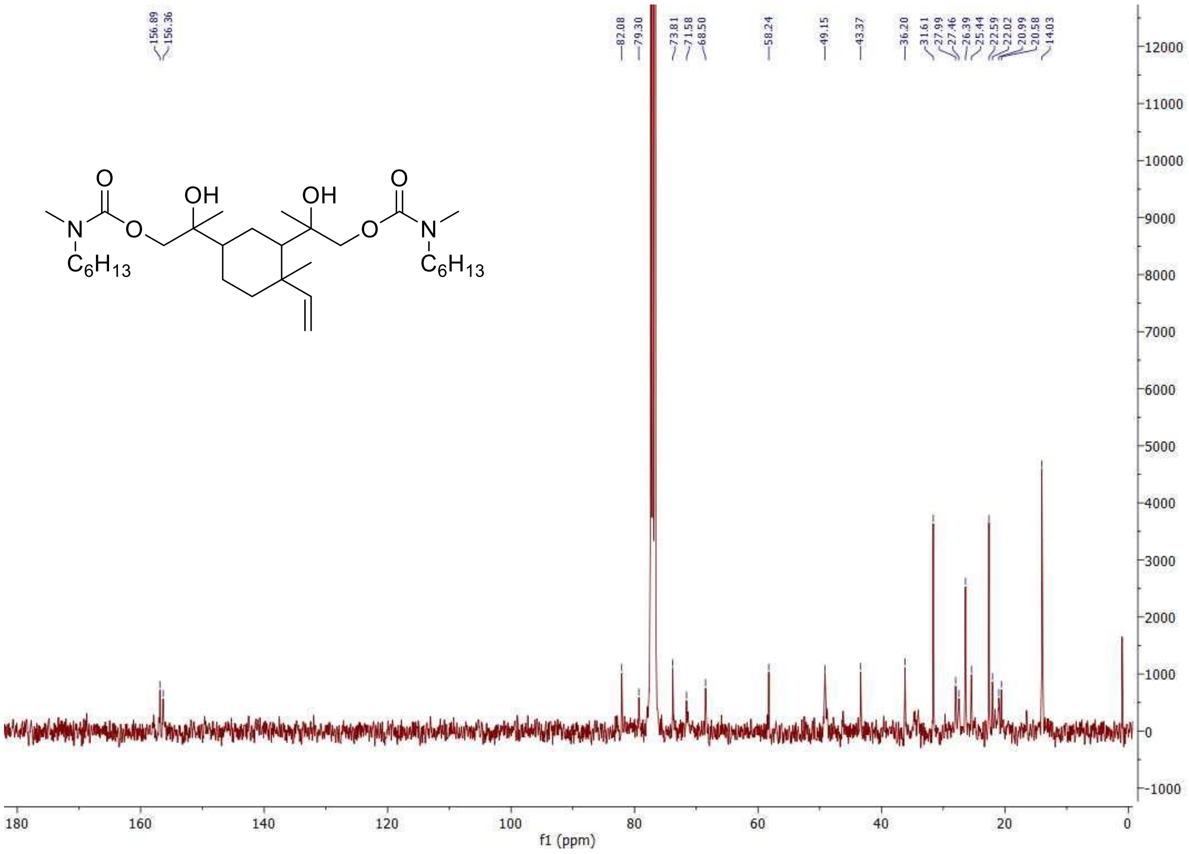

Figure S57. ^{13}C NMR ($CDCl_3$, 126 MHz) of **BEDCU2**.

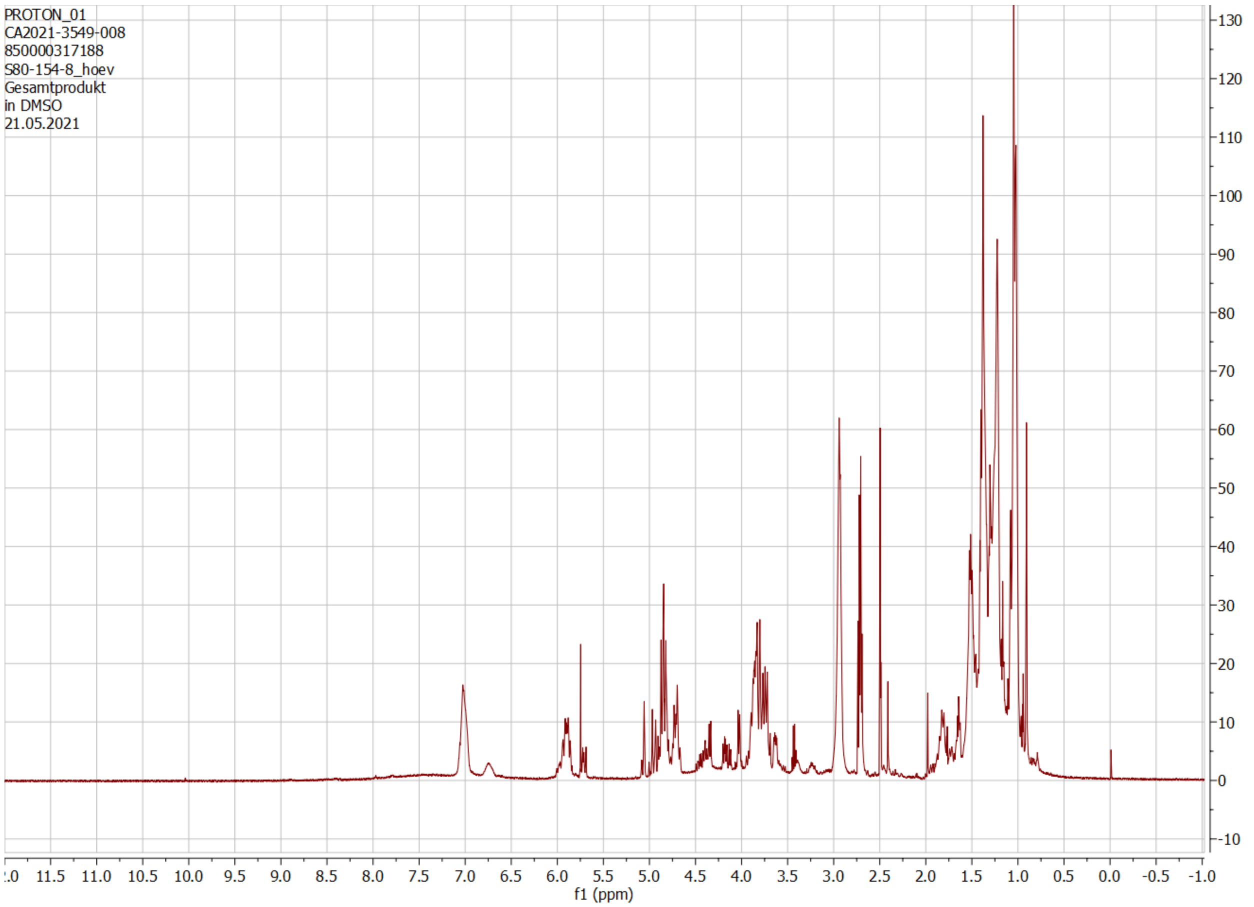

Figure S58. ^{1}H NMR (DMSO-d_6, 400 MHz) of **BEDCU3**.

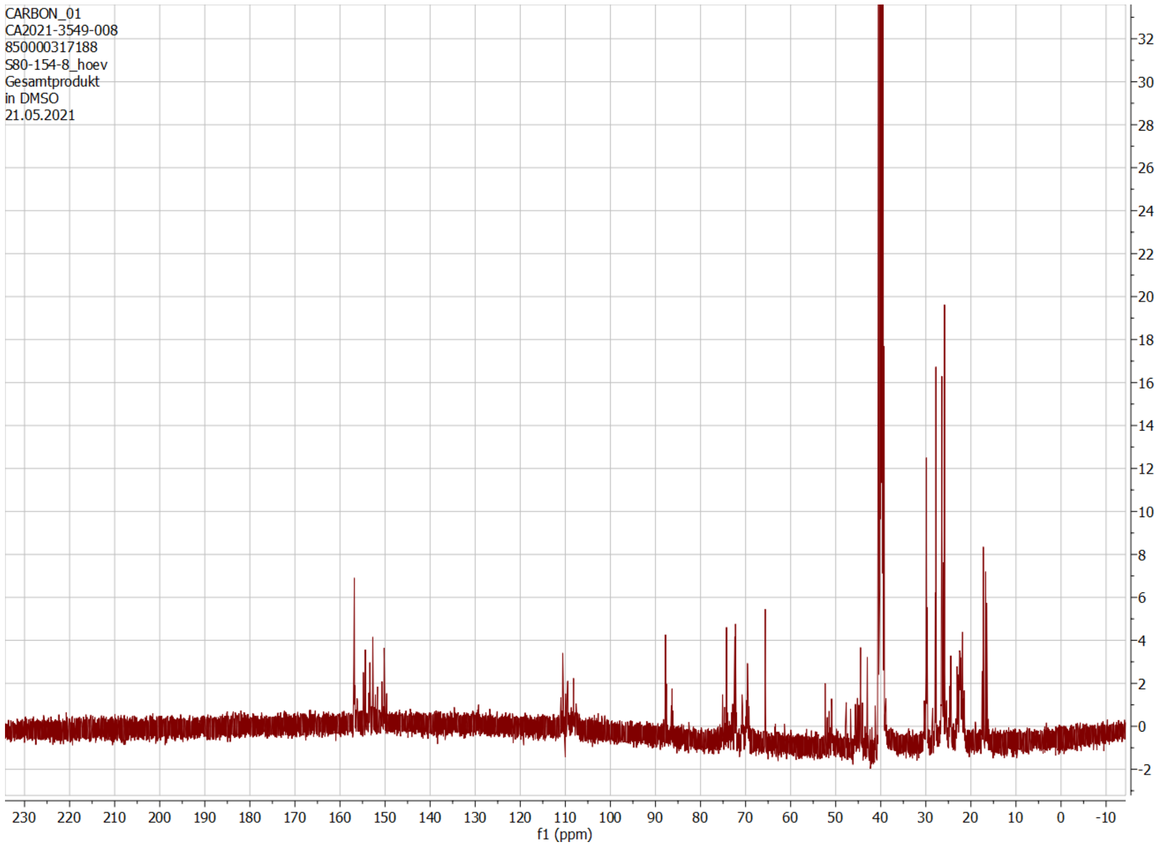

Figure S59. ^{13}C NMR (DMSO-d_6, 126 MHz) of **BEDCU3**.

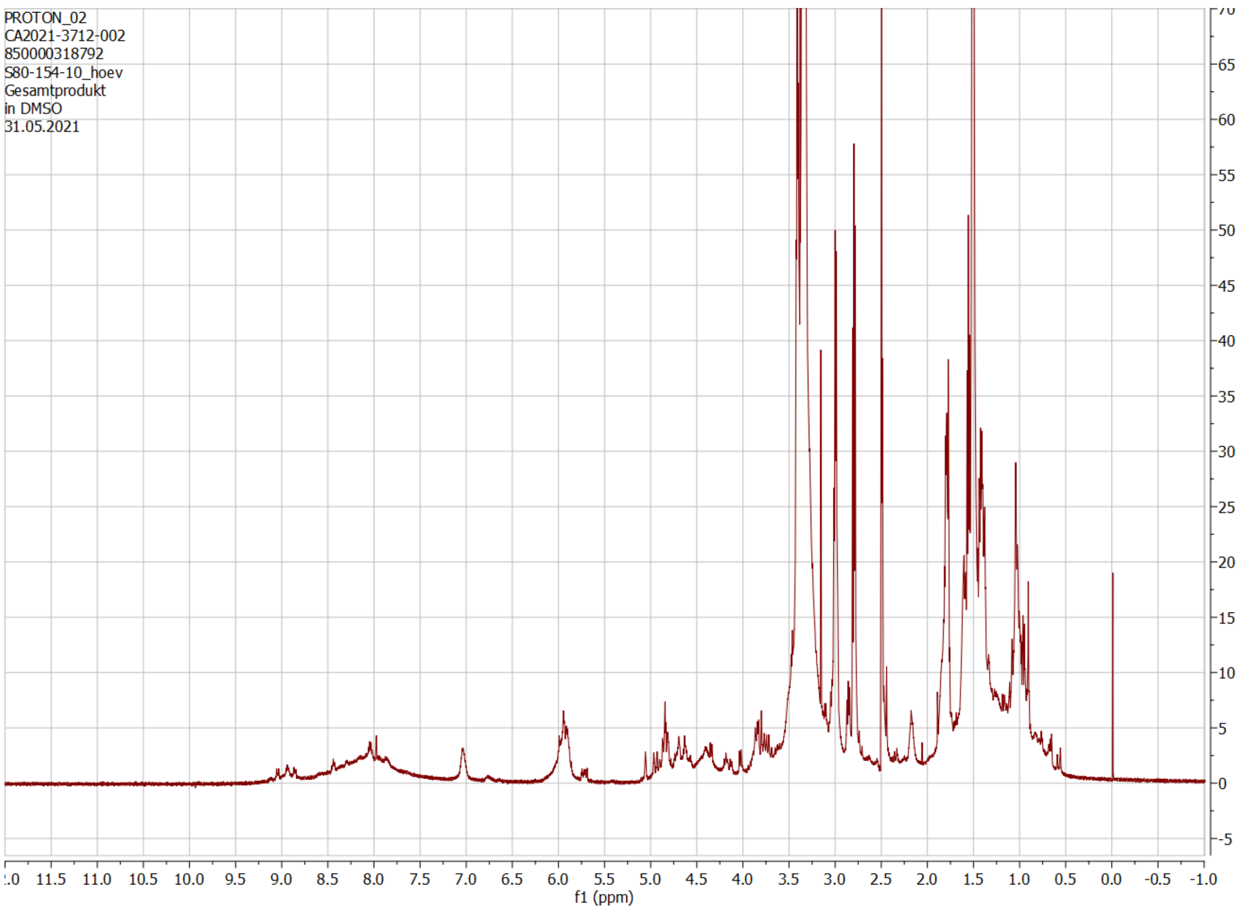

Figure S60. ^{1}H NMR (DMSO-d_6, 400 MHz) of **BEDCU4**.

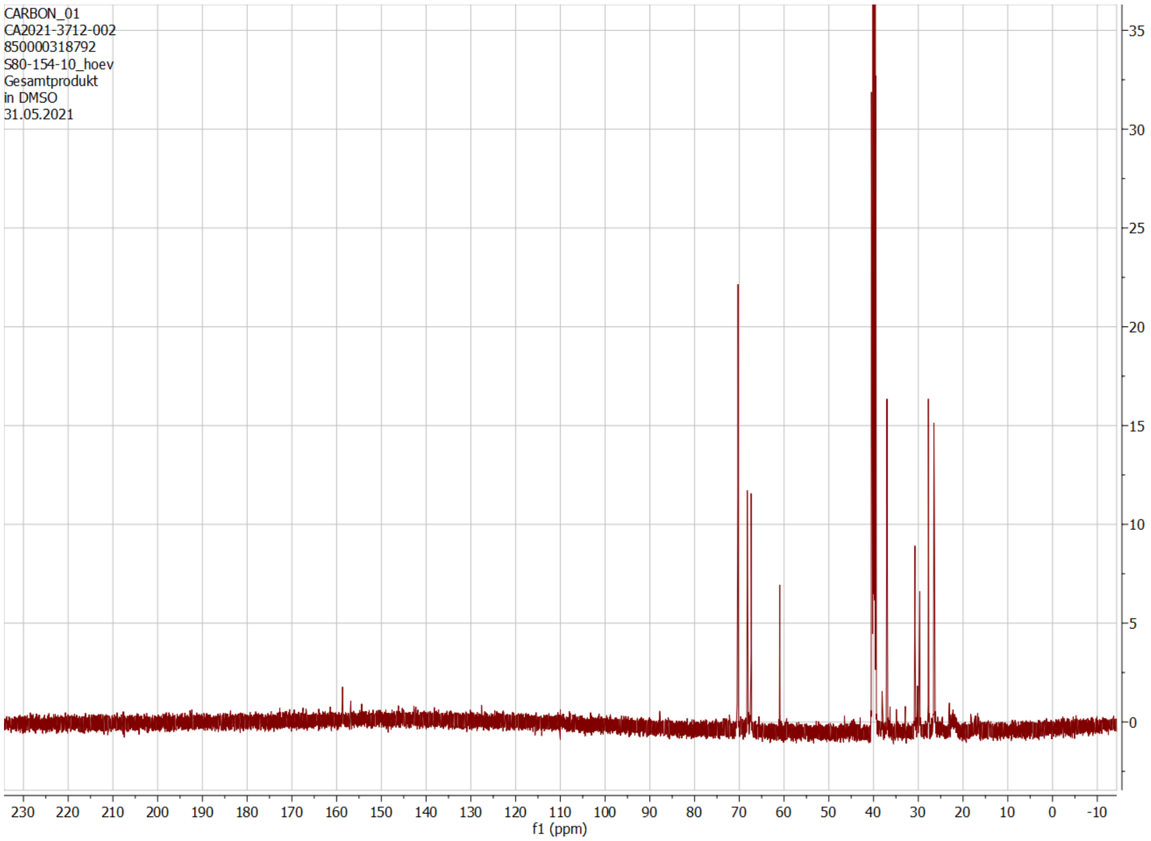

Figure S61. ^{13}C NMR (DMSO-d_6, 126 MHz) of **BEDCU4**.

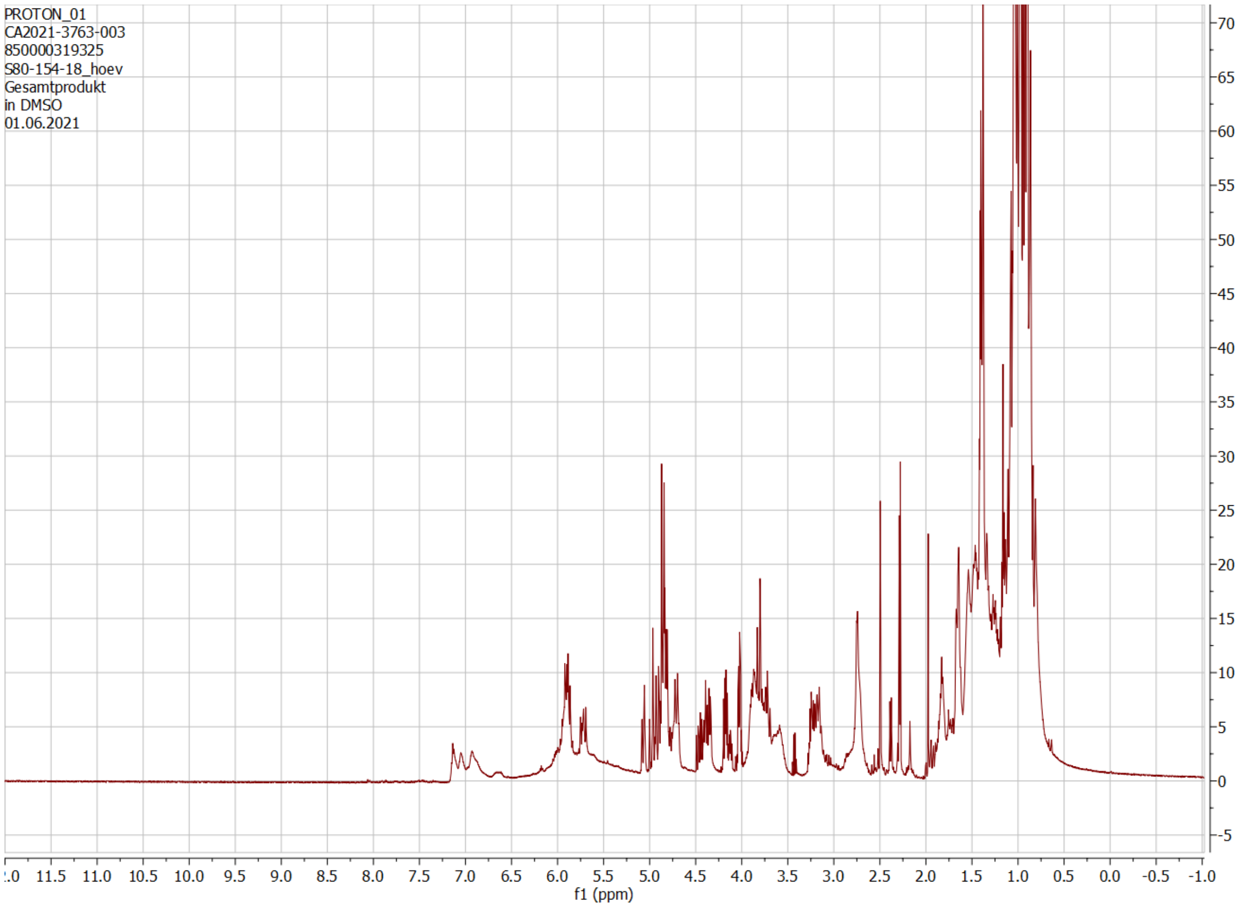

Figure S62. ^{1}H NMR (DMSO-d_6, 400 MHz) of **BEDCU5**.

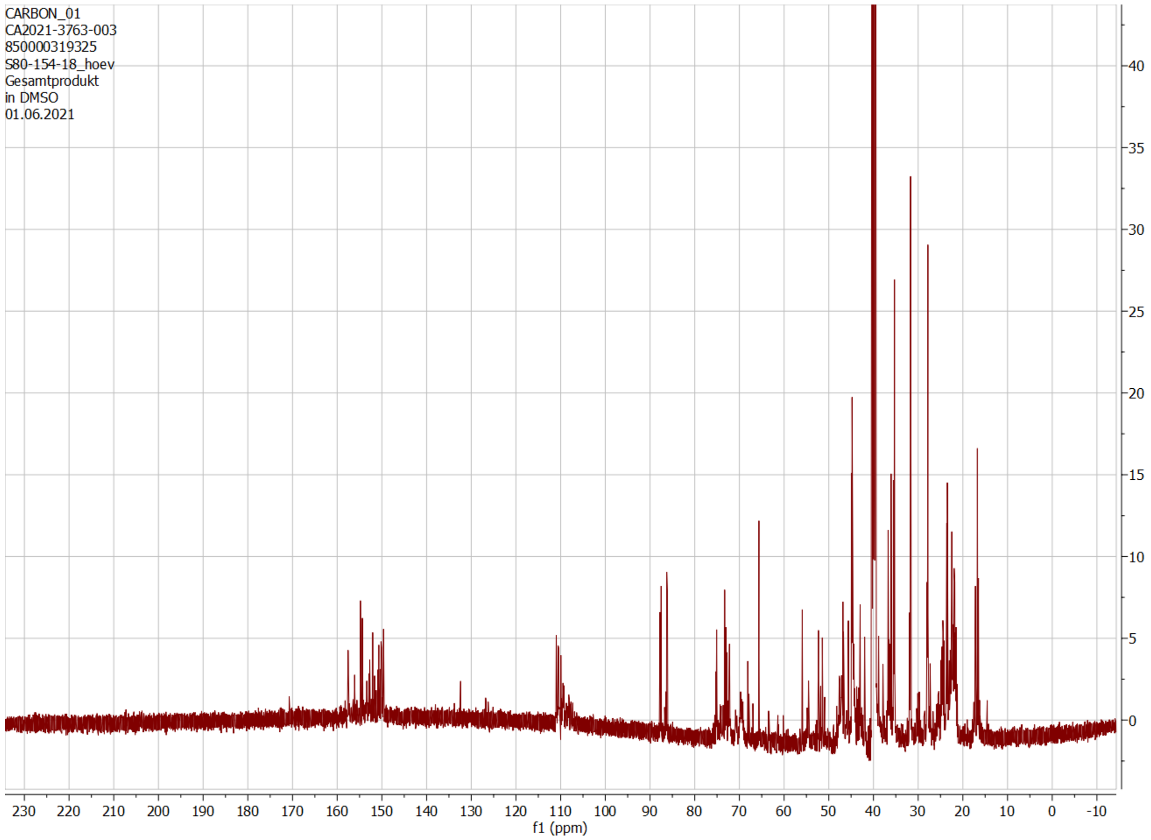

Figure S63. ^{13}C NMR (DMSO-d_6, 126 MHz) of **BEDCU5**.

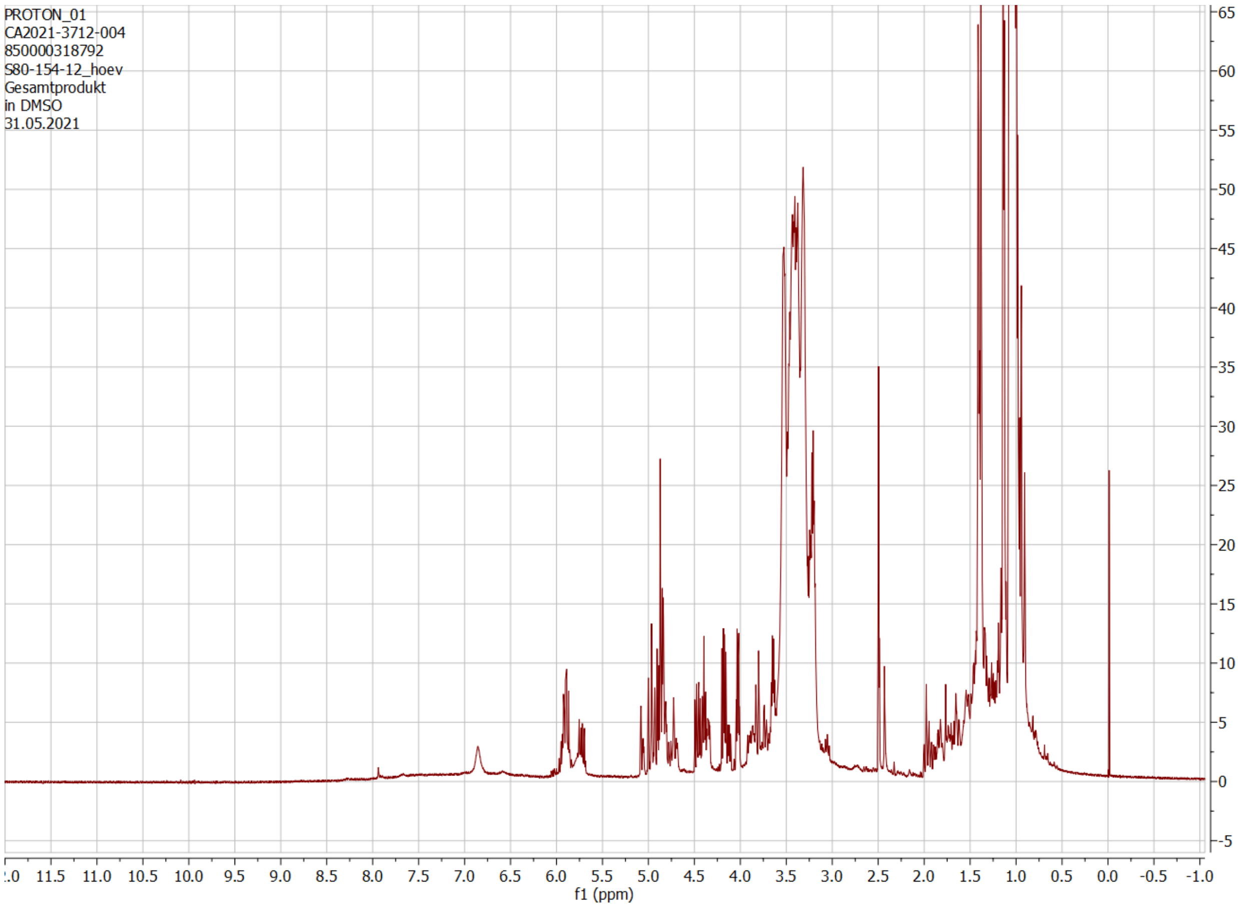

Figure S64. ^{1}H NMR (DMSO-d_6, 400 MHz) of **BEDCU6**.

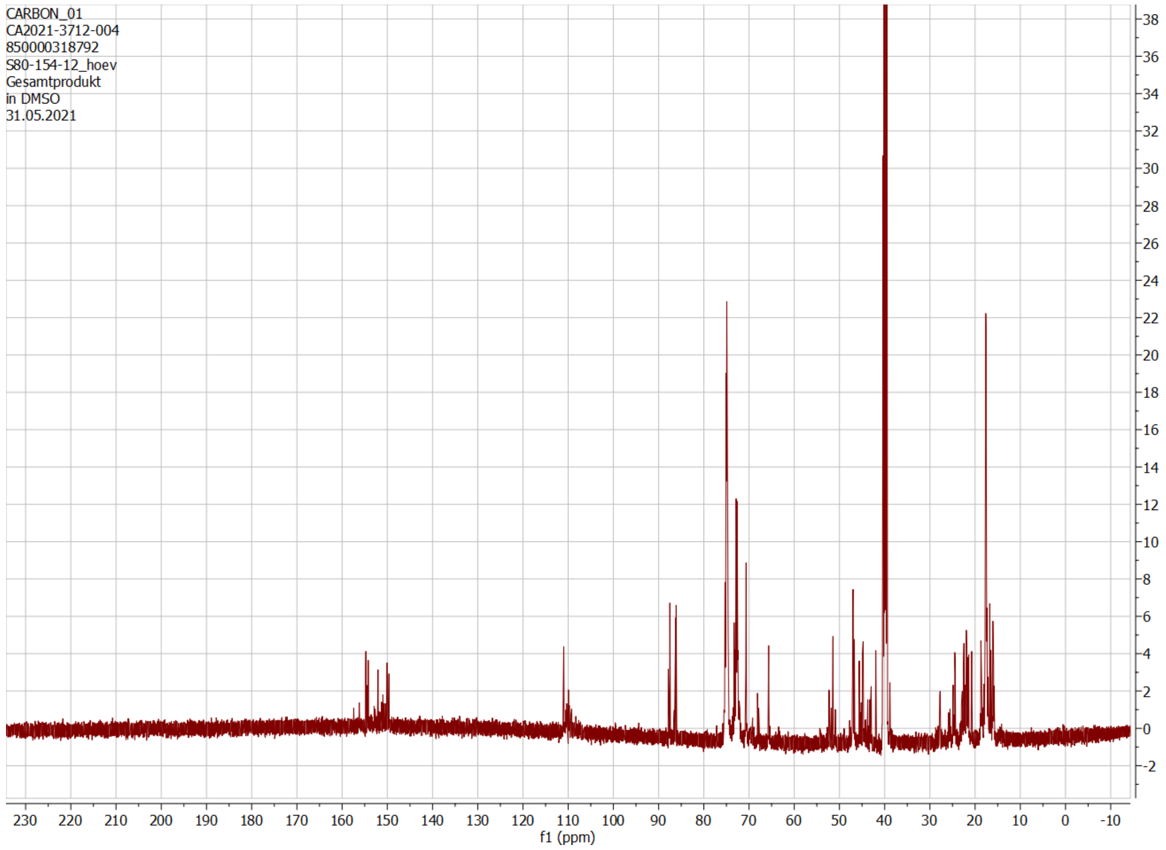

Figure S65. ^{13}C NMR (DMSO-d_6, 126 MHz) of **BEDCU6**.

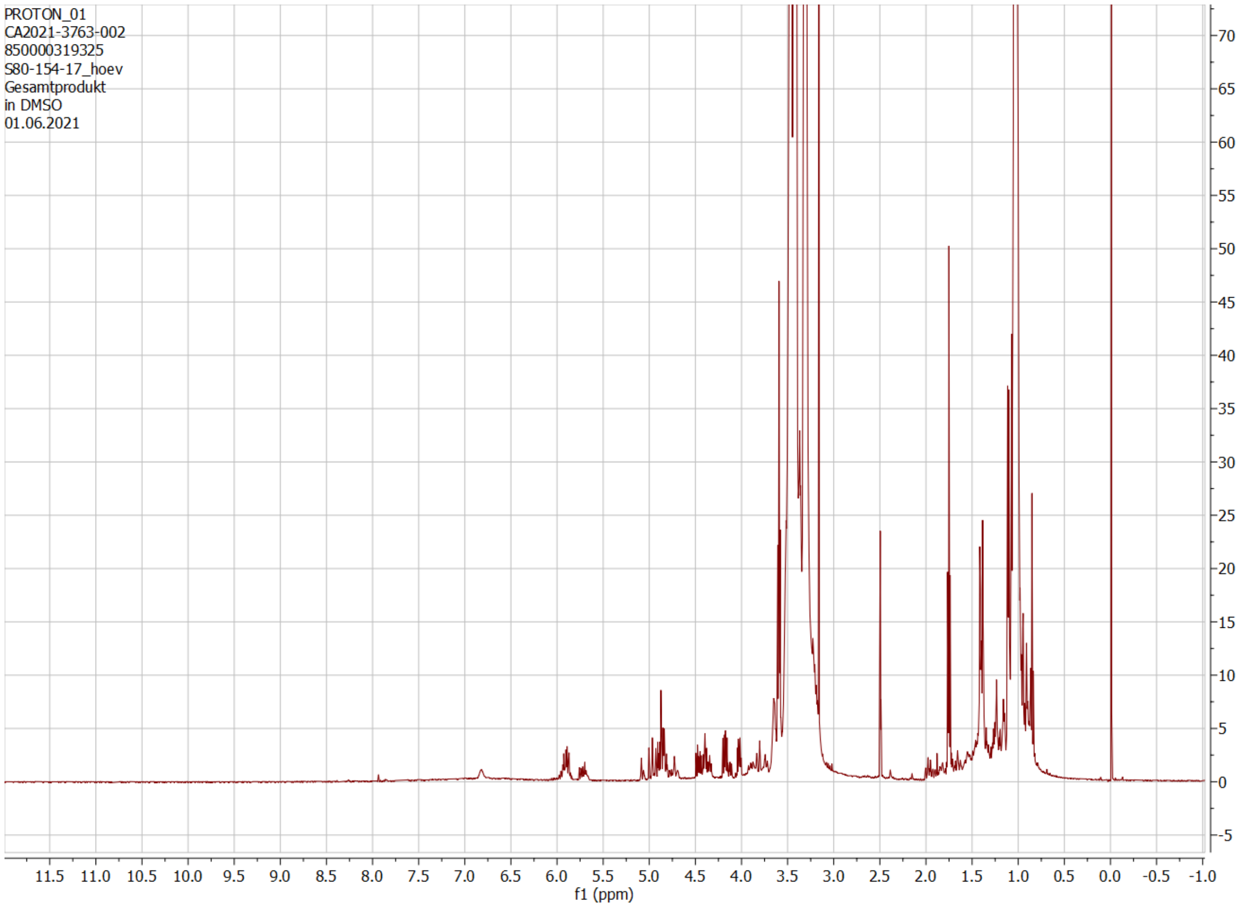

Figure S66. ^{1}H NMR (DMSO-d_6, 400 MHz) of **BEDCU7**.

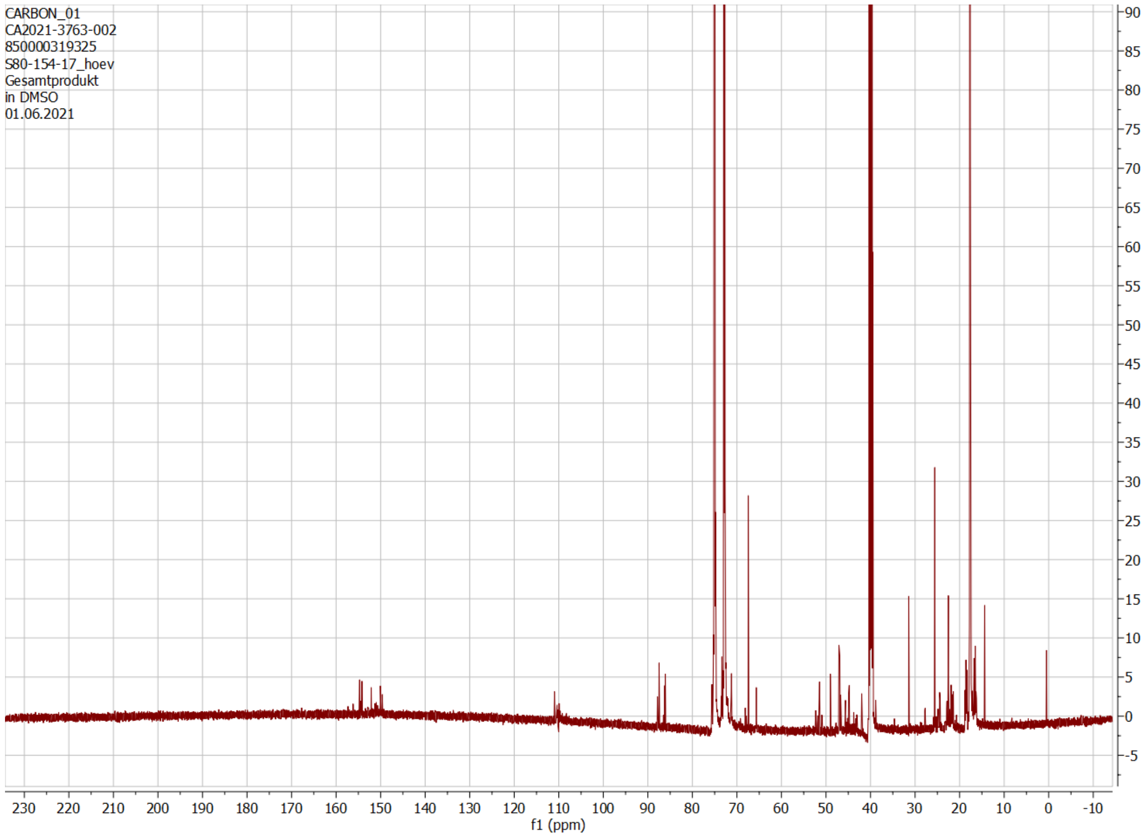

Figure S67. ^{13}C NMR (DMSO-d_6, 126 MHz) of **BEDCU7**.

S67. FT-IR spectra

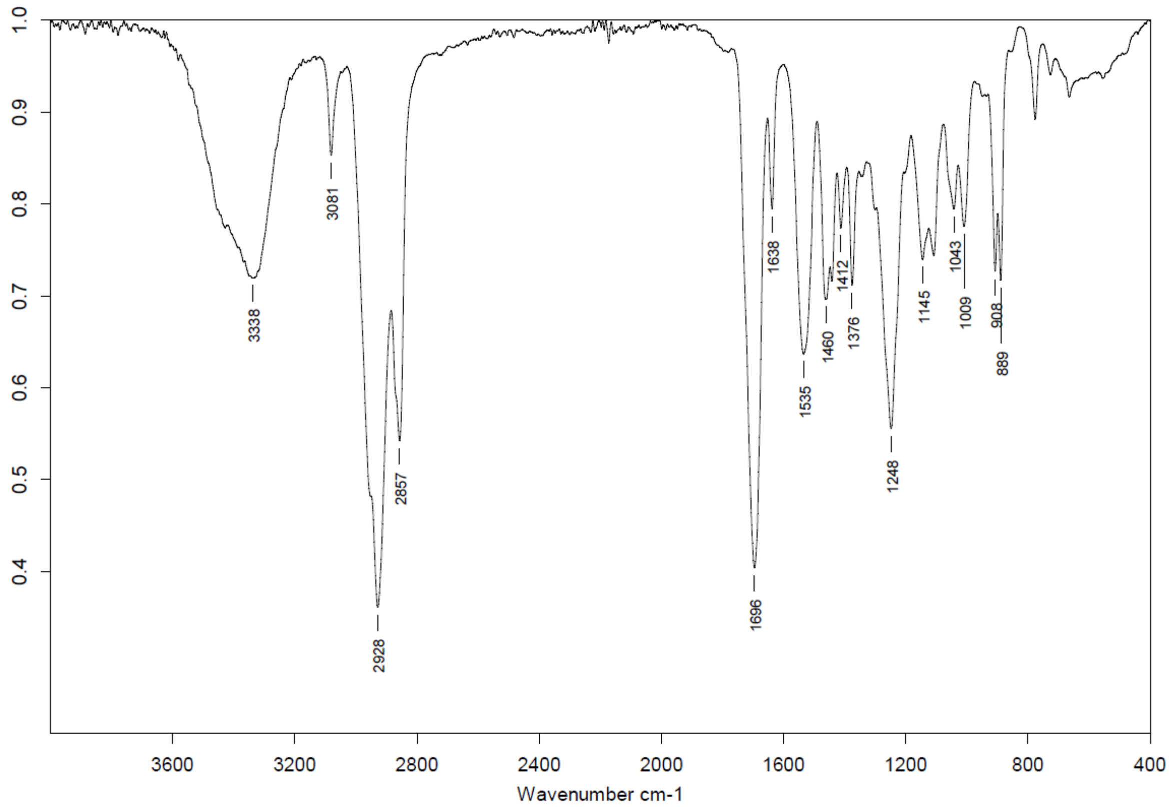

Figure S68. FT-IR spectra of **BECU1**.

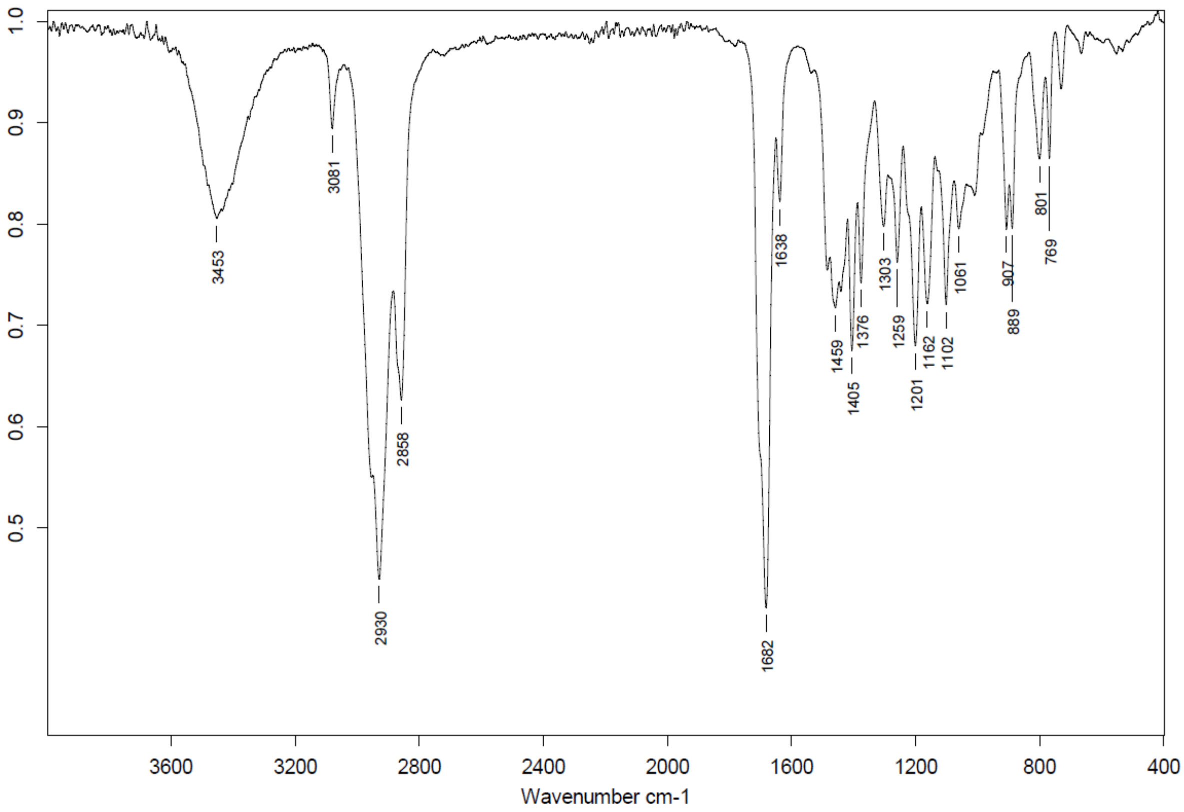

Figure S69. FT-IR spectra of **BECU2**.

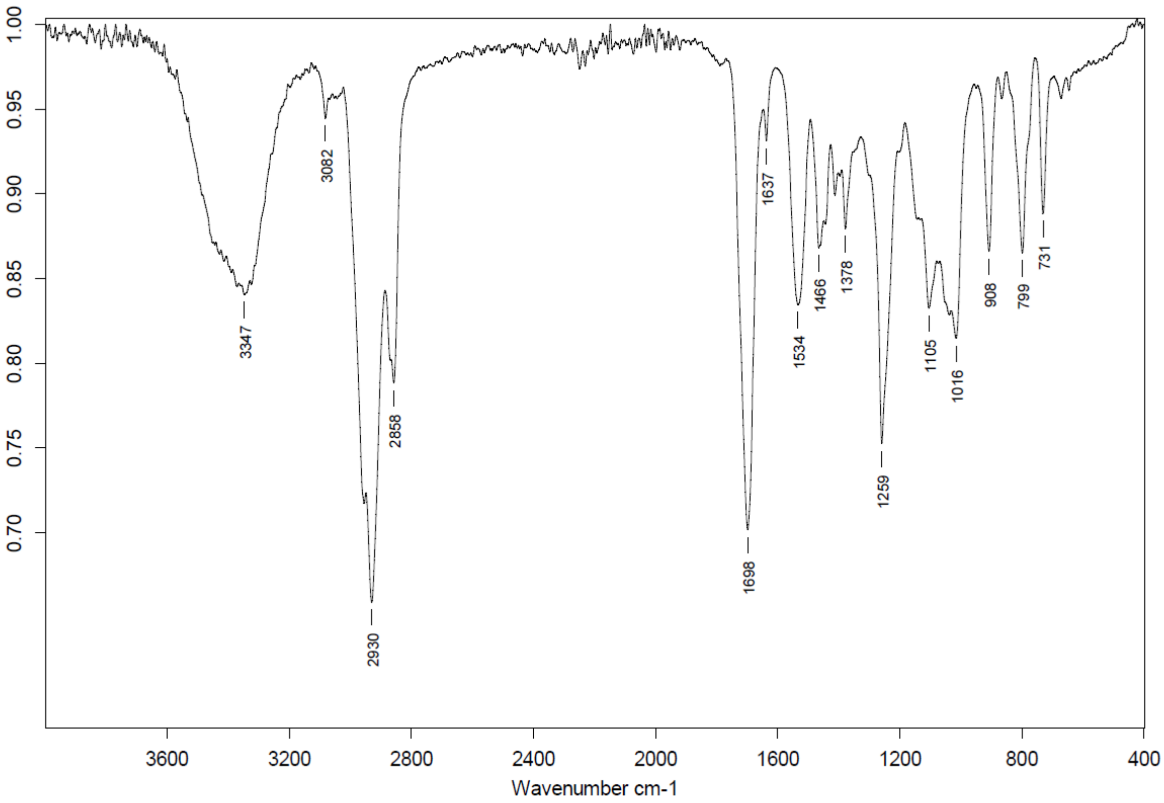

Figure S70. FT-IR spectra of **BEMCU1**.

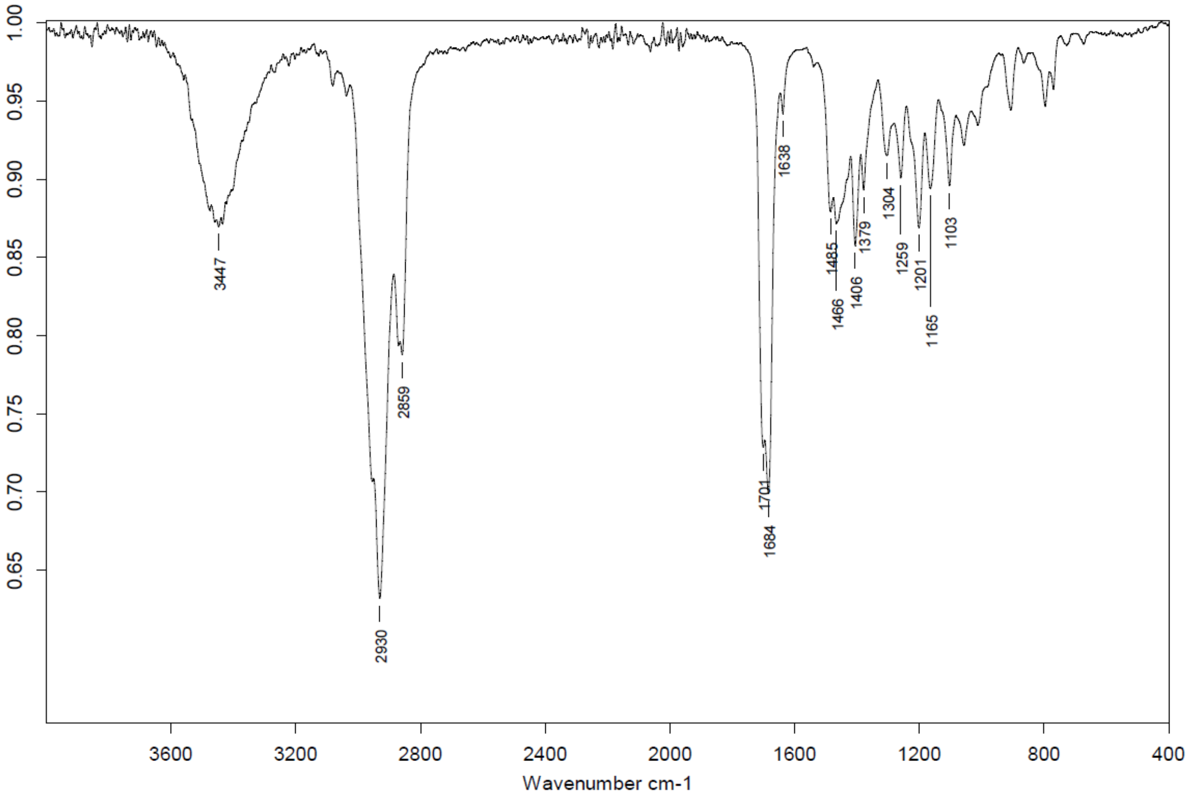

Figure S71. FT-IR spectra of **BEMCU2**.

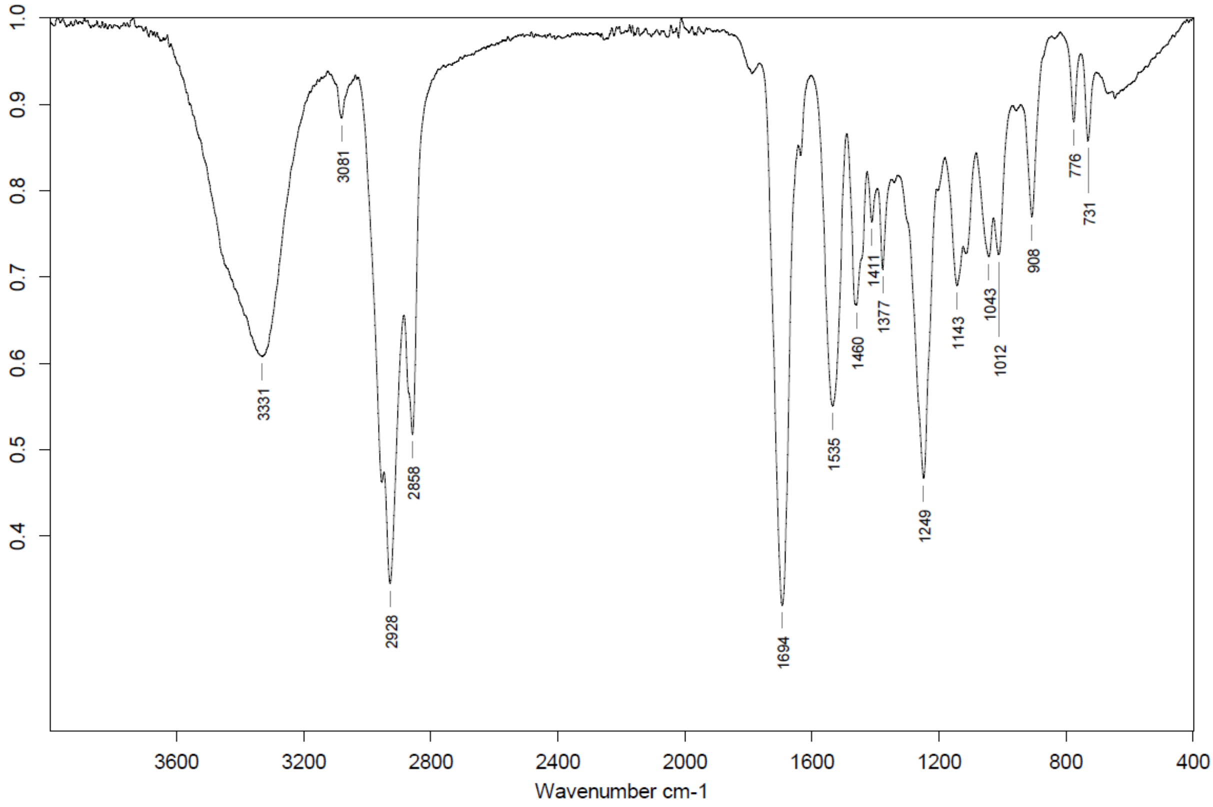

Figure S72. FT-IR spectra of **BEDCU1**.

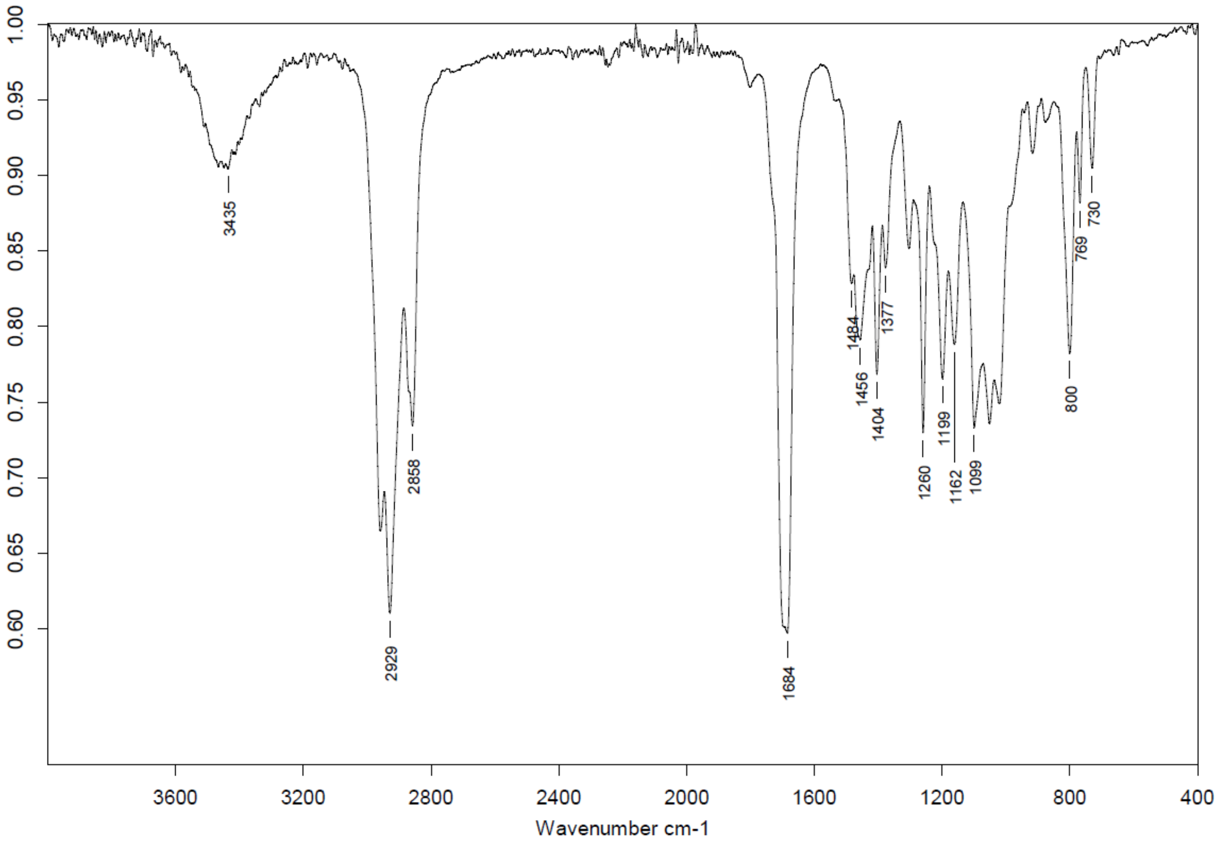

Figure S73. FT-IR spectra of **BEDCU2**.

S70. GPC analyses

Figure S74. GPC traces of **BEDCU3**.

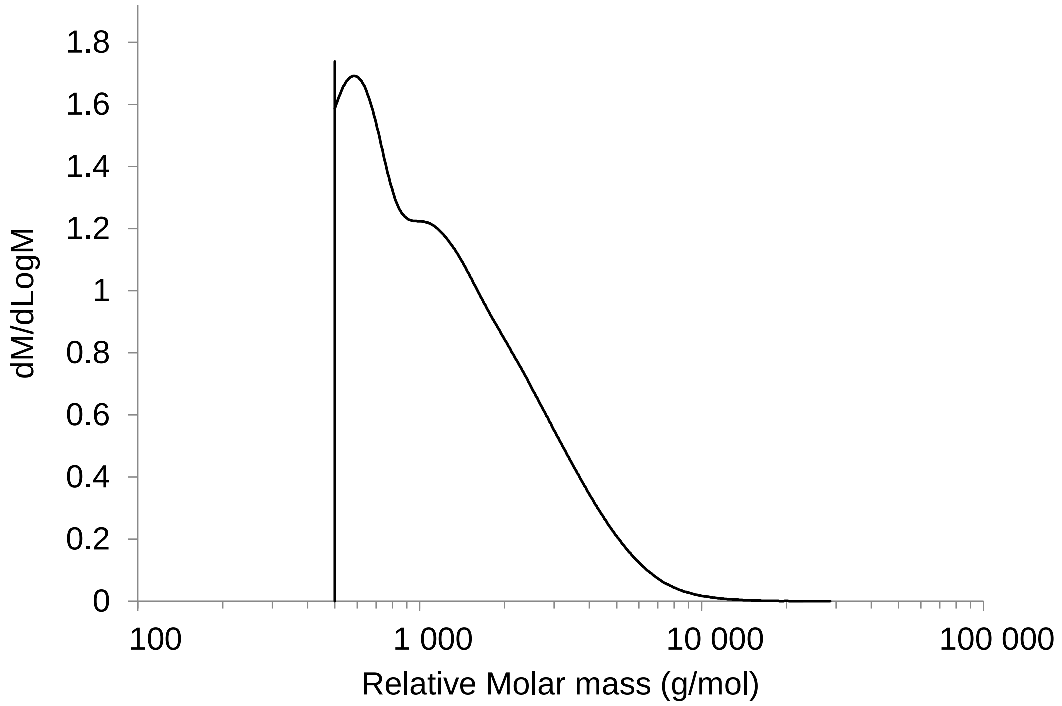

M_n [kDa]	*Đ*
1.6	1.5

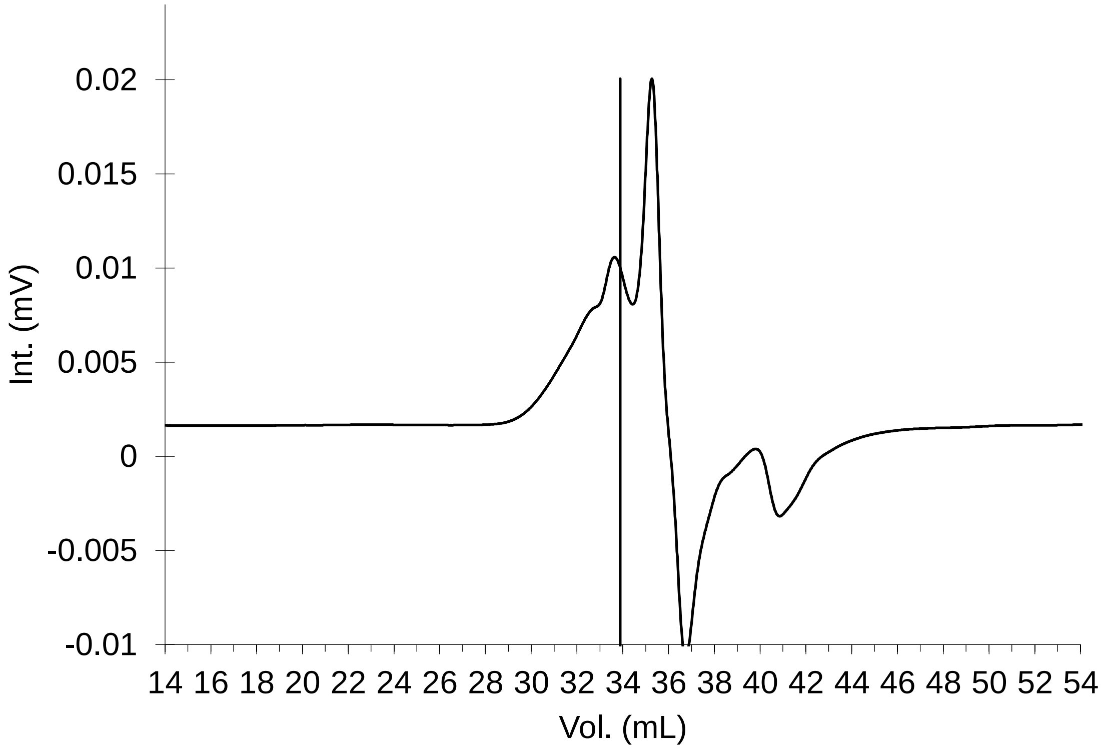

Figure S75. GPC traces of **BEDCU4**.

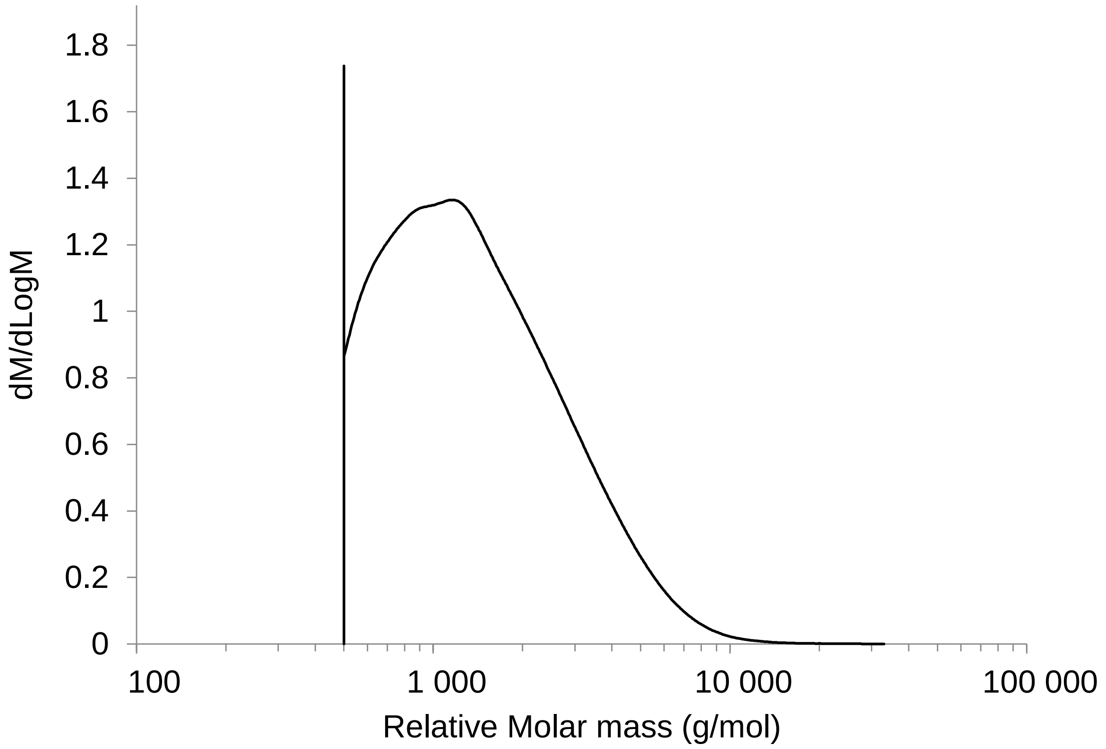

M_n [kDa]	*Đ*
1.3	1.3

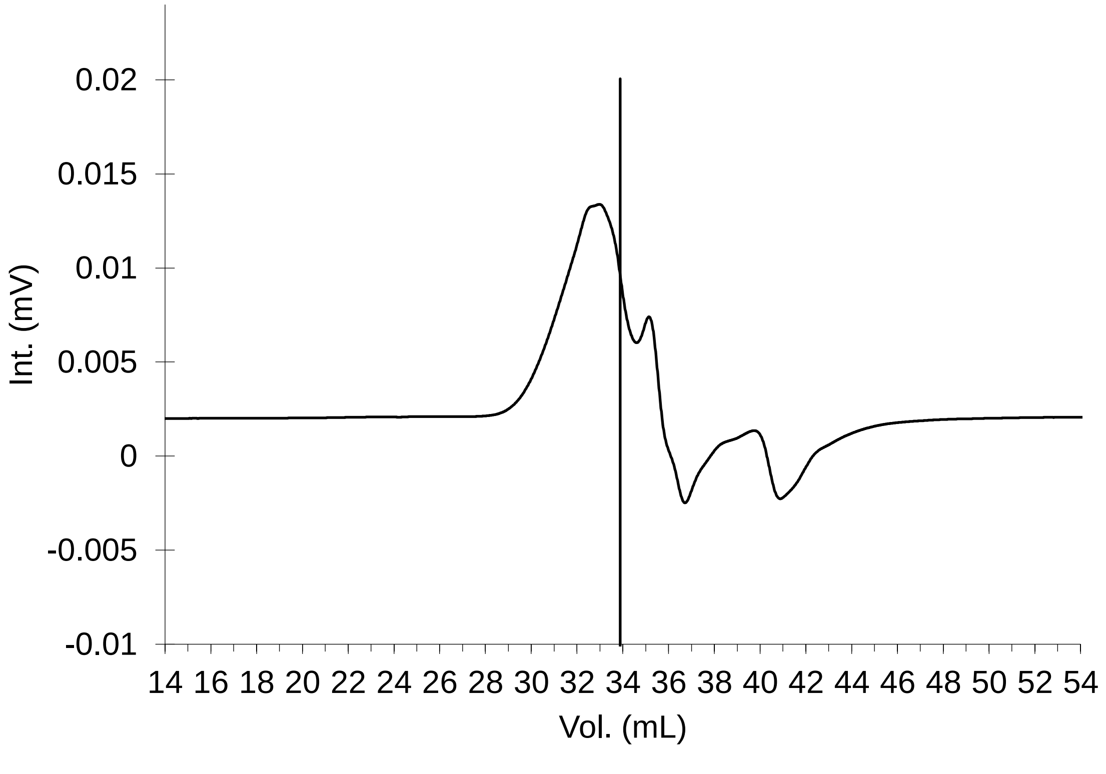

Figure S76. GPC traces of **BEDCU5**.

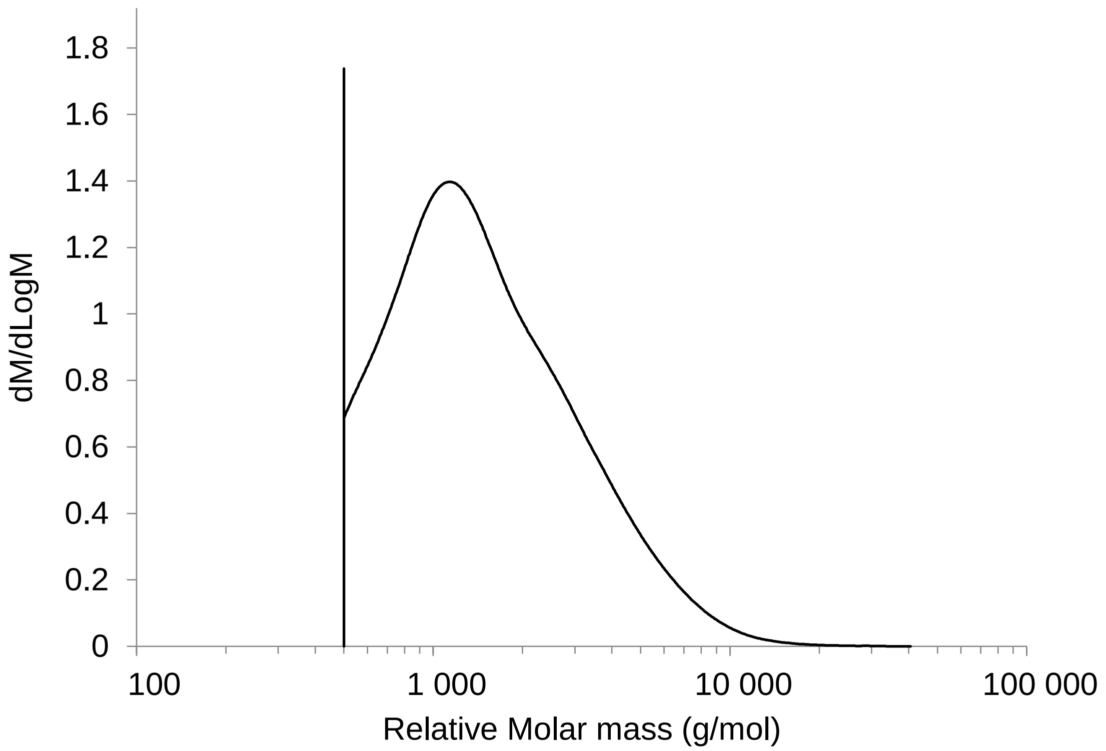

M_n [kDa]	*Đ*
1.8	1.5

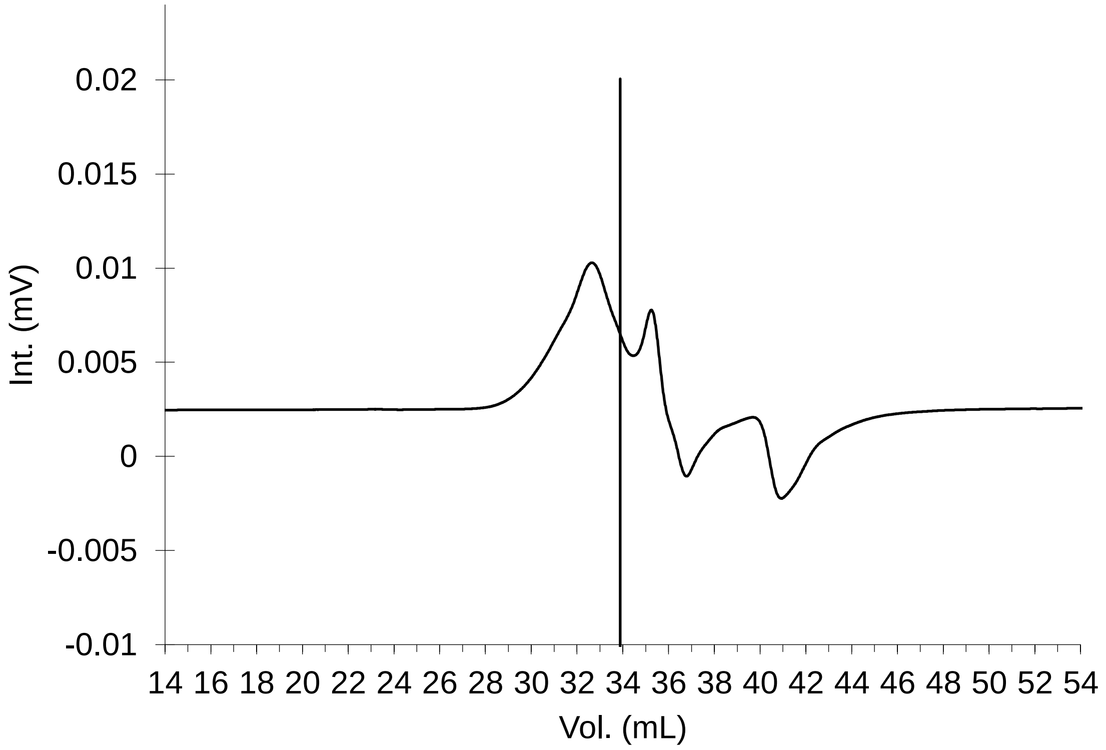

Figure S77. GPC traces of **BEDCU6**.

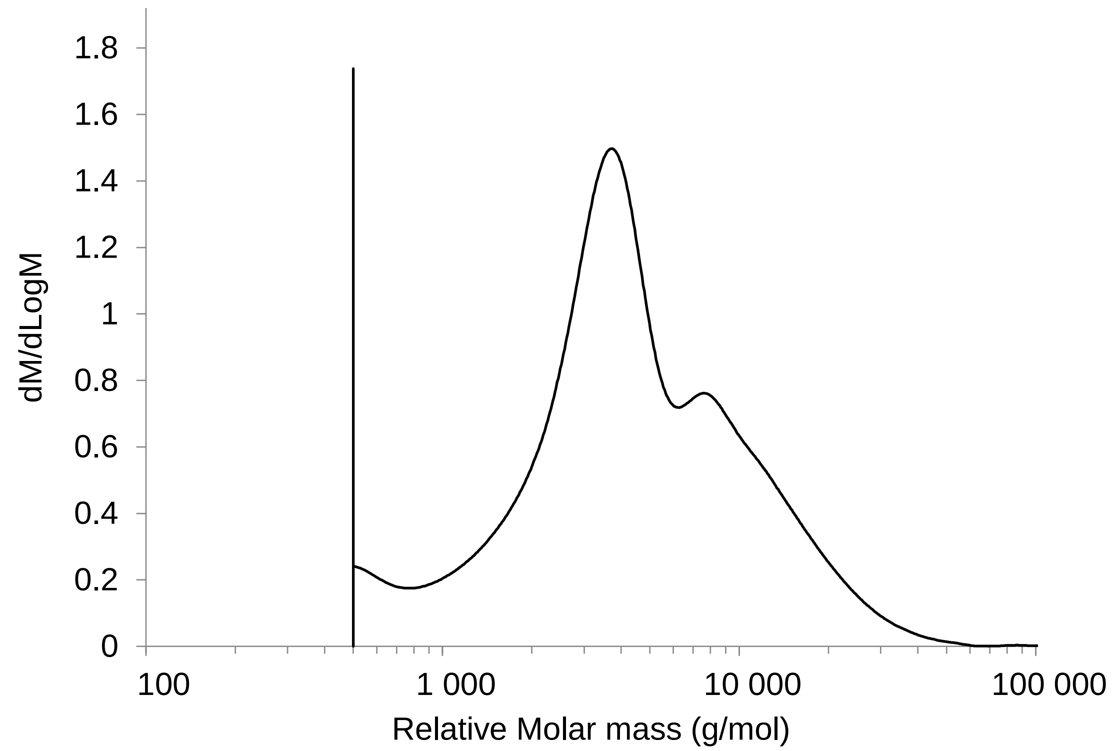

M_n [kDa]	*Đ*
2.0	1.6

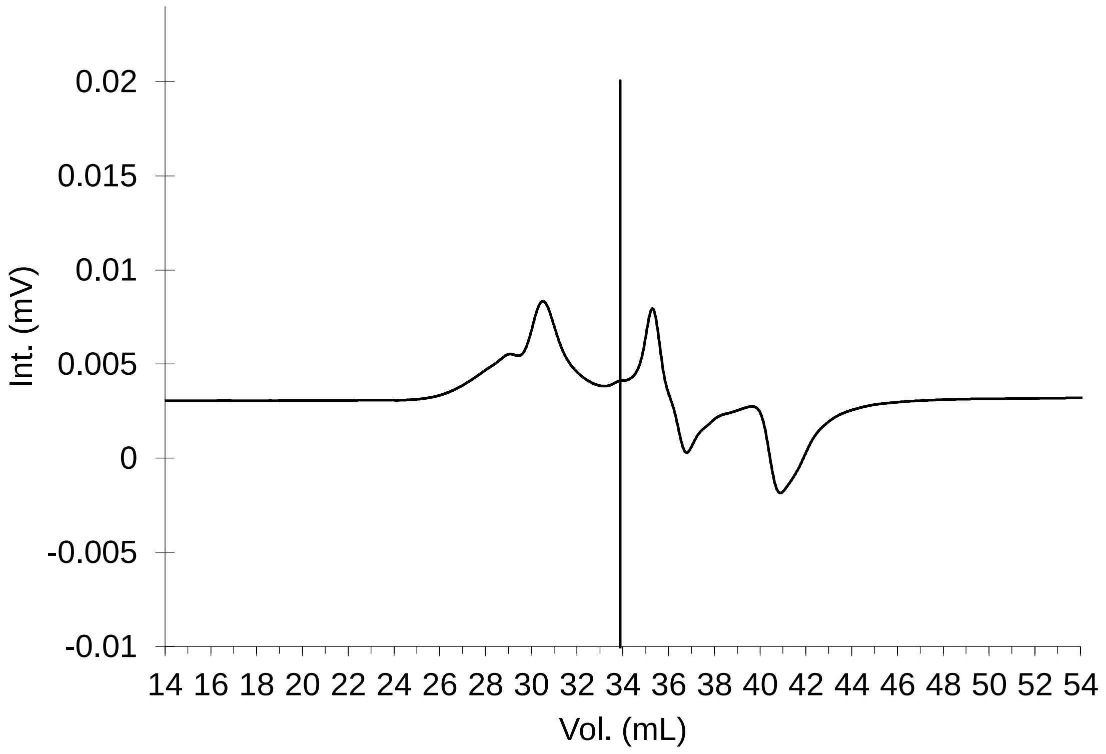

Figure S78. GPC traces of **BEDCU7**.

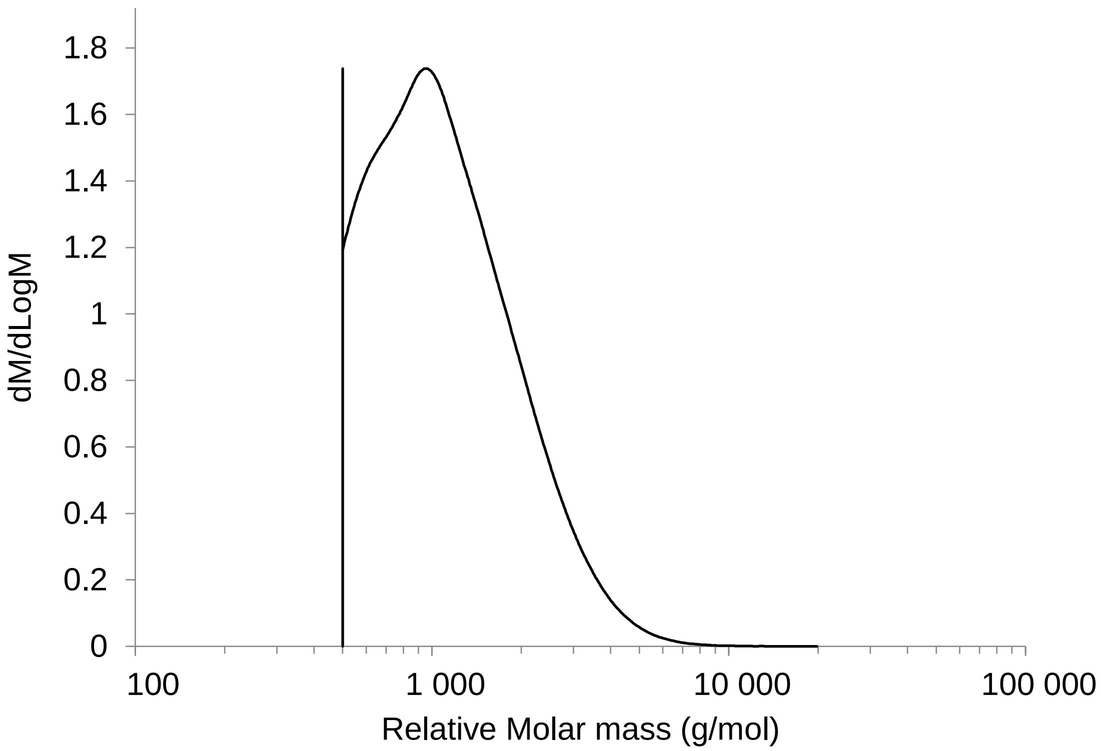

M_n [kDa]	*Đ*
6.3	2.1

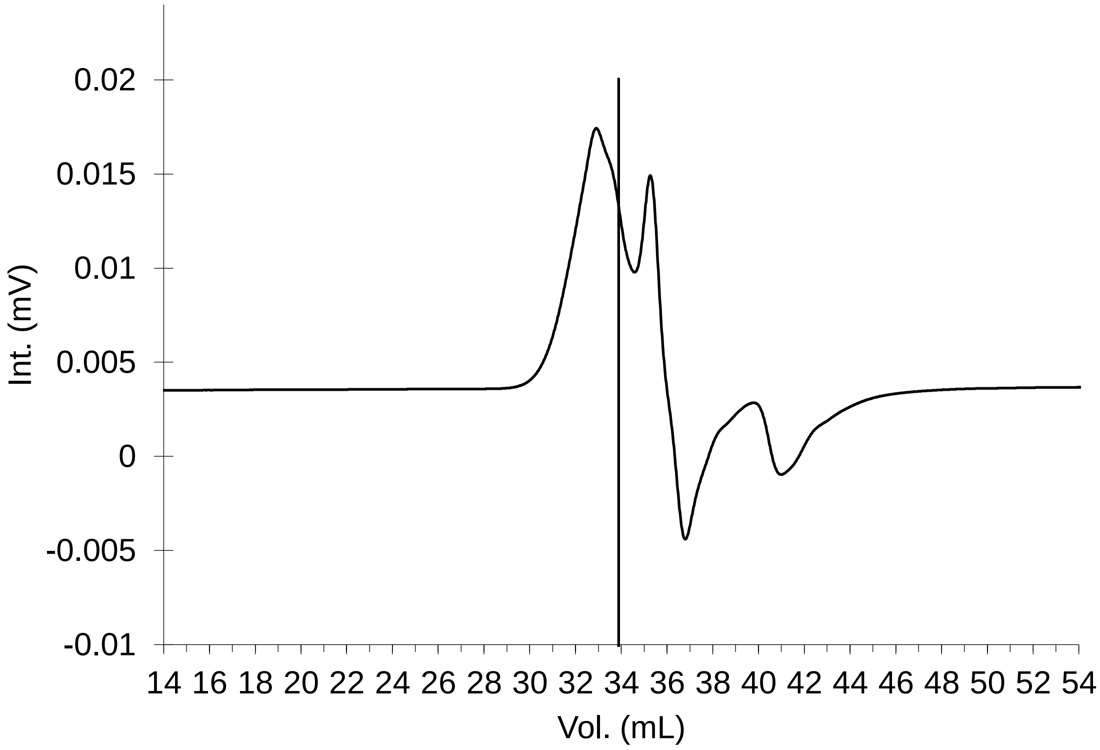

S75. DSC analyses

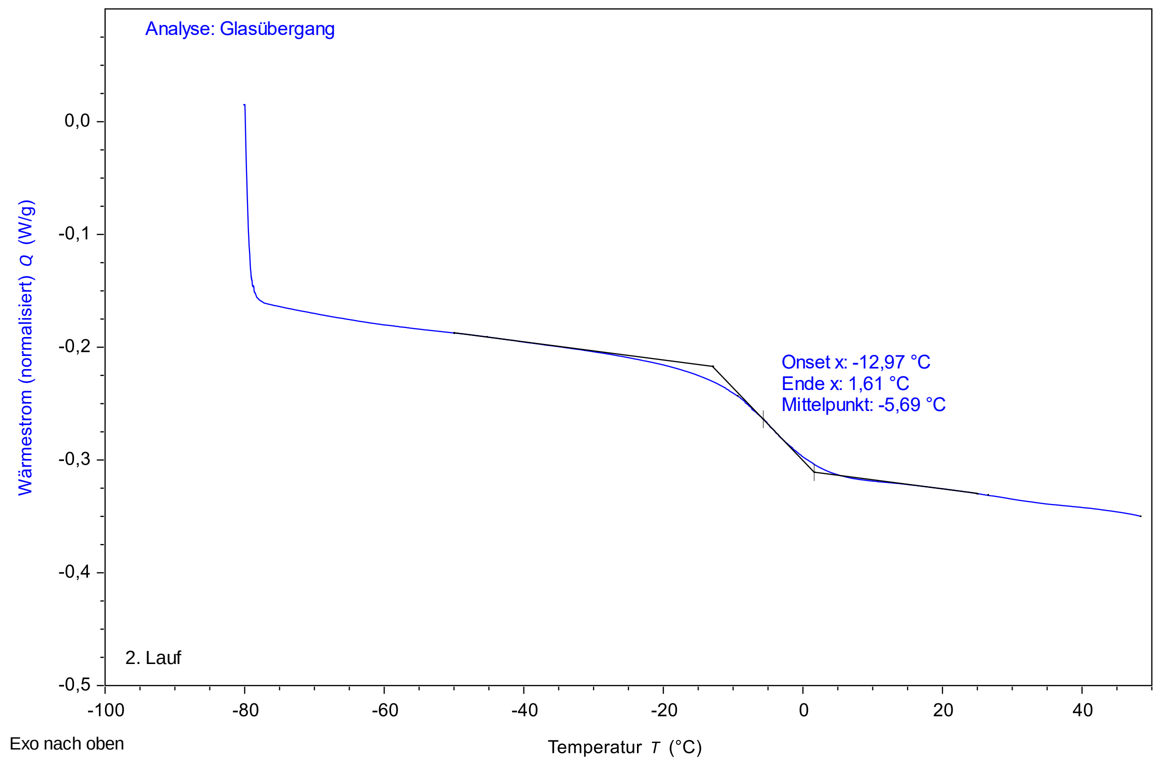

Figure S79. DSC thermogram of **BEDCU3**.

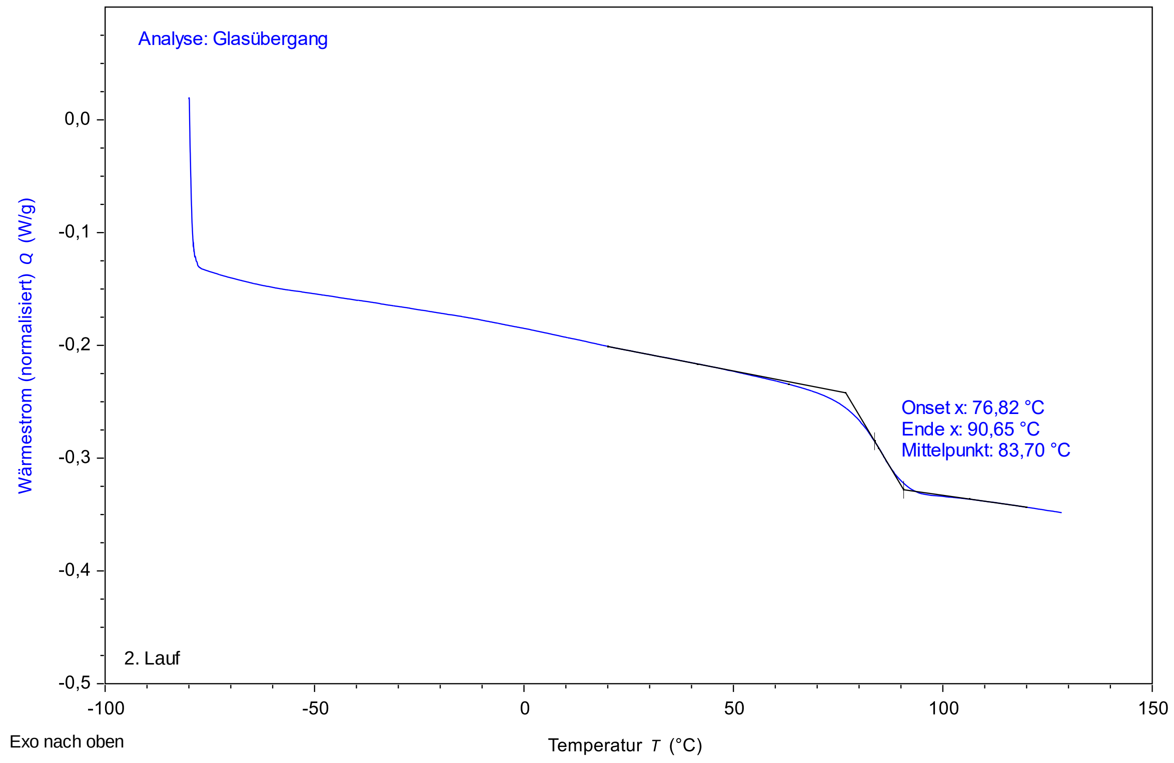

Figure S80. DSC thermogram of **BEDCU4**.

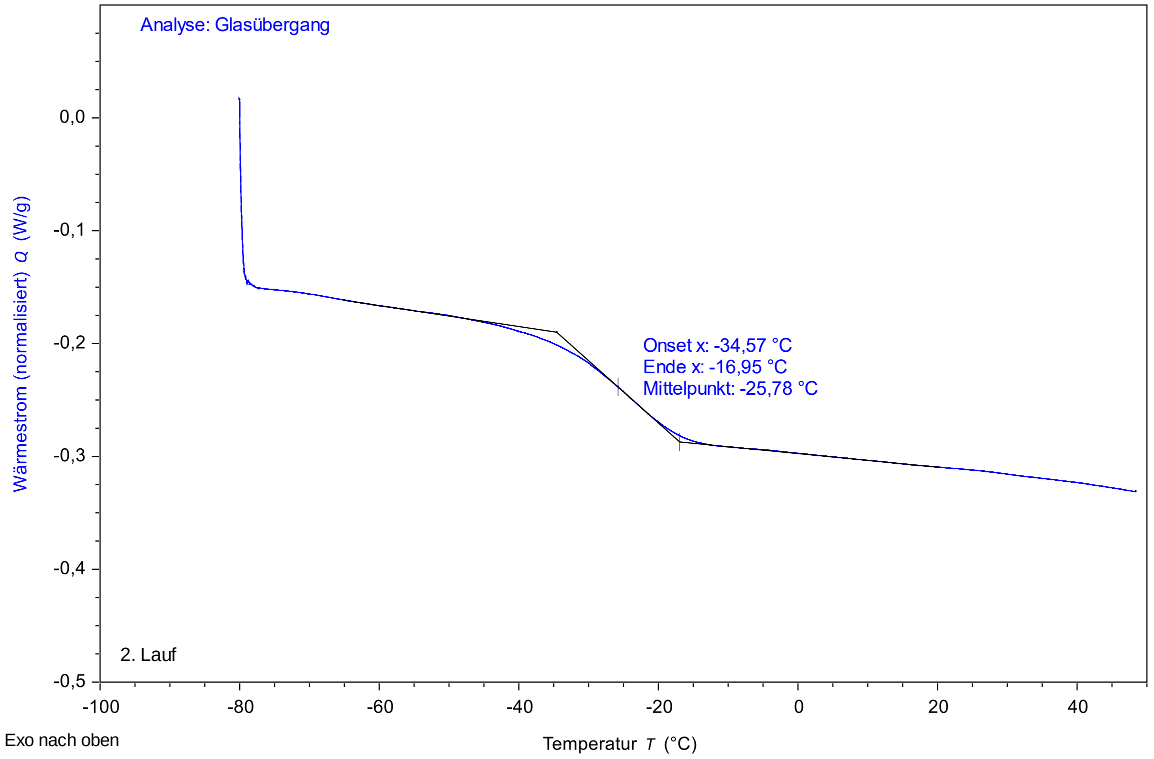

Figure S81. DSC thermogram of **BEDCU5**.

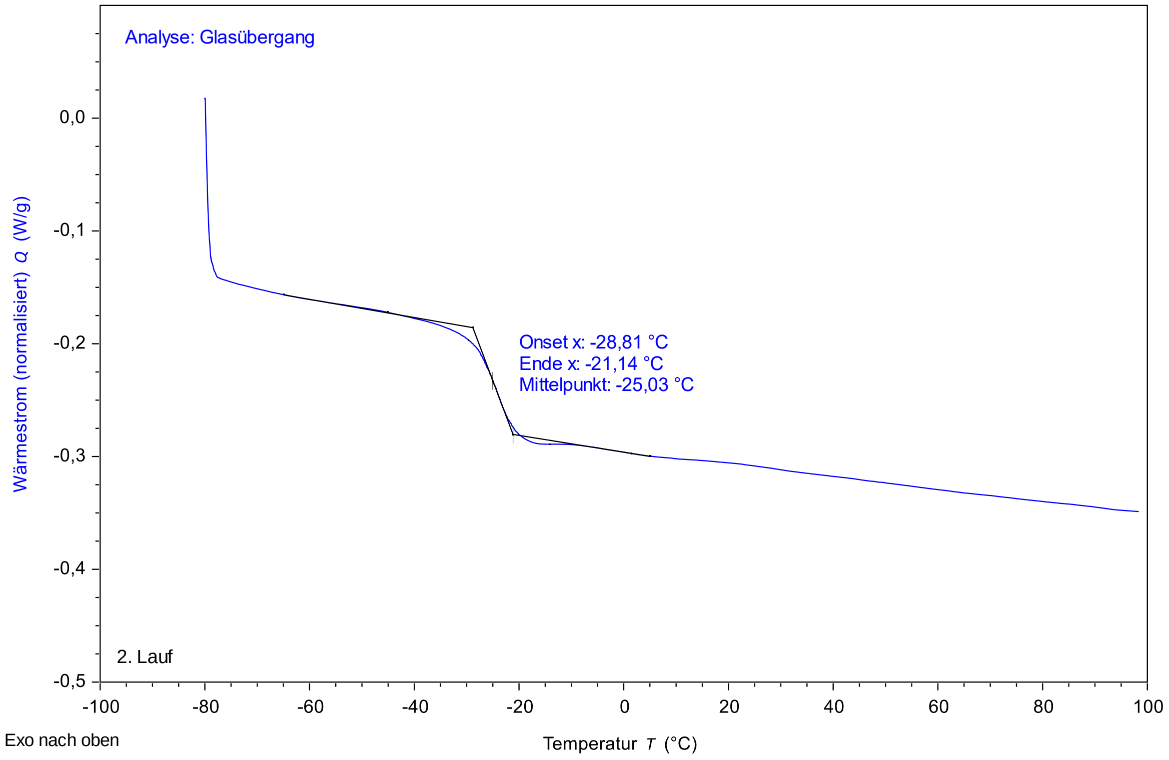

Figure S82. DSC thermogram of **BEDCU6**.

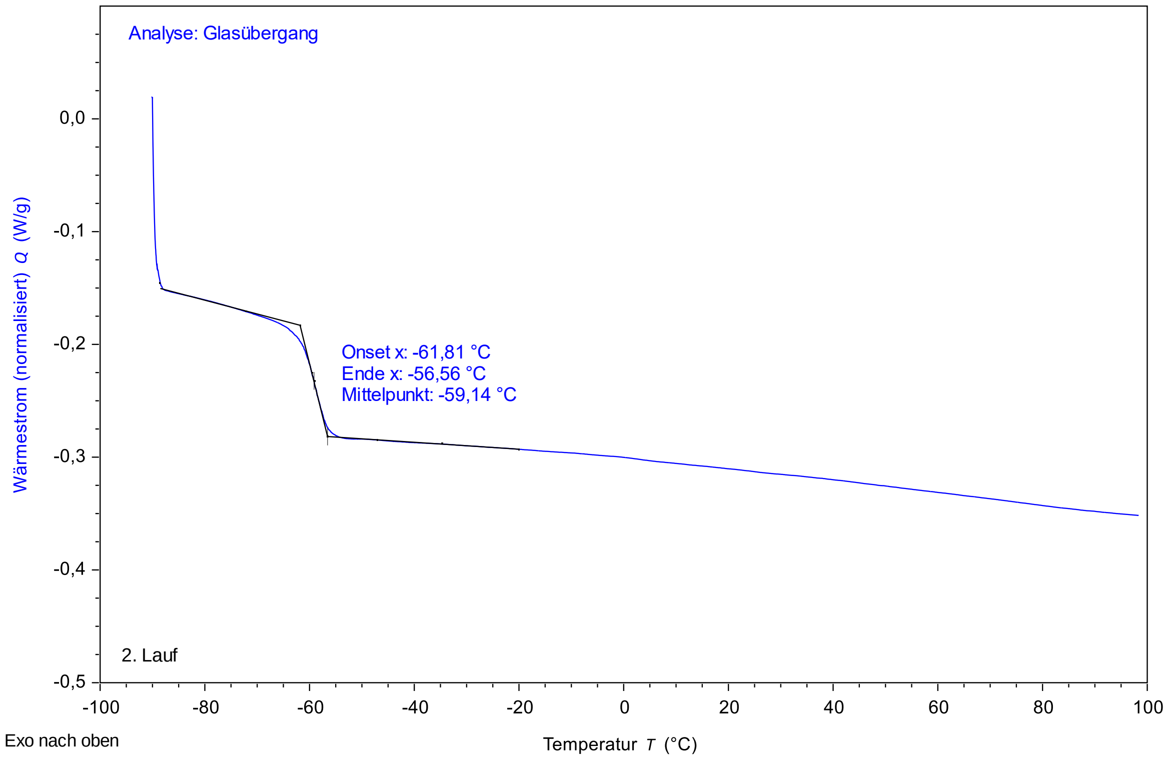

Figure S83. DSC thermogram of **BEDCU7**.

S78. Thermogravimetric analyses

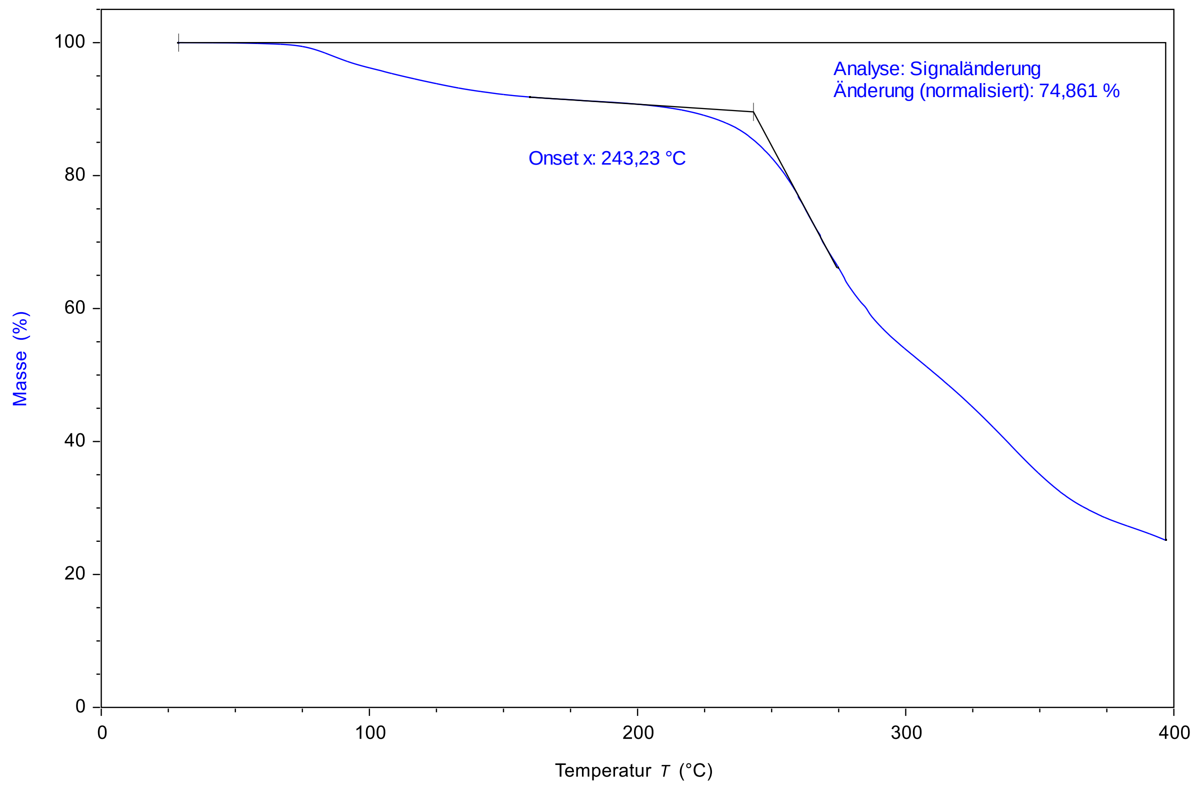

Figure S84. TGA thermogram of **BEDCU3**.

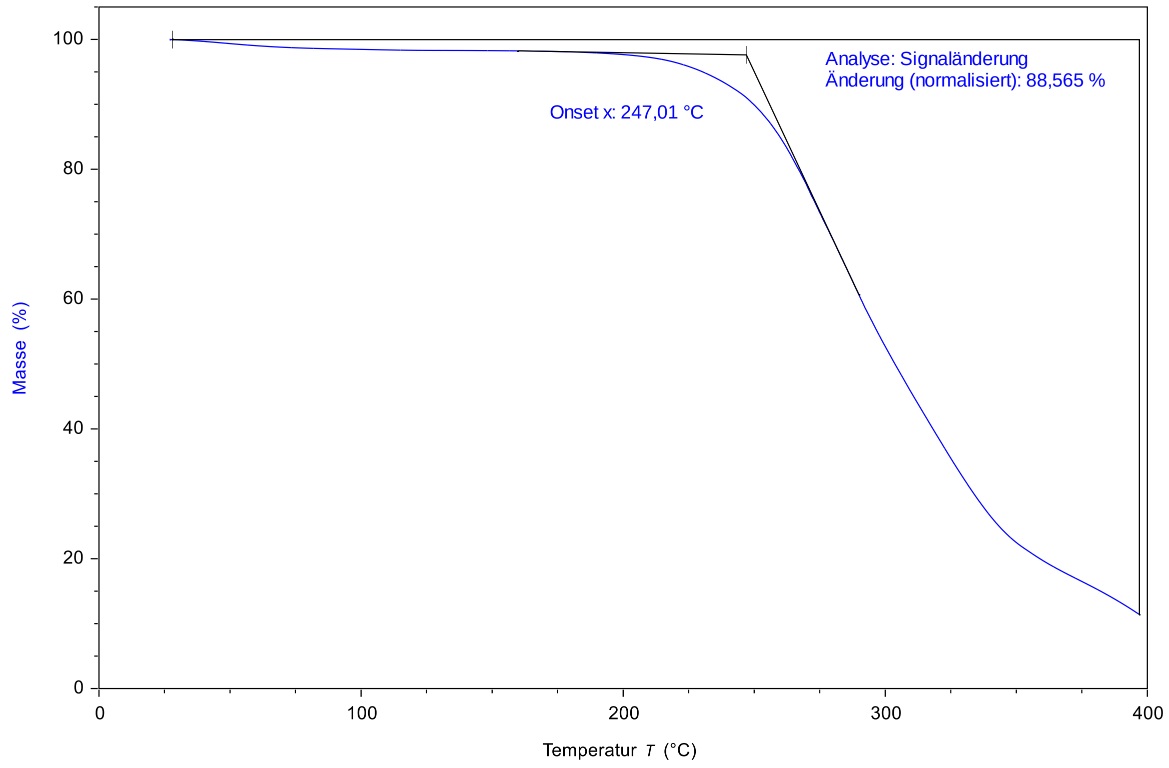

Figure S85. TGA thermogram of **BEDCU4**.

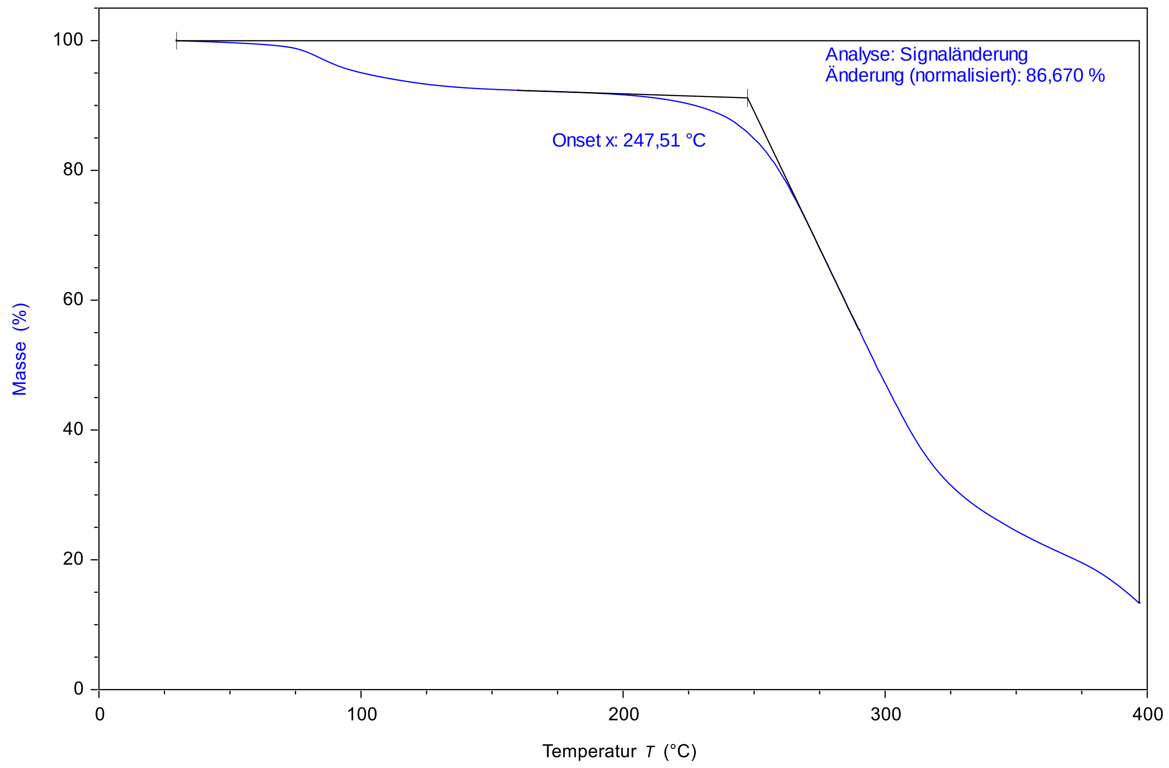

Figure S86. TGA thermogram of **BEDCU5**.

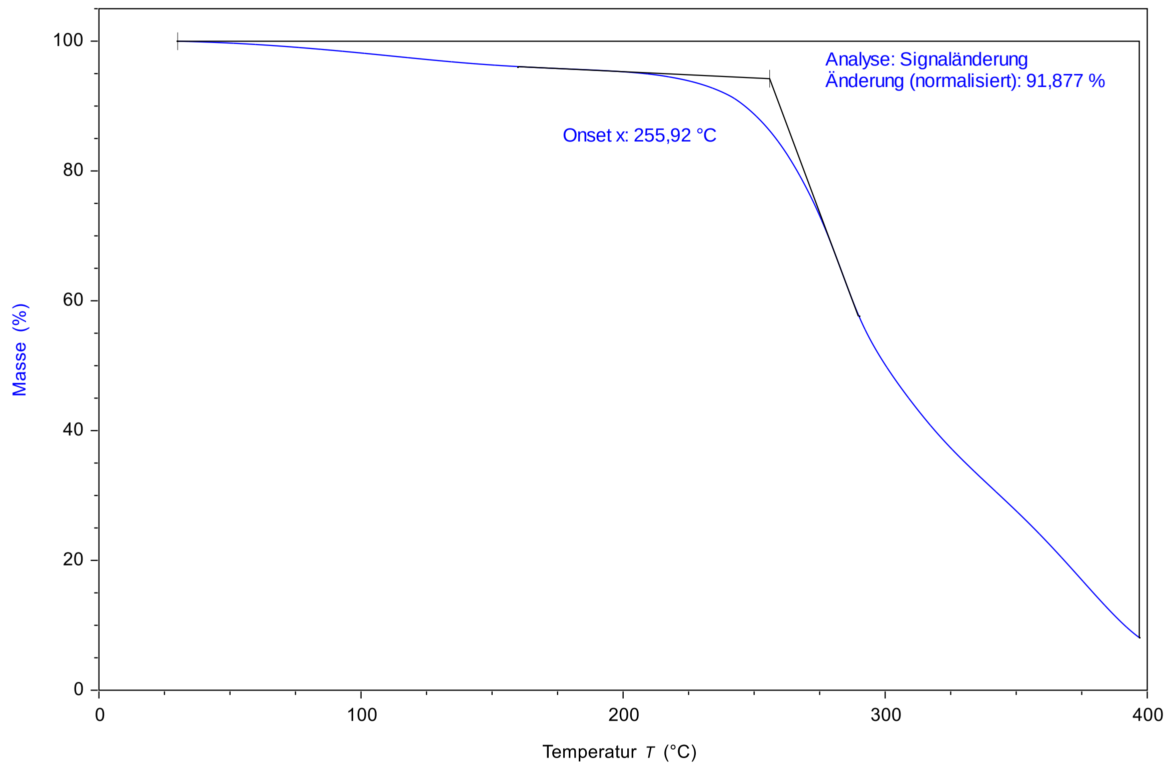

Figure S87. TGA thermogram of **BEDCU6**.

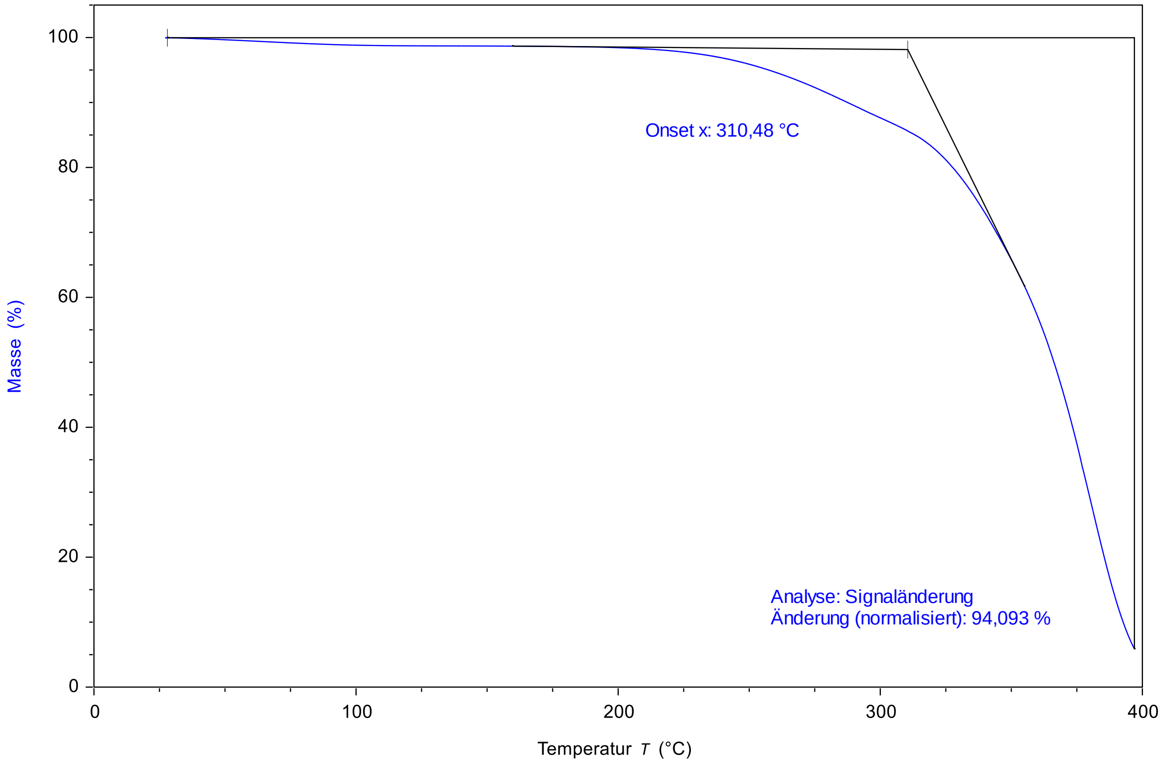

Figure S88. TGA thermogram of **BEDCU7**.

S81. References

[1] Della Monica, F; Kleij, A. W. Synthesis and Characterization of Biobased Polyesters with Tunable Tg by ROCOP of Beta-Elemene Oxides and Phthalic Anhydride. *ACS Sustainable Chem. Eng*. **2021**, *9*, 2619−2625.
